# Functional Specialisation and Diversity Shape the Rhizosphere Microbiota of Cacao and Coffee in the Amazonas Region, Peru

**DOI:** 10.1111/1758-2229.70259

**Published:** 2025-12-09

**Authors:** Jois V. Carrion, Maricela Chavez, Yadhira M. Olano, Martha S. Calderon, Danilo E. Bustamante

**Affiliations:** ^1^ Instituto de Investigación Para el Desarrollo Sustentable de Ceja de Selva (INDES‐CES) Universidad Nacional Toribio Rodríguez de Mendoza Chachapoyas Amazonas Peru; ^2^ Instituto de Investigación en Ingeniería Ambiental (INAM), Facultad de Ingeniería Ambiental, Biosistemas y de la Energía (FIABE) Universidad Nacional Toribio Rodríguez de Mendoza Chachapoyas Amazonas Peru

**Keywords:** Archaea, bacteria, *Coffea arabica*, functional role, fungi, microbiota diversity, *Theobroma cacao*

## Abstract

Cacao and coffee are the most economically important crops in the Amazonas region, Peru. Thus, characterising microbial species composition and functional profiles in these rhizospheric soils will enable a comprehensive representation of the metabolic potential of host plants in soils. Accordingly, rhizospheric soil from cacao and coffee farms was collected for both physicochemical and molecular analyses. The diversity and functional roles of the archaeal, bacterial and fungal communities were investigated through a comprehensive DNA metabarcoding analysis. It was found that each crop hosts distinct microbiomes, with a minimal shared core microbiota, underscoring the plant host's role as a primary filter. However, the assembly rules and functional responses diverged significantly across domains. Bacterial communities were compositionally and functionally homogeneous within each crop, shaped by deterministic factors. In contrast, archaeal and fungal compositions were heterogeneous but maintained stable functional profiles within their crop environment. Functionally, prokaryotes (bacteria and archaea) acted as “specialists,” exhibiting significant metabolic divergence between crops. Conversely, fungi served as “generalists,” showing no significant difference in guilds and providing a stable, redundant backbone for decomposition. This tiered microbial response highlights a fundamental ecological dichotomy and underscores the necessity of a multi‐kingdom perspective to fully understand and manage the rhizosphere ecosystem.

## Graphical abstract

The microbial species composition, abundance and functional profiles in the rhizospheric soils of cacao and coffee were evaluated. Our findings highlight the presence of predominant but functionally specialised taxa that play unique functional roles within the cacao and coffee rhizosphere ecosystems.

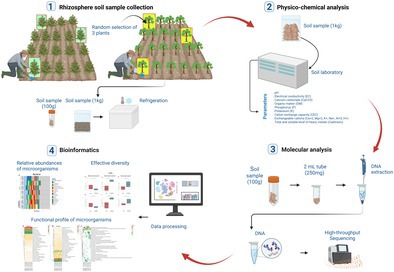

## 1 Introduction

Soil plays a critical role in supporting crop development through a number of essential functions (Powlson et al. 2011). It provides structural support to plant roots, supplies vital minerals and nutrients, protects against erosion and facilitates a range of physical, biological and chemical processes in the environment (Munawar and Wiryono 2019; Zhang et al. 2020). Soil health encompasses attributes such as resilience to stress, high biological diversity and efficient internal nutrient cycling (Van Bruggen and Semenov 2000). Soils with specific microbial diversity contribute to enhancing and regulating beneficial processes for plants, including nutrient uptake, nitrogen fixation, stress tolerance and overall growth promotion (Ali and Xie 2019; Kaviya et al. 2019). An increase in temperature of 2°C–3°C in 2050 is predicted to have a negative impact on soil microbial diversity and consequently on soil health by generating drought, salinisation and acidification (Ali and Xie 2019; Cruz‐Paredes et al. 2023; Zhao, Xie, et al. 2024; Zhao, Zhang, et al. 2024). This phenomenon will lead to disequilibrium in the diversity and composition of the soil microbial community, changing solute mobility and nutrient availability (Dutta and Dutta 2016; Ren et al. 2018; Yang et al. 2021). As a consequence, crops and other plants will be impacted by increased disease incidence and yield reduction (Jayaraman et al. 2021).

Understanding the composition and function of the microbiome in agricultural soils can provide valuable information on their ability to cope with climate impacts and promote crop health (Saleem et al. 2019). These microbiomes play key roles in nutrient availability, disease resistance and the maintenance of soil structure and fertility (Yadav et al. 2021; Todorović et al. 2023). For instance, the soil microbiota (i.e., archaea, bacteria and fungi) mineralizes soil nutrients, making them available to plants (Marzouk et al. 2024). By secreting sticky polysaccharides, these microorganisms bind soil particles together, preventing erosion (Costa et al. 2018). They also coregulate the hormonal balance of plants, helping them cope with abiotic stressors and providing protection against a variety of insect pests, parasites and pathogens (Saleem et al. 2019). They also play roles in promoting plant growth through auxin biosynthesis (Taffner et al. 2018). Accordingly, the characterisation of microbiomes, especially in poorly studied soils with their own features, such as those from the Amazonas region in northern Peru, is highly important in the context of climate change (Mukhtar et al. 2023). Modern molecular tools like metabarcoding use high‐throughput sequencing of specific gene markers to simultaneously identify thousands of microbial species and their abundance within a sample (Ríos‐Castro et al. 2021). It is widely applied in microbial ecology, using markers like 16S rRNA for prokaryotes and ITS for fungi to profile communities in environments such as soil (de Groot et al. 2021; Maretto et al. 2022).

It is imperative to characterise the crucial role of microbial communities in the transformation of organic matter and nutrient cycling in the soil (Piotrowska‐Długosz et al. 2022). To achieve this, methodologies such as functional inference have become necessary when using metabarcoding data (Djemiel et al. 2022). The determination of functional profiles allows the representation of metabolic capabilities on the basis of the presence or absence of genes associated with those functions (Laroche et al. 2021). Methods for functional inference are based on the assumption that the phylogenetic information of gene sequences correlates closely with the genomic content, which allows accurate predictions when reference genomes are available (Djemiel et al. 2022). For functional profiling, specialised databases such as PICRUSt (Phylogenetic Investigation of Communities by Reconstruction of Unobserved States, https://picrust.github.io/picrust/) (Yang, Mai, et al. 2023; Yang, Qiu, et al. 2023; Vigil et al. 2024) and FUNGuild (Nguyen et al. 2016) have been widely adopted. For instance, several studies have used PICRUSt to (i) determine the downregulation of genes involved in propionate and butyrate oxidation in an acidogenic bioreactor (Li et al. 2022), (ii) characterise the metabolic behaviour of bacteria during silage fermentation (Wang et al. 2022) and (iii) highlight 44 key metabolic pathways associated with bacterial metabolism and genetic information processing hosted by macroalgae (Vigil et al. 2024). The FUNGuild tool assigns ecological guilds on the basis of the taxonomic classification of fungi using the ITS marker and provides insight into the potential functional roles of fungal communities (Nguyen et al. 2016). For instance, the use of FUNGuild allowed the identification of a greater richness of pathotroph, symbiotroph and saprotroph microhabitats closer to coffee plants (Rao et al. 2020).

Cacao and coffee are the most economically important crops in the Amazonas region, northern Peru (MINAGRI 2017). Specifically, cacao production in the Amazonas represents approximately 3.6% of total production in Peru (MINCETUR 2023). The unique sensory qualities, such as flavour and aroma, of the cacao from the Peruvian Amazonas region led to a designation of origin, namely, Fine Aroma Cacao (INDECOPI 2016; Bustamante et al. 2022), making this cacao highly attractive to international markets (INDECOPI 2016). Similarly, coffee is the first export commodity of the Amazonas region, and its production represents 6.3% of the national coffee exports after San Martin, Cajamarca and Junin regions (MINCETUR 2023). On the basis of this relevance, both cacao and coffee crops from the Amazonas region have been extensively investigated with respect to agronomical (Rojas‐Briceño et al. 2022; Oliva et al. 2023), physiological (Gonzales Alegría et al. 2023), genetic (Bustamante et al. 2022; Meléndez‐Mori et al. 2023), genomic (Calderon et al. 2024) and organoleptic (Oliva‐Cruz et al. 2022) topics. Despite this abundance of information, there is a lack of knowledge about the rhizospheric soil microbiomes of cacao and coffee crops. Accordingly, this study aimed to decipher the microbial biodiversity and determine the functional profile of the microbiota inhabiting cacao and coffee rhizospheric soils in the Amazonas region. This study elucidated the diversity and metabolic behaviour of the rhizospheric soil microbial community in cacao and coffee.

## 2 Materials and Methods

### 2.1 Rhizospheric Soil Sampling

The provinces of Bagua and Utcubamba are well known for their production of Fine Aroma Cacao (Castañeda Chávez et al. 2018), whereas the provinces of Luya and Rodríguez de Mendoza produce specialty coffees (Salas López et al. 2020). The collection of rhizospheric soil samples associated with *Theobroma cacao* crops was carried out in three districts of Bagua (−5.437249, −78.442347; 420 masl) and one district of Utcubamba (−5.742306, −78.295304; 465 masl) (Table S1). In contrast, rhizospheric soil sampling from *Coffea arabica* crops under direct sunlight was carried out in five districts of Luya for the Caturra variety (−6.274361, −78.172223; 2303 masl) and two districts of Rodríguez de Mendoza for the Typica variety (−6.541572, −77.410553; 1597 masl) (Table S1).

**FIGURE 1:**
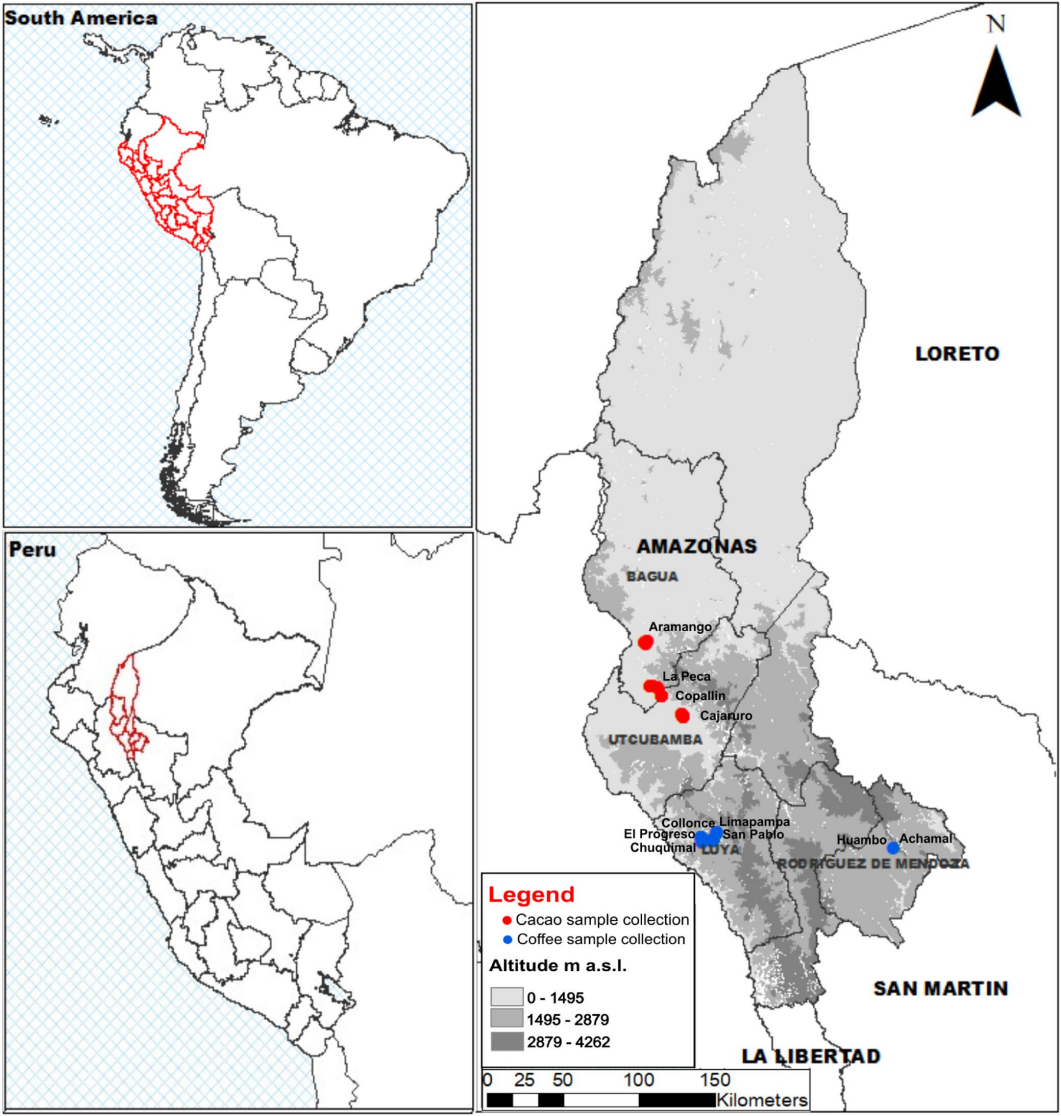
Sampling points of rhizospheric soil from cacao (red dots) and coffee (blue dots) crops in the provinces of Bagua, Luya, Utcubamba and Rodríguez de Mendoza.

The sampling approach was nonrandom convenience sampling, and plots whose owners were willing to participate in the research were selected (Figure 1). The collection procedures for soil sampling adhered to the sampling guidelines established by MINAM (2014). Briefly, the samples were collected from the rhizospheric zone of the cacao and coffee plants at a depth of 20 cm. Four samples separated by 1 m were taken per plant and subsequently homogenised to obtain approximately 2 kg of rhizospheric soil. All samples were immediately stored at 4°C in coolers for preservation, and upon arrival at the laboratory, they were refrigerated and stored at −20°C in the Molecular Biology and Genomics Laboratory of the Universidad Nacional Toribio Rodríguez de Mendoza (UNTRM) (Figure S1). These rhizospheric soil samples were used for both physicochemical and molecular analyses.

### 2.2 Physicochemical Properties of the Rhizospheric Soil

The physicochemical analyses of the rhizosperic soil were performed commercially at the Laboratory of Chemical Analysis of Soil, Plant and Water at Universidad Nacional Agraria La Molina (Lima, Peru), according to standardised methods. The parameters evaluated were pH, electrical conductivity (EC), calcium carbonate (CaCO_3_), organic matter (OM), phosphorus (P), potassium (K), cation exchange capacity (CEC), exchangeable cations (Ca^+2^, Mg^+2^, K^+^, Na^+^, Al^+3^ and H^+^), textural classes (sand, silt and clay) and total and soluble heavy metal levels (cadmium) (Figure S2).

### 2.3 DNA Extraction and Amplicon Sequencing

#### 2.3.1 DNA Extraction

Genomic DNA was extracted from each sample core with ZymoBIOMICS (Irvine, CA, USA) and pooled prior to sequencing. One round of extraction and pooling allowed enough DNA to be obtained (pooled samples included DNA from four extractions). Genomic DNA extraction of microorganisms from rhizospheric soil samples was performed according to the manufacturer's instructions. The quality of the extracted DNA was assessed on agarose gels (1%) and using the Qubit dsDNA BR Assay Kit (Invitrogen, Carlsbad, CA) and NanoDrop (Thermo Fisher Scientific, Waltham, MA, USA).

#### 2.3.2 Library Preparation and Sequencing

Library construction and Illumina sequencing using the MiSeq platform were performed commercially by IGATech (Udine, Italy). Genomic DNA was quantified through optical density measurements via a Qubit instrument (Thermo Fisher Scientific, Waltham, MA, USA). DNA quality was assessed by agarose gel electrophoresis and an Agilent TapeStation. Libraries were prepared via the Illumina Metagenomic Sequencing Library Preparation Protocol (https://support.illumina.com/documents/documentation/chemistry_documentation/16s/16s‐metagenomic‐libraryprep‐guide‐15044223‐b.pdf) in two amplification steps: initial PCR amplification using locus‐specific PCR primers and subsequent amplification that integrates relevant flow‐cell binding domains and unique indices (NexteraXT Index Kit, FC‐131‐1001/FC‐131‐1002). The resulting library was validated for DNA size distribution and concentration using a Qubit instrument and a TapeStation. For the identification of bacterial communities, the V3–V4 region of the 16S rRNA gene was amplified and sequenced using the specific primers 341F (forward: 5′‐CCTACGGGNBGCASCAG‐3′) and 805R (reverse: 5′‐GACTACNVGGGTATCTAATCC‐3′) (Wasimuddin et al. 2020). For the archaeal communities, the 16S rRNA gene was amplified and sequenced using the primers Arch340wF (forward: 5′‐CCCTAYGGGGGGYGCASCAG‐3′) and Arch806R (reverse: 5′‐GGACTACVSGGGTATCTAAT‐3′) (Klindworth et al. 2013). For the fungal communities, the ITS gene was amplified and sequenced using the primers ITS1 (forward: 5′‐TCCGTAGGTGAACCTGCGG‐3′) and ITS2 (reverse: 5′‐GCTGCGTTCTTCATCGATGC‐3′) (White et al. 1990). Deep metagenome amplicon sequencing was performed, generating 250 base‐paired (bp) reads each.

### 2.4 Bioinformatics

The reads were processed as follows: (i) the reads maintained their 250 bp length (Phred score > 20), (ii) ambiguous or undefined bases (Ns) were not allowed and (iii) reads with a quality score < 20 were removed. The obtained sequences were processed using the Dada2 package version 1.26.0 (Callahan et al. 2016) for quality filtering and chimaera removal, followed by amplicon sequence variant (ASV) inference. For taxonomic classification, the SILVA database (Release 138) (Quast et al. 2013) was used for prokaryotes through a naive Bayes RDP classifier with 95% similarity, an 8‐size kmer and 100 bootstrap replicates, whereas the Unite v8.3 database (Nilsson et al. 2019) was employed for fungi. The compositions of the bacterial, archaeal and fungal communities were presented as relative abundance data, and the most abundant microbial taxa were presented at different levels (phylum, class and genus) for each collection site (districts and provinces) and for each crop type (cacao and coffee), following the default parameters of the microViz package, version 0.10.08 (Barnett et al. 2021). Venn diagrams were constructed to visualise the microbiota shared among sampling districts for each crop individually, as well as for both crops combined. Additionally, co‐occurrence networks for bacteria, archaea and fungi for each crop were modelled using the microeco package to determine potential ecological interactions. The alpha diversity indices (i.e., Shannon and Simpson indices) were calculated for each crop (cacao or coffee) and province via the diversity function of the Phyloseq package version 1.42.0 (McMurdie and Holmes 2013). Hill numbers were also calculated to facilitate a comparison of diversity between the two crops (Jost 2007).

For beta diversity, the structures and similarities between microbial communities at the collection sites were analysed using the Bray–Curtis dissimilarity matrix for principal coordinate analysis (PCoA) using the vegan package version 2.6–4 (Oksanen et al. 2007). For multivariate analysis, discriminant analysis of principal components (DAPC) was performed using the package Adegenet version 1.3.1 (Jombart and Ahmed 2011). A PERMANOVA model was implemented on this multivariate analysis to determine the influence of crop. Additionally, using a PERMANOVA model, db‐RDA (distance‐based redundancy analysis) was performed to identify the influence of environmental variables and crop type on microbial composition. All packages used in this analysis were run in R software version 4.2.2 (Ramette 2007).

### 2.5 Functional Profile of the Microbiota

The PICRUSt2 software was used to understand the potential functional genetic capabilities of the identified bacterial and archaeal communities (Douglas et al. 2020). This analysis was performed using non‐rarefied data and decontaminated ASVs to maintain the integrity of the original distribution, allowing an accurate representation of the abundance of predicted gene families (Davis et al. 2018). These data were then rigorously filtered to identify KEGG orthology (KO) markers using a threshold of 1000 counts after normalisation to counts per million (cpm) (Robinson et al. 2010). Gene family predictions were combined with the relative abundance of genes in the samples. The data were visualised using ggpicrust2 version 1.7.2 (Yang, Mai, et al. 2023; Yang, Qiu, et al. 2023) in R version 4.2.2 to interpret the functional profile of the microbiota associated with cacao and coffee rhizospheric soils.

For fungi, functional guilds were assigned using the FUNGuild tool in R version 4.4.2 (Nguyen et al. 2016). To ensure comparability between samples with different sequencing depths, functional abundances were normalised to the minimum total observed across samples. This strategy preserves all available functional information and faithfully represents the relative functional composition of each sample. Thus, this allows a statistically valid comparison of ecological patterns between localities (Nguyen et al. 2016).

## 3 Results

### 3.1 Characterisation of Illumina Sequencing Data

The number of unprocessed reads for the cacao rhizospheric soils was 5,096,510 for 16S rRNA (Bacteria), 4,696,030 for 16S rRNA (Archaea) and 2,961,680 for ITS (fungi) (Table S2). For the coffee rhizospheric soils, a total of 1,269,326 for 16S rRNA (Bacteria), 4,459,372 for 16S rRNA (Archaea) and 1,339,324 for ITS (fungi) unprocessed reads were obtained (Table S2). All these reads had an average length of 250 bp. After quality and filtering analysis of the forward and reverse reads, clean sequence reads with Phred score values above 20 were obtained (Figures S3 and S4, Table S2). The chimeric, plastid and mitochondrial sequences were filtered out to obtain effective sequence reads for subsequent analyses (Table S2).

### 3.2 Relative Abundance of the Microbiota Associated With Cacao and Coffee Rhizospheric Soils

**FIGURE 2:**
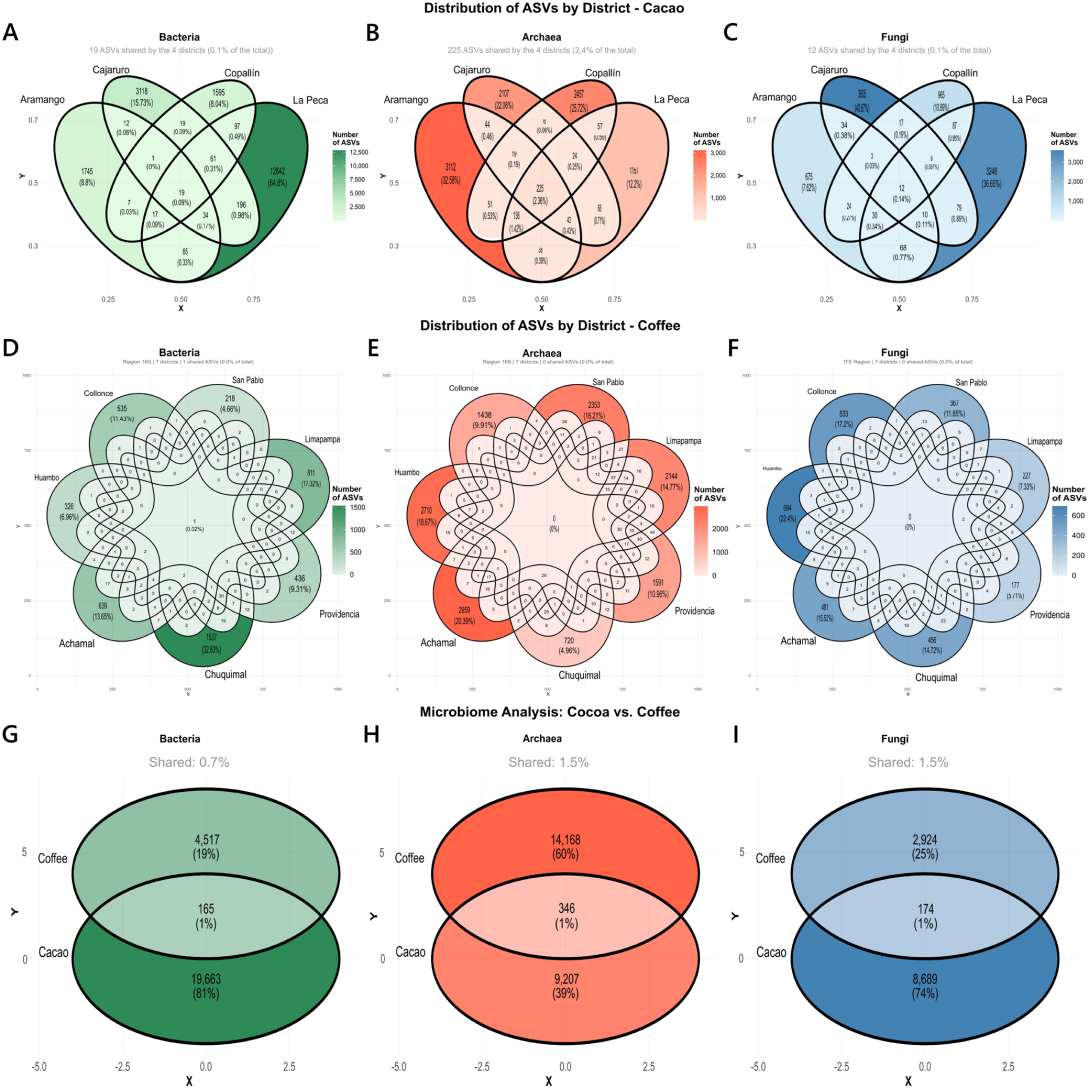
Distribution of ASVs in cacao and coffee rhizosphere microbiota. Venn diagrams illustrate the unique and shared ASVs across sampling districts for cacao (A–C) and coffee (D–F). Global comparison between microbiota from cacao and coffee rhizospheres showing shared and exclusive ASVs (G–I).

The number of effective ASVs after normalisation for the microbiota of the cacao rhizospheric soils was 19,828 for bacteria, 9553 for archaea and 8863 for fungi. Our analysis revealed a predominance of unique ASVs and a low proportion of shared taxa across the four evaluated districts (i.e., Aramango, Cajaruro, Copallín and La Peca) in cacao rhizospheric soils. A core microbiome common to all four districts consisted of only 19 bacterial ASVs (0.09%), 225 archaeal ASVs (2.36%) and 12 fungal ASVs (0.14%) (Figure 2A–C). For the microbiota of the coffee rhizospheric soils, the number of effective ASVs after normalisation was 4682 for bacteria, 14,514 for archaea and 3098 for fungi. The microbial composition was even more heterogeneous, with a minimal presence of shared taxa. A core microbiome across all districts was identified for only one bacterial ASV (0.02%), while no core ASVs were detected for archaea or fungi (Figure 2D–F). Further analysis of the global microbiota from cacao and coffee rhizospheric soils revealed marked differences in microbial composition. Most ASVs were crop‐specific, confirming a clear differentiation between the cacao and coffee rhizospheres. Only 165 bacterial ASVs (1%), 346 archaeal ASVs (1%) and 174 fungal ASVs (1%) were shared between the two crops (Figure 2G–I).

**FIGURE 3:**
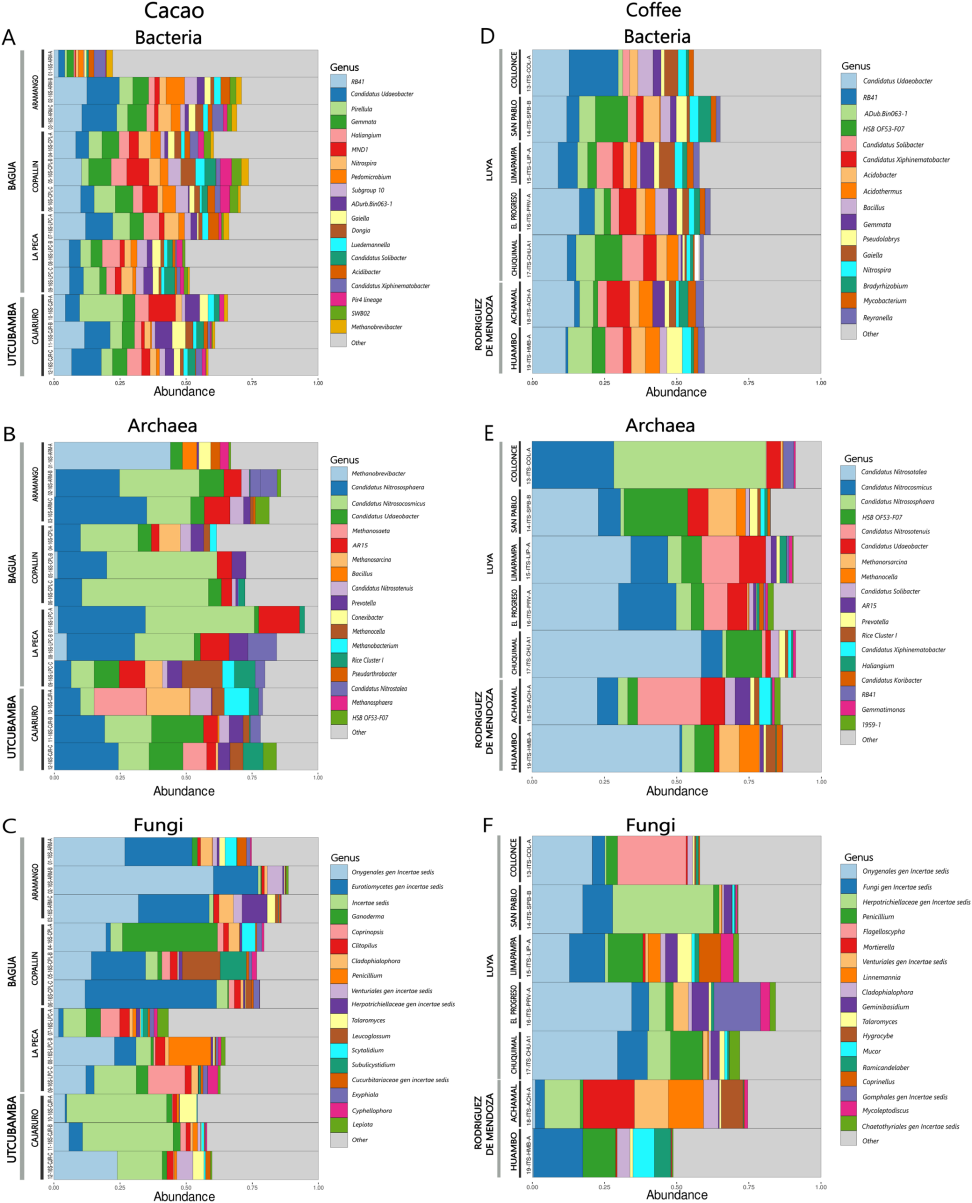
Genus‐level taxonomic composition of the rhizosphere microbiota. Panels show bacteria (A, D), archaea (B, E) and fungi (C, F) associated with cacao soils from Bagua and Utcubamba (A–C) and coffee soils from Luya and Rodríguez de Mendoza (D–F).

For the microbiome associated with cacao rhizospheric soils, the relative abundances of archaea and fungi differed across districts and provinces, especially at the genus level (Figure 3). Conversely, the relative abundance of bacteria exhibited similar compositions, except for a sample from Aramango (ARM‐A), which was notably distinct.

A total of 24 bacterial phyla were identified in the provinces of Bagua and Utcubamba. The most abundant phyla were Proteobacteria (23.5%), Planctomycetota (15.5%), Verrucomicrobiota (15.2%) and Acidobacteriota (13.9%) (Figure S5A). At the class level, 49 bacterial classes were identified, and the most abundant were Verrucomicrobiae (14.9%), Planctomycetes (13.6%), Gammaproteobacteria (12.0%) and Alphaproteobacteria (11.5%) (Figure S5B). At the genus level, 2323 bacterial genera were identified. The most abundant genera were *Candidatus Udaebacter* (8.4%), RB41 (6.70%), *Gemmata* (5.2%) and *Haliangium* (4.4%) (Figure 3A). A distinct bacterial composition was observed in a sample from Aramango (ARM‐A) in the relative abundance at the phylum (e.g., Actinobacteriota, Chloroflexi and Firmicutes), class (e.g., Ktedonobacteria and Thermoleophilia) and genus (e.g., *ADurb.Bin063‐1*) levels.

A total of 22 archaean phyla were identified. The most abundant phyla were Crenarchaeota (31.4%), Halobacterota (15.7%) and Euryarchaeota (9.3%) (Figure S5C). A distinct archaeal phylum, Euryarchaeota, was observed in a sample from Aramango (ARM‐A) (Figure S5C). At the class level, 37 archaean classes were identified, with Nitrososphaeria (30.8%), Methanobacteria (9.29%) and Methanosarcina (8.1%) being the most abundant (Figure S5D). At the genus level, 149 archaeal genera were identified. The most abundant genera were *Candidatus Nitrososphaera* (15.2%), *Candidatus Nitrosocosmicus* (8.1%) and *Methanobrevibacter* (6.7%) (Figure 3B).

A total of 11 fungal phyla were identified (Figure S5E). The most abundant phyla were Ascomycota (33.8%), Fungi phy Incertae sedis (30.5%) and Basidiomycota (29.0%). At the class level, 42 fungal classes were identified, and the most abundant were Eurotiomycetes (19.6%), Dothideomycetes (5.9%), Agaricomycetes (2.7%), Orbiliomycetes (2.7%), Sordariomycetes (2.1%) and Pezizomycetes (1.7%) (Figure S5F). At the genus level, 302 fungal genera were identified, and *Fungi gen Incertae sedis* (30.5%), *Onygenales gen Incertae sedis* (5.7%), *Eurotiomycetes gen Incertae sedis* (2.6%) and *Coprinopsis* (1.7%) were the most abundant (Figure 3C). A sample from Copallin presented a different abundance of *Ganoderma*, and the genus *Fungi gen Incertae sedis* was more intense in Cajaruro (Figure 3C).

For the microbiome associated with coffee rhizospheric soils, the relative abundance of bacteria exhibited similar compositions across districts and provinces. Conversely, the relative abundances of archaea and fungi differed, specifically at the genus level (Figure 3).

A total of 19 bacterial phyla were identified in the provinces of Luya and Rodríguez de Mendoza. The most abundant bacterial phyla were Verrucomicrobiota (30.64%), Proteobacteria (19.8%), Acidobacteriota (11.4%) and Actinobacteriota (9.3%) (Figure S6A). At the class level, 39 bacterial classes were identified. The most abundant classes were Verrucomicrobiae (30.4%), Alphaproteobacteria (10.1%), Gammaproteobacteria (9.7%) and Ktedonobacteria (9.0%) (Figure S6B). At the genus level, 192 bacterial genera were identified, and the most abundant were *Candidatus Udaebact* (20.64%), *HSB OF53‐F07* (5.5%), *RB41* (4.80%), *Gemmata* (5.2%) and *ADurb. Bin063‐1* (4.5%) (Figure 3D).

A total of 23 archaean phyla were identified (Figure S6C). The most abundant phyla in most samples were Crenarchaeota (46.7%), Halobacteria (15.8%) and Nanoarchaeota (1.7%). At the class level, 35 archaeal classes were identified. The most abundant were Nitrososphaeria (43.2%), Methanocellia (7.6%), Methanosarcinia (7.6%) and Nanoarchaeia (1.7%) (Figure S6D). At the genus level, 129 archaeal genera were identified. The most abundant included *Candidatus Nitrosotalea* (15.1%), *Candidatus Nitrososphaera* (13.70%), *HSB OF53‐F07* (10.02%) and *Candidatus Nitrosocosmicus* (8.1%) (Figure 3E). In addition, other notable genera, such as *Methanosarcina* (7.5%), *Candidatus Nitrosotenuis* (6.0%), *Methanocella* (4.2%) and *Rice Cluster I* (3.4%), were reported. In a sample from San Pablo (SPB‐1), a different composition of archaeal abundance was observed, with the genera *HSB OF53‐F07* and *Methanorcisina* being the most predominant (Figure 3E).

A total of 11 fungal phyla were identified. The most abundant phyla were Ascomycota (41.9%), Basidiomycota (25.9%), Fungi_phy_Incertae_sedis (21.5%) and Kickxellomycota (3.7%) (Figure S6E). At the class level, 37 fungal classes were identified, and the most abundant were Eurotiomycetes (27.3%), Agaricomycetes (23.1%), Fungi_cls_Incertae_sedis (21.5%) and Dothideomycetes (7.1%) (Figure S6F). At the genus level, 228 fungal genera were identified, and *Fungi gen incertae sedis* (21.5%), *Onygenales gen Incertae sedis* (6.0%), *Herpotrichiellaceae gen Incertae sedis* (5.7%) and *Penicillium* (4.1%) were predominant (Figure 3F). Additionally, differences were observed across districts. In the Collonce district, the genus *Flagelloscypha* presented the highest abundance. In the Achamal district, *Mortierella*, *Venturiales gen incertae sedis*, *Linnemania* and *Cladiophialophora* were predominant. In the Huambo district, the most abundant genera were *Cladiophialophora*, *Mucor* and *Ramicandelaber* (Figure 3F).

The co‐occurrence network modelling in the rhizosphere microbiota of cacao and coffee revealed high density and high modularity, except in fungi from cacao (Figures S7 and S8). In cacao, the predominant clustering in bacterial (five modules) and archaeal (two modules) networks resulted from the predominant interaction of eight bacterial taxa and one archaeal taxon (*Methanobrevibacter*), respectively (Figure S7). In coffee, the predominant clustering in bacterial (two modules), archaeal (four modules) and fungal (five modules) networks resulted from the predominant interaction of eight bacterial taxa, three archaeal taxa (*Candidatus Nitrososphaera*, *Candidatus Nitrocosmicus*, *Methanobrevibacter*), and eight fungal taxa (Figure S8).

### 3.3 Analysis of the Diversity of the Microbiota Associated With Cacao and Coffee Rhizospheric Soils

#### 3.3.1 Alpha Diversity

**FIGURE 4:**
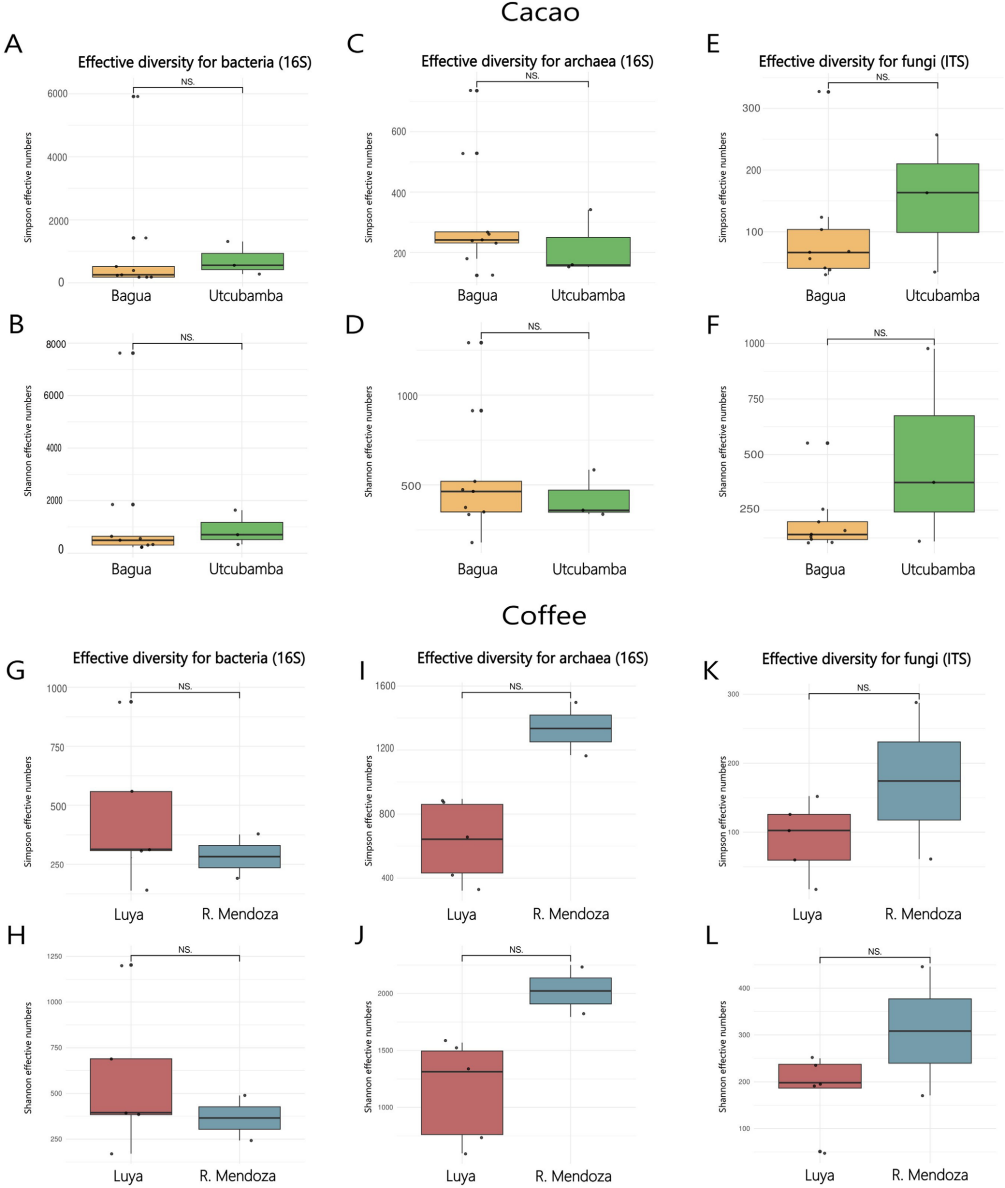
Alpha diversity indices (i.e., Shannon and Simpson) of the microbiota (i.e., bacteria, archaea and fungi) associated with the rhizospheric soils of cacao (A–F) from Bagua and Utcubamba Provinces and coffee (G–L) from Luya and Rodríguez de Mendoza Provinces in the Amazonas region. NS = not significant.

The effective numbers were used for the calculation of alpha diversity indices (i.e., Shannon and Simpson) for the microbiota (i.e., bacteria, archaea and fungi) associated with the cacao and coffee rhizospheric soils (Figure 4).

In the cacao rhizospheric soils (Figure 4A–F), the highest effective diversity was observed for bacteria, whereas the lowest was recorded for fungi. At the provincial level (Utcubamba and Bagua), the effective diversity of bacteria, archaea and fungi was variable. Bagua exhibited slightly greater diversity in bacteria (Shannon = 1360, *p* = 0.75; Simpson = 1031, *p* = 0.79) and archaea (Shannon = 545, *p* = 0.59; Simpson = 312, *p* = 0.45), whereas Utcubamba was more prominent in fungi (Shannon = 486, *p* = 0.094; Simpson = 151, *p* = 0.4). At the district level, the microbial composition was similar (Figure S9). La Peca presented the highest effective diversity for bacteria (Shannon = 3265.83; Simpson = 2530.69), followed by Cajaruro (Shannon = 890.58, Simpson = 715.40) (Figure S9A,D). Aramango presented the highest diversity for archaea (Shannon = 795.09; Simpson = 462.78), followed by Cajaruro (Shannon = 426.95, Simpson = 217.00) (Figure S9B,E). In contrast, Aramango (Shannon = 126.98; Simpson = 66.99) and Copallín (Shannon = 124; Simpson = 55.26) presented the lowest effective diversity for fungi (Figure S9C,F).

In the coffee rhizospheric soils (Figure 4G–L), the highest effective diversity was observed for archaea, followed by bacteria and fungi. In the province of Rodríguez de Mendoza, greater effective diversity was recorded for archaea (Shannon = 2023, *p* = 0.055; Simpson = 1335, *p* = 0.02) and fungi (Shannon = 308, *p* = 0.24; Simpson = 174, *p* = 0.03). In contrast, bacterial effective diversity was greater in Luya (Shannon = 451, *p* = 0.54; Simpson = 451, *p* = 0.51).

Diversity comparisons of the cacao and coffee rhizospheric soil microbiota, based on Hill numbers, revealed that bacteria were the most diverse domain, while archaea were the least diverse (Figure S10). The Hill numbers also indicated that both taxon richness and Shannon diversity were greater in coffee than in cacao for bacteria (*p* < 0.05), archaea and fungi (*p* < 0.05). Conversely, Simpson's dominance was higher in cacao than in coffee for bacteria (*p* < 0.05), archaea and fungi.

#### 3.3.2 Beta Diversity

**FIGURE 5:**
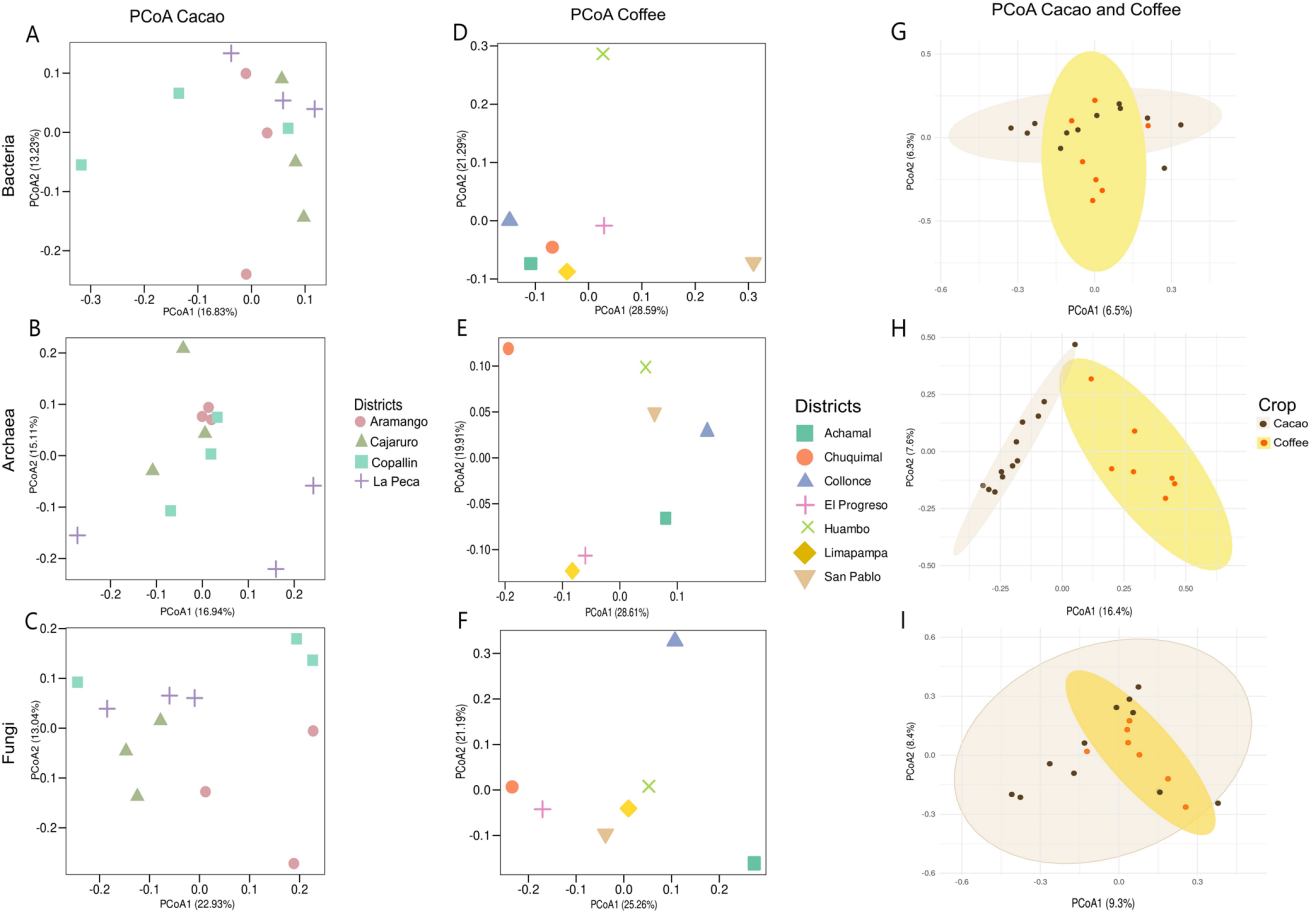
PCoA plot based on the Bray–Curtis distance of the microbiota (i.e., bacteria, archaea and fungi) associated with the rhizospheric soils of cacao farms (A–C), coffee farms (D–F) and both crops (G–I).

Beta diversity analysis was performed using PCoA and DAPC for the microbiota (i.e., bacteria, archaea and fungi) associated with the cacao and coffee rhizospheric soils (Figures 5 and S11).

For the microbiome associated with cacao rhizospheric soils, the PCoA results based on the Bray–Curtis distance explained 30.06%, 32.05% and 35.97% of the total diversity of bacteria, archaea and fungi, respectively (Figure 5A–C). The bacterial and fungal microbiota varied across the districts. In contrast, the archaeal microbiota was similar across all districts, except for samples from La Peca (Figure 5B). To explain the low diversity represented in the PCoA, a DAPC was implemented (Figure S11). The DAPC analysis revealed that the bacterial microbiota from all districts was similar, except for one sample each from Aramango, Cajaruro and Copallin (Figure S11A). The archaeal microbiota clustered into a group containing at least one sample from all districts except Cajaruro (Figure S11B). The fungal microbiota was represented by one group from Cajaruro, another from Aramango, and a third from Copallín and La Peca (Figure S11C).

For the microbiome associated with the coffee rhizospheric soils, the PCoA results based on the Bray–Curtis distance explained 35.31%, 41.89% and 39.69% of the total diversity of bacteria, archaea and fungi, respectively (Figure 5D–F). The archaeal and fungal microbiota showed notable variations across the districts (Figure 5E,F). In contrast, the bacterial microbiota from Achamal, Chuquimal, Collonce, El Progreso and Limapampa clustered into a single group, which was distinct from that from Huambo and San Pablo (Figure 5D). To explain the low diversity in the PCoA, a DAPC was implemented (Figure S11). The DAPC analysis revealed that the bacterial, archaeal and fungal microbiota across all districts did not cluster, suggesting significant variations across the districts (Figure S11D–F).

The analysis of homogeneity of dispersion (Betadisper) revealed significant differences only in the bacterial communities (*F* = 26.16, *p* = 0.001). In contrast, the dispersion of archaeal (*F* = 2.40, *p* = 0.147) and fungal (*F* = 1.06, *p* = 0.297) communities did not differ significantly between crops. Regarding community structure (PERMANOVA, Bray–Curtis), archaeal communities differed significantly between the cacao and coffee rhizospheres (*R* ^2^ = 0.146, F = 2.91, *p* = 0.001). In contrast, neither bacterial (*R* ^2^ = 0.055, *F* = 0.99, *p* = 0.617) nor fungal (*R* ^2^ = 0.044, *F* = 0.78, *p* = 0.994) communities showed significant compositional differences between crops (Figure 5G–I).

#### 3.3.3 Influence of Physicochemical Parameters on the Microbiota Associated With Cacao and Coffee Rhizospheric Soils

The 18 evaluated physicochemical parameters of the cacao and coffee rhizospheric soils are summarised in Table S3. The influence of the physicochemical parameters on the microbiota was evaluated using a PERMANOVA model and db‐RDA. The db‐RDA included the elimination of highly correlated variables (*r* > 0.7) to avoid redundancies and ensure the robustness of the model (Tapaça et al. 2024).

**FIGURE 6:**
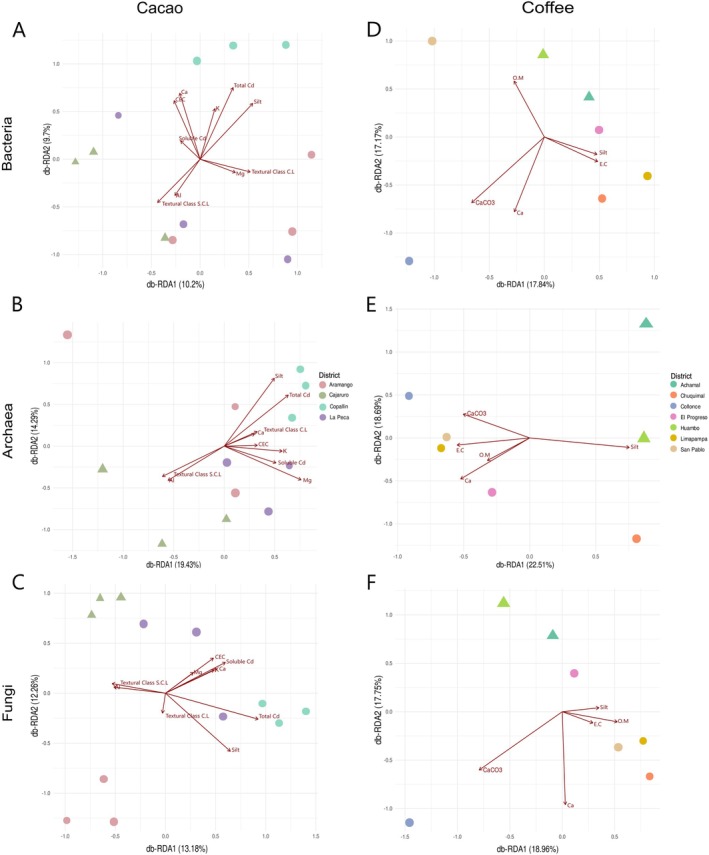
db‐RDA plot representing the variance explained by the influence of physicochemical parameters on the composition of the microbiota of cacao (A–C) and coffee (D–F) rhizospheric soils.

In the cacao rhizospheric soils, the se parameters explained the variance observed in the bacterial (19.9%, Figure 6A), archaeal (33.7%, Figure 6B) and fungal (25.4%, Figure 6C) compositions. The bacterial community composition showed no significant association with any of the physicochemical parameters (*p* value > 0.05). However, the textural class silt influenced the archaeal (*p* value = 0.045) and fungal (*p* value = 0.023) compositions (Table S4). Additionally, the total cadmium concentration influenced the microbial composition in Copallín (Figure 6A–C).

In the coffee rhizospheric soils, these parameters explained the variance observed in the bacterial (35.0%, Figure 6D), archaeal (41.2%, Figure 6E) and fungal (36.7%, Figure 6C) compositions. There was no robust influence of physicochemical parameters on the composition of the microbiota (Table S4). However, the CaCO_3_ concentration and electrical conductivity influenced the microbial composition in Collonce and Limapampa, respectively (Figure 6D–F).

The db‐RDA (CAP) analyses showed that the composition of microbial communities in the rhizospheres is not explained by the crop type (Figure S12). Additionally, under this model, for bacteria, the variables most correlated with community composition were magnesium (Mg; *r* ^2^ = 0.59, *p* = 0.001), exchangeable calcium (C.E.; *r* ^2^ = 0.30, *p* = 0.057) and calcium carbonate (*r* ^2^ = 0.32, *p* = 0.025). In archaea, significant variables included CaCO_3_ (*r* ^2^ = 0.61, *p* = 0.001), organic matter (M.O.; *r* ^2^ = 0.49, *p* = 0.003), silt (*r* ^2^ = 0.52, *p* = 0.005), Mg (*r* ^2^ = 0.54, *p* = 0.002), aluminium (Al; *r* ^2^ = 0.33, *p* = 0.025) and C.E. (*r* ^2^ = 0.35, *p* = 0.03). For fungi, the most influential variables were CaCO_3_ (*r* ^2^ = 0.61, *p* = 0.001), M.O. (*r* ^2^ = 0.55, *p* = 0.003), K (*r* ^2^ = 0.32, *p* = 0.037), silt (*r* ^2^ = 0.36, *p* = 0.041) and C.E. (*r* ^2^ = 0.32, *p* = 0.044).

### 3.4 Functional Profiling of the Microbiota Associated With Cacao and Coffee Rhizospheric Soils

The functional prediction of the microbiota aimed to understand the various biological and ecological functions of the microbiota associated with cacao and coffee rhizospheric soils (Figures S13 and S14).

For the functional profiling of the microbiota of cacao rhizospheric soils, 111 KO codes for bacteria and 105 KO codes for archaea were annotated using PICRUSt2. These codes were associated with five metabolic pathways according to the Kyoto Encyclopedia of Genes and Genomes (KEGG) database (Figure S13A,B). Additionally, 1165 ecological guilds were annotated for fungi according to the FUNGuild tool (Figure S14A,B).

**FIGURE 7:**
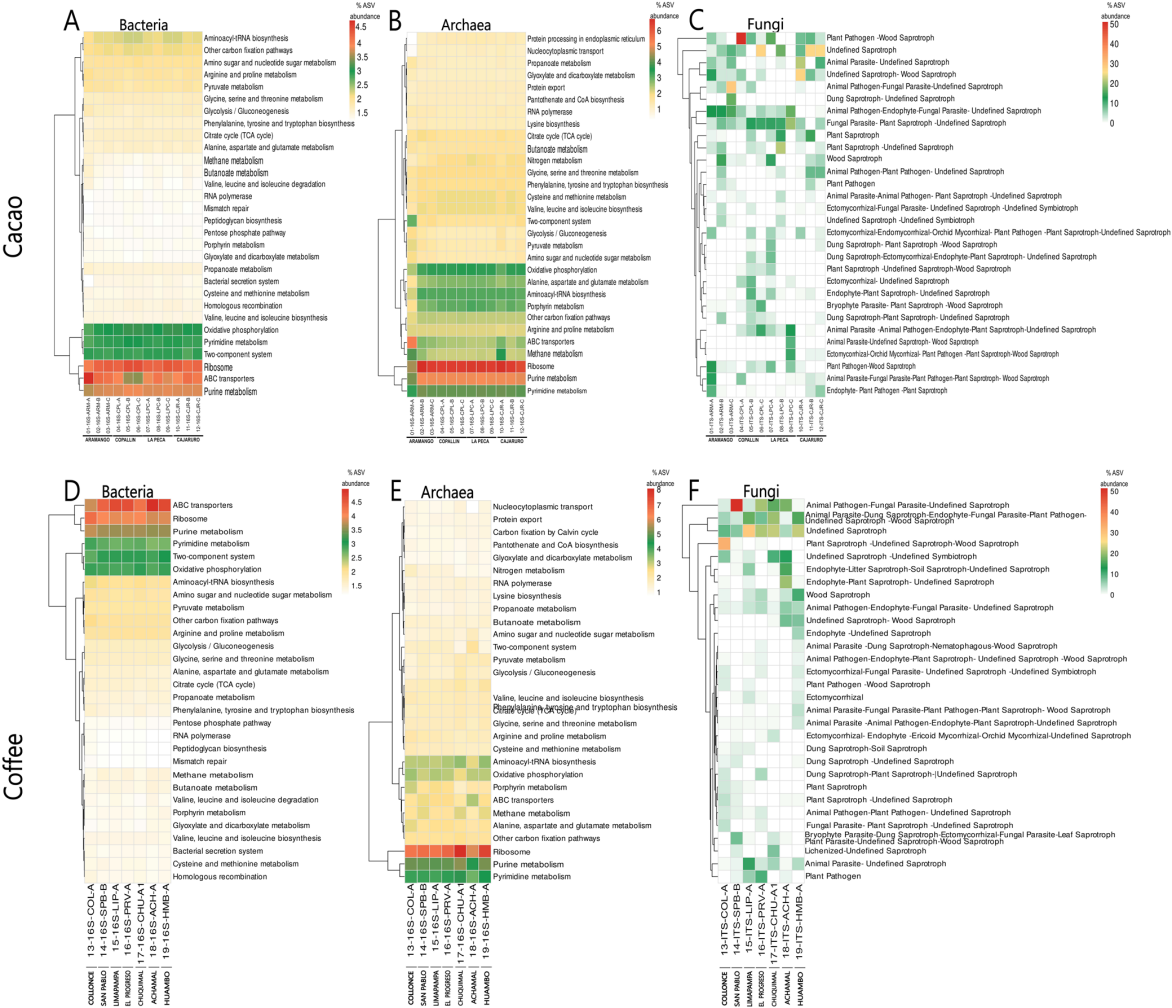
Heatmap displaying the most active predicted pathways for the bacterial (A, D) and archaeal (B, E) microbiota and the ecological guilds for the fungal (C, F) microbiota associated with cacao (A–C) and coffee (D–F) rhizosphere soils in districts from the Amazonas region.

In the cacao rhizosphere soils, the bacterial microbiota across all districts presented high activity in key metabolic pathways, including ribosome, ABC transporter and purine metabolism pathways (red and orange in Figure 7A). Three other pathways—two‐component system, pyrimidine metabolism and oxidative phosphorylation—had moderate activity (green in Figure 7A). Additionally, 10 metabolic pathways related to sugar and amino acid metabolism were considerably activated (yellow in Figure 7A).

The archaeal microbiota was prominent in pathways essential for protein synthesis and basic metabolites, such as ribosome and purine metabolism (red and orange in Figure 7B). Eight other pathways presented moderate activity, including functions related to transporters, phosphorylation, amino acid, porphyrin and pyrimidine metabolism and carbon fixation (green in Figure 7B). Eleven additional pathways displayed low activity and were associated with nitrogen, butanoate, citrate and sugar metabolism (yellow in Figure 7B). Notably, archaeal metabolic activity in some locations of Aramango and Cajaruro differed from each other.

The fungal microbiota exhibited high diversity across the ecological guilds, with distinct variations across the districts (Figure 7C). The most abundant guilds included (i) plant pathogen–wood saprotroph, (ii) undefined saprotroph, (iii) animal parasite–undefined saprotroph, (iv) undefined–wood saprotroph and (v) animal pathogen–fungal parasite–undefined saprotroph. These guilds represent fungi with diverse ecological roles, including pathogens or parasites of plants and animals, as well as key contributors to the decomposition of dead organic matter.

For functional profiling of the coffee rhizosphere soil microbiota, 113 bacterial and 112 archaeal KO codes were annotated using PICRUSt2, corresponding to five metabolic pathways in the KEGG database (Figure S13C,D). Additionally, 744 fungal ecological guilds were annotated using the FUNGuild tool (Figure S14C,D).

**FIGURE 8:**
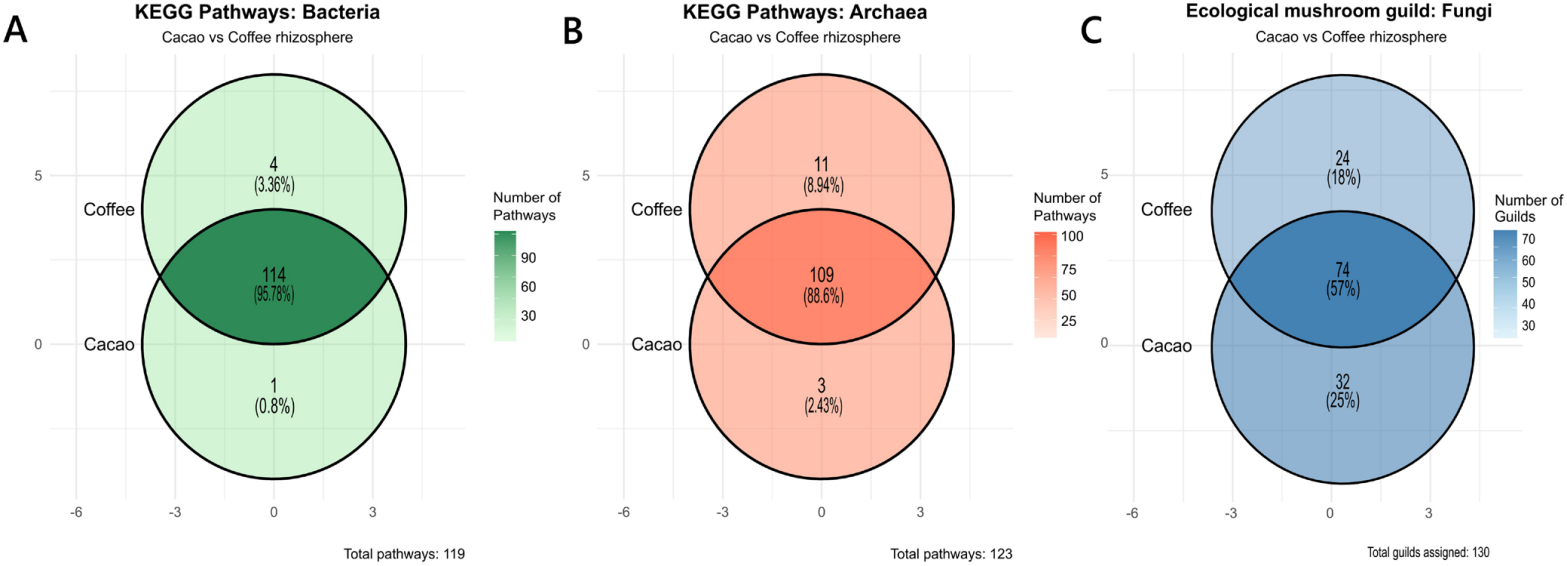
Venn diagram showing core pathways for the bacterial (A) and archaeal (B) microbiota and the core guilds for the fungal (C) microbiota associated with cacao and coffee rhizosphere soils in the Amazonas region.

In the coffee rhizosphere soils, the most active bacterial functional pathways across all districts were ABC transporters, ribosome and purine metabolism (red and orange in Figure 8A). Three other prominent pathways—pyrimidine metabolism, two‐component system and oxidative phosphorylation—had moderate activity (green in Figure 7D). Additionally, five pathways associated with amino acid and sugar metabolism displayed low activation (yellow in Figure 7D).

The archaeal microbiota exhibited high activity specifically in the ribosome pathway (red in Figure 7E). Moderate metabolic activation was detected in four other pathways (i.e., purine metabolism, pyrimidine metabolism, aminoacyl‐tRNA biosynthesis and oxidative phosphorylation; dark green to light green in Figure 7E). Additionally, 12 metabolic pathways associated with amino acid, sugar, methane and nitrogen metabolism showed slight activation (yellow in Figure 7E).

The fungal microbiota exhibited high diversity across the ecological guilds, with distinct variations observed across the coffee districts (Figure 7F). The most abundant guilds included (i) animal pathogen–fungal parasite–undefined saprotroph, (ii) animal parasite–dung saprotroph–endophyte–fungal parasite–plant pathogen–undefined saprotroph–wood saprotroph, (iii) undefined saprotroph and (iv) plant saprotroph–undefined saprotroph–wood saprotroph. These guilds encompass fungi with diverse ecological functions, ranging from animal pathogens and fungal parasites to saprotrophs that decompose dead organic matter. Notably, we also detected endophytic fungi that colonise plant tissues and may provide mutualistic benefits to their host plants.

The comparative analysis of predicted metabolic pathways revealed a high degree of similarity between the functional profiles of microbial communities of cacao and coffee rhizospheric soils. Specifically, 95.8% of bacterial and 88.6% of archaeal pathways, along with 57% of fungal guilds, were identified as a shared functional core between the two crops (Figure 8). In contrast, a small number of pathways were exclusive to each crop: 0.8% of bacterial and 2.43% of archaeal pathways and 25% of fungal guilds were unique to cacao, while 3.5% of bacterial and 8.9% of archaeal pathways and 18% of fungal guilds were unique to coffee. These exclusive pathways suggest functional adaptations specific to each crop.

### 3.5 Enriched Pathways of the Microbiota Associated With Cacao and Coffee Rhizospheric Soils

The analysis identified numerous KEGG metabolic pathways that were statistically significantly enriched in either cacao or coffee rhizospheric soils. The results highlight distinct functional specialisations of the microbiota communities associated with each crop (Figure S15, Tables S5–S7).

Regarding bacteria (Table S5), cacao‐enriched pathways are largely associated with core cellular processes (e.g., ribosome, DNA replication, tRNA biosynthesis), central carbon metabolism (e.g., carbon fixation, TCA cycle) and the biosynthesis of various secondary metabolites. Coffee‐enriched pathways are predominantly linked to stress response and degradation (e.g., metabolism of xenobiotics, drug metabolism, naphthalene degradation) and nutrient cycling (e.g., sulphur and nitrogen metabolism).

In archaea, the results point to a fundamental ecological specialisation. Coffee‐enriched archaea are dominated by autotrophic carbon fixation pathways, whereas cacao‐enriched archaea are strongly specialised in the degradation of man‐made pollutants (xenobiotics) and various nitrogen and carbon metabolic processes (Table S6). This suggests that coffee may favour archaea that function as primary producers, while cacao selects for archaea adept at detoxification and recycling complex compounds.

In fungi, despite several guilds showing large fold changes and nominally significant *p*‐values, none of these differences remained significant after applying a multiple‐testing correction (P_adj > 0.05) (Table S7). The functional potential of the fungal communities, as defined by guild assignment, is not significantly different between the rhizospheric soils of cacao and coffee.

## 4 Discussion

The rhizospheric soil microbiome consists of a wide range of microorganisms that inhabit areas surrounding plant roots and play crucial roles in maintaining ecosystem functions (Tapaça et al. 2024). The interactions between the rhizospheric soil microbiome and plants are key to essential ecosystem processes such as nutrient cycling, soil fertility enhancement and plant disease suppression, all of which contribute to the stability and resilience of ecosystems (Ajala et al. 2022). Therefore, this study unravelled the microbiota diversity associated with rhizospheric soils of the two most important crops in northern Peru (i.e., cacao and coffee) by presenting the first DNA metabarcoding dataset encompassing the three domains of cellular life (i.e., bacteria, archaea and fungi). Furthermore, we examined the influence of physicochemical parameters on the microbiota composition. Additionally, our analysis elucidated the functional profiles of these microbiota communities in the rhizospheric soils of both cacao and coffee crops.

### 4.1 Microbiota Associated With Cacao Rhizospheric Soils

On the basis of the alpha and beta diversity analyses, the microbiota composition of rhizospheric soils in cacao across the districts of Bagua and Utcubamba showed consistent patterns in bacterial communities but exhibited significant variability in archaeal and fungal communities (Figures 2, 3, 4, 5). Accordingly, bacteria, archaea and fungi do not share a common response to soils with similar characteristics on cacao farms. The bacterial microbiota was not influenced by any physicochemical parameters significantly (Figure 6). It has been suggested that (i) the genetic identity of the host plant (Bouffaud et al. 2014), (ii) geographic and climatic homogeneity (Yang, Mai, et al. 2023; Yang, Qiu, et al. 2023) and (iii) uniform agricultural practises (Mo et al. 2024) play crucial roles in shaping the composition of the root‐associated microbiome, particularly in bacterial communities, as bacteria constitute the most prevalent microorganisms, accounting for 70%–90% of the total soil biomass (Wang et al. 2024). The host cacao plants from Bagua and Utcubamba predominantly exhibit a genotype combination of Amelonado, Nacional and Criollo ancestry (Bustamante et al. 2022). This cacao genotype most likely generates a specific microenvironment for bacteria due to root exudates (e.g., sugars, amino acids and organic acids) (Badri and Vivanco 2009). Additionally, the cacao farms in Bagua and Utcubamba present similar environmental conditions (for details, see Vásquez‐García et al. 2022) and agroforestry systems (Goñas et al. 2022). All these factors may act as deterministic factors, creating a balanced bacterial ecosystem with comparable diversity levels across different cacao rhizospheres.

On the other hand, although the fungal and archaeal communities in the rhizospheric soil of cacao exhibit similar effective diversity in Bagua and Utcubamba, they appear to be sensitive to other disturbances, as their compositions differ (Figures 2, 3, 4, 5). Although the percentage of silt influenced the fungal and archaeal microbiota from Copallin (Figure 6), how it shapes these communities remains unclear. This variability in composition suggests that archaeal and fungal communities play distinct ecological roles and are potentially influenced by stochastic processes, as proposed by Neal et al. (2021). For instance, for archaea, both major (Akinola et al. 2023) and minor changes (Gao et al. 2022) in edaphic factors contributed to unpredictable diversity patterns. In fungal communities, while greater diversity is associated with elevated levels of organic matter (Gionchetta et al. 2019), significant selection pressure on fungi is often observed through the use of fungicides, which can increase fungal niche assembly (Neal et al. 2021). Although numerous other factors may influence the diversity and composition of the microbiota associated with the rhizospheric soils of cacao in the Amazonas region, our current data suggest that the bacterial microbiota is shaped by deterministic factors, whereas the archaeal and fungal communities are influenced by stochastic factors.

Despite differences in their response to cacao rhizospheric soils in this study, the prokaryotic and eukaryotic microbiota across all soil types are predominantly composed of a small number of highly abundant microorganisms (Neal et al. 2021). Accordingly, the bacterial (i.e., *Ca. Udaeobacter*, RB41), archaeal (i.e., *Ca. Nitrososphaera*, *Ca. Nitrosocosmicus* and *Methanobrevibacter*) and fungal (i.e., undetermined fungi and *Onygenales*) communities from Bagua and Utcubamba are dominated by specific predominant taxa. The predominant bacteria have been extensively reported in other rhizospheric soils associated with cacao production in Colombia (Cárdenas et al. 2024; Jaramillo‐Mazo et al. 2024). These ubiquitous bacteria (i) significantly influence global soil resistomes and may thrive amid increasing antimicrobial pollution (e.g., *Ca. Udaebacter*) and (ii) play a key role in maintaining soil metabolism, biogeochemical function and cadmium regulation (e.g., *RB41*) (Ai et al. 2018; Wang et al. 2018). Regarding the predominant archaea, this study is the first to report ammonia‐oxidising archaea (AOA) in soils associated with the cacao rhizosphere. *Ca. Nitrososphaera* and *Ca. Nitrosocosmicus* are the two most represented AOA in Bagua and Utcubamba and play significant roles in soil nitrification (Zhalnina et al. 2013), particularly in rhizospheres (Lee et al. 2024). Another predominant archaeon is *Methanobrevibacter*, which is typically reported in the digestive tract of mammals (DasSarma et al. 2010). This archaeon is strictly anaerobic and has an energy metabolism restricted to the formation of methane (Thauer et al. 2008). Regarding the predominant fungi, although Onygenales are decomposers that break down keratin and cellulose (Coleine et al. 2022), fungal taxa cannot be taxonomically classified, and as a result, their influence on the cacao rhizospheric soil remains unclear. The presence of these abundant yet unidentified fungal diversities highlights the unexplored microbiota of the Amazonas region. The absence of a comprehensive reference DNA sequence library, particularly one that incorporates local fungal diversity, likely hinders the annotation of these unknown taxa (Calderon et al. 2024). Despite this limitation, this approach holds significant potential for discovering new fungal species associated with cacao rhizospheric soils.

### 4.2 Microbiota Associated With Coffee Rhizospheric Soils

In the rhizosphere soils of coffee from Luya and Rodríguez de Mendoza Provinces, the diversity analyses revealed results similar to those of the cacao rhizosphere soils. The bacterial communities presented consistent patterns, whereas the archaeal and fungal communities presented greater variability (Figures 2, 3, 4, 5). These findings suggest that microbial communities in rhizosphere coffee soils play distinct ecological roles, with bacteria potentially contributing to stable functional processes, whereas archaea and fungi may respond more dynamically to environmental changes or host‐specific factors (Schöps et al. 2018; Bertola et al. 2021). Additionally, bacterial and fungal diversity was reduced compared with that of archaea, which presented the highest effective diversity in coffee rhizosphere soils (Figure 4). Long‐term coffee monoculture decreased the soil pH, consequently reducing bacterial and fungal richness (Zhao et al. 2018). Accordingly, the acidic soils from the coffee farms in Luya and Rodríguez de Mendoza Provinces, which have over 50 years of coffee‐growing history (Alvarado et al. 2022), negatively impacted bacterial and fungal richness (Table S3).

The composition of the bacterial microbiota in coffee rhizosphere soils is influenced primarily by altitude (Veloso et al. 2023), host species (de Sousa et al. 2022), host variety (Solís Pino et al. 2023) and agroecosystem management (Jurburg et al. 2020). Nevertheless, the bacterial composition in Luya and Rodríguez de Mendoza remained homogeneous across the evaluated varieties (i.e., Caturra and Typica) and altitudes (i.e., 1597–2303 masl). The adoption of mixed agroforestry systems (Haro et al. 2025), along with the predominance of sandy loam soils (Alvarado et al. 2022), in these two provinces appears to be a determining factor in explaining this bacterial homogeneity. On the other hand, the fungal heterogeneity in coffee rhizosphere soils from Luya and Rodríguez de Mendoza is not explained by any of the physicochemical properties evaluated. It has been reported that metabolites secreted by roots strongly influence fungal diversity and composition. For instance, fungi are affected by the sucrose concentration in coffee, which is abundant and triggers microbial colonisation in the rhizosphere (de Sousa et al. 2022). With respect to archaeal heterogeneity, there is limited information available to understand which factors influence the archaeal diversity associated with coffee. Only two archaeal phyla, comprising five genera, were previously reported from coffee roots (one genus; Caldwell et al. 2015) and coffee cherries (four genera; Oliveira et al. 2013). This is not surprising, given that archaea were discovered relatively recently in the history of microbiology (Woese et al. 1978; Duong et al. 2020). Nevertheless, the significant variability of the archaeal species comprising 23 phyla and 129 genera across Luya and Rodríguez de Mendoza was not associated with any physicochemical parameters. These findings suggest that stochastic factors may play a role in shaping archaeal composition, as observed in the cacao rhizosphere.

The microbial communities in coffee rhizospheric soils are largely dominated by a limited number of highly abundant species, similar to those found in rhizospheric cacao soils. For instance, the bacterial (i.e., *Ca. Udaeobacter* and *HSB OF53‐F07*), archaeal (i.e., *Ca. Nitrosotalea*, *Ca. Nitrosocosmicus* and *Ca. Nitrososphaera*) and fungal (i.e., unidentified fungi and Onygenales) communities are primarily composed of dominant taxa. These bacterial and fungal microbiota have been previously reported in coffee soils from Brazil (Andrade et al. 2023), Colombia (Gómez‐Godínez et al. 2024; Ochoa‐Henriquez et al. 2024) and Ecuador (Cruz et al. 2024). The predominant bacterium *Ca. Udaeobacter* has been shown to thrive upon the release of multiple classes of antibiotics (Willms et al. 2020), while the bacterium *HSB OF53‐F07* is prevalent in agricultural soils, although its specific function requires further investigation (Vélez‐Martínez et al. 2024). On the other hand, although one of the predominant fungal taxa in coffee rhizosphere soils belongs to Onygenales, the others remain unclassified, confirming the presence of unexplored microbiota even in the coffee soils of the Amazonas region. The development of a comprehensive and curated DNA reference library at a local or regional scale has already been proposed (Cárdenas et al. 2024), as it could facilitate the identification of these fungi. With respect to archaea, the three AOA identified in coffee rhizosphere soils (i.e., *Ca. Nitrosotalea*, *Ca. Nitrosocosmicus* and *Ca. Nitrososphaera*) are the first to be reported in this crop. These AOA have been reported to play important roles in soil nitrification (Lehtovirta‐Morley et al. 2013).

### 4.3 Standardised Diversity and Core Microbiota Between Cacao and Coffee Rhizospheric Soils

Our findings confirm that the rhizospheric soils of cacao and coffee support highly distinct and complex microbial communities, characterised by a striking predominance of crop‐specific taxa as previously proposed by Lee et al. (2020). This clear differentiation is evidenced by the minimal core microbiome shared across geographical districts for each crop, a pattern especially pronounced in coffee, which lacked a core for archaea and fungi entirely. A similar finding was previously reported for the rhizosphere microbiome of *Coffea arabica* grown in different countries (Bez et al. 2023). Furthermore, the microbial consortia of both crops formed highly modular co‐occurrence networks, suggesting specialised ecological niches; yet coffee exhibited significantly greater taxonomic richness and Shannon diversity. Conversely, the higher Simpson's dominance in cacao implies a community structure driven by a smaller number of more dominant taxa. Collectively, these results demonstrate that the plant host is a powerful filter, selecting for unique and divergent rhizosphere microbiota with distinct structural and diversity patterns (Lee et al. 2020; Compant et al. 2021).

### 4.4 Functional Profiling of the Microbiota Associated With Cacao Rhizospheric Soils

The bacterial functional profiles predicted in the cacao rhizospheric soils were similar across the districts of Bagua and Utcubamba Provinces (Figure 7A). This consistency aligns with the deterministic factor shaping the homogenous bacterial composition. The predominant metabolic pathways, which are highly to moderately activated, are linked to three key processes: protein synthesis (Wilson 2014), nucleic acid synthesis (Brown et al. 2011), and energy metabolism and transport (Davidson et al. 2008). These core metabolic systems appear to function synergistically, simultaneously supporting bacterial growth and adaptive capacity while mediating critical ecological functions in cacao plants, including nutrient acquisition, stress response and the establishment of symbiotic or pathogenic relationships (De Vuyst and Leroy 2020).

The archaeal functional profiles in the cacao rhizosphere soils presented metabolic similarities to those of the bacterial microbiota. However, archaea displayed additional activated pathways, including nitrogen, butanoate, citrate and sugar metabolism (Figure 7B). These supplementary metabolic functions may reflect the disturbance sensitivity of soil influenced by stochastic factors and the presence of active AOA, given their documented metabolic activity in nitrogen cycling (Brown et al. 2011; Zhalnina et al. 2013). While archaeal composition varied between Bagua and Utcubamba, their metabolic profiles showed remarkable consistency, exhibiting only minor variations in community structure (e.g., ARM‐A from Aramango). This similarity leads to the assumption that the archaeal community is driven mostly by functional genes associated with the environment rather than by taxonomic groups. This ecological pattern, first documented in bacterial communities (Calderon et al. 2024), has clearly been demonstrated in archaea inhabiting cacao rhizosphere ecosystems.

Soil fungal communities are typically classified into three major functional guilds on the basis of trophic mode (i.e., saprotrophs, pathotrophs and symbiotrophs) (Frac et al. 2018; Lin et al. 2022). However, diverse ecological roles were observed for saprotrophic fungi in the cacao rhizosphere soils across all the districts (Figure 7C). These specific guild distributions were likely shaped by stochastic factors not identified in this study. The predominance of saprotrophs may be linked to the abundant organic matter on cacao farms resulting from extensive soil management under agroforestry systems (Goñas et al. 2022). Previous studies have suggested that such soil management practises significantly alter fungal communities in cacao soils (Arévalo‐Gardini et al. 2020). This observation may explain the lack of consistent guild patterns across cacao rhizospheric soils in Bagua and Utcubamba. Notably, the relatively low abundance of both pathotrophic and symbiotrophic fungi underscores the delicate balance between these functional groups in cacao ecosystems. Importantly, proper management practises can shift this balance towards more beneficial, symbiotroph‐dominated communities (Arévalo‐Gardini et al. 2020; Prada‐Salcedo et al. 2021).

### 4.5 Functional Prediction of the Microbiota Associated With Coffee Rhizospheric Soils

The bacterial functional profiles in the coffee rhizospheric soils were similar across the districts of Luya and Rodríguez de Mendoza (Figure 7D). As observed on cacao farms, the homogeneous bacterial composition in coffee rhizospheric soils led to a correspondingly uniform functional profile. This profile is associated primarily with key metabolic pathways, including nucleic acid metabolism, transport, protein and biosynthesis processes previously reported in coffee fermentation (Perez et al. 2023; Calderon et al. 2024) and soil characterisation (Duong et al. 2020). This conserved metabolic behaviour persists across various stages of coffee production, highlighting the critical role of bacteria in these ecosystems.

The archaeal functional profiles of the coffee rhizosphere soils revealed metabolic pathways analogous to those associated with the bacterial microbiota. Nevertheless, additional metabolic pathways such as nitrogen and methane metabolism were activated uniformly across the districts by different and active AOA (Figure 7E). This functional homology supports an environmental gene‐driven model of archaeal community assembly, where metabolic potential supersedes taxonomic identity as the primary organisational principle (Calderon et al. 2024).

The predominant functional guilds of fungal communities in the coffee rhizosphere soils of Luya and Rodríguez de Mendoza were primarily saprotrophic (Figure 7F). This ecological role likely stems from the abundant organic matter derived from the mixed agroforestry systems on these coffee farms (Alvarado et al. 2022; Haro et al. 2025). Saprotrophic guilds play a critical role in maintaining soil health and facilitating nutrient cycling (Hannula and Träger 2020). Conversely, the limited representation of pathotrophic and symbiotrophic guilds may reflect the inhibitory effects of reduced soil pH, which constrains mutualistic interactions and significantly alters fungal community composition (Zhang et al. 2016).

### 4.6 Functional Redundancy in Cacao and Coffee Rhizospheres

The high degree of functional similarity between the cacao and coffee rhizospheres confirms that the functional profile of the rhizosphere microbiome is shaped more strongly by the conserved habitat filters of the rhizosphere than by host‐specific identity (Jia and Whalen 2020). This conserved functional profile is essential for survival in microbial communities (Figures 7 and 8). This functional convergence is maintained through widespread functional redundancy across microbial taxa, wherein a core set of essential metabolic functions is preserved irrespective of phylogenetic composition (Rosenfeld 2002). Conversely, the small fraction of unique metabolic pathways suggests a degree of functional specialisation and niche partitioning, potentially driven by subtle differences in host root exudates, representing the locus for highly specific plant‐microbiome associations (Badri and Vivanco 2009; de Sousa et al. 2022).

Regarding the enrichment pathways, our findings demonstrate a striking functional divergence in prokaryotes (bacteria and archaea), contrasted by a remarkable functional stability in fungi between the rhizospheres of cacao and coffee. The microbiome responds to the plant host in a tiered manner (Lajoie et al. 2020; Song et al. 2021). Prokaryotes (bacteria and archaea) represent the “specialists,” rapidly adapting their metabolic networks to the specific chemical milieu of each rhizosphere. For instance, the systems employ different carbon acquisition strategies. The coffee rhizosphere appears to rely more on archaeal autotrophy (Calvin cycle), while the cacao rhizosphere utilises bacterial autotrophy (other pathways) alongside heterotrophic degradation of complex organic matter. This highlights how different crops can alter the very foundations of the soil food web (Liu et al. 2023). Conversely, fungi represent the “generalists,” providing a stable, redundant functional backbone that is less sensitive to changes between the two crops. This analysis demonstrates that a holistic, multi‐kingdom perspective is essential to understanding rhizosphere ecology. The responses of one domain to another cannot be extrapolated (Leff et al. 2015). The clear functional partitioning between cacao and coffee seen in prokaryotes is entirely absent in fungi, revealing a fundamental dichotomy in how these microbial kingdoms assemble and function. Future work should focus on linking these distinct functional profiles to specific agroecosystem outcomes, such as plant health, carbon sequestration and resilience to agricultural chemicals, with a particular emphasis on the newly highlighted role of archaea in rhizosphere biogeochemistry.

## 5 Conclusion

The metagenomic characterisation of rhizosphere‐associated microbiota in the cacao and coffee systems from the Amazonas region revealed deterministic assembly of bacterial communities, in contrast with the stochastic structuring of archaeal and fungal assemblages. Further research should focus on elucidating the environmental drivers underlying these distinct community assembly patterns.

Additionally, the bacterial, archaeal and fungal microbiota across all rhizospheric soils of cacao and coffee were predominantly composed of a small number of highly abundant microorganisms. While archaeal and bacterial species demonstrate relatively high taxonomic resolution, the current lack of a comprehensive reference DNA sequence library, particularly one encompassing local fungal diversity, continues to challenge the accurate annotation of autochthonous microorganisms. Additionally, cacao and coffee cultivate highly specific bacterial, archaeal and fungal communities, with minimal shared core microbiomes. However, the assembly rules and functional outcomes for these communities differ fundamentally. Bacterial communities in both crops exhibited remarkable compositional and functional homogeneity, shaped by deterministic factors like host genotype and management practises. In contrast, archaeal and fungal compositions were variable and influenced by stochastic factors, yet their functional profiles remained consistent within each crop, suggesting that environmental gene‐driven selection, rather than taxonomic identity, governs their metabolic output.

This multi‐kingdom perspective reveals that functional convergence in core rhizosphere processes is maintained through widespread taxonomic redundancy. Nevertheless, a small fraction of unique, enriched pathways underscores the potential for host‐specific fine‐tuning of the microbiome. These findings highlight the need for holistic management strategies that consider the distinct assembly rules and roles of each microbial domain. Additionally, these findings highlight the substantial potential for discovering novel microbial taxa and previously unrecognised functional roles within cacao and coffee rhizosphere ecosystems. Furthermore, these findings underscore the biotechnological potential of indigenous soil microorganisms for enhancing productivity and sustainability across cacao and coffee cultivation systems. Future research should leverage these insights to link these specific microbial functional profiles to tangible agroecosystem outcomes, such as crop health, soil carbon storage and resilience to agricultural stresses, with a particular focus on the newly elucidated and critical role of archaea in rhizosphere biogeochemistry.

## Author Contributions

**Jois V. Carrion:** data curation, formal analysis, investigation, writing – original draft, writing – review and editing, validation, visualization. **Maricela Chavez:** investigation, validation, writing – review and editing. **Yadhira M. Olano:** investigation, validation, writing – review and editing. **Martha S. Calderon:** conceptualization, funding acquisition, supervision, methodology, writing – review and editing, writing – original draft, resources. **Danilo E. Bustamante:** conceptualization, funding acquisition, methodology, supervision, writing – review and editing, writing – original draft, project administration, resources.

## Funding

This research was fully funded by the Peruvian National Council for Science, Technology, and Technological Innovation (CONCYTEC) through its PROCIENCIA program, under the call for ‘Special Projects—EULAC—Research Infrastructures’ (N°PE501079652‐2022, MiCroResi). This research was also funded by CONCYTEC under the project Metacafé 2.0 (N°PE501081184‐2022‐PROCIENCIA).

## Conflicts of Interest

The authors declare no conflicts of interest.

## Supporting information


**Table S1:** Sampling information for the cacao and coffee rhizospheric soil samples obtained from Amazonas region.
**Table S2:** Read quality processing results for the microbiota associated with rhizospheric soil samples from cacao and coffee farms.
**Table S3:** Physicochemical parameters measured at each sampling point from the rhizospheric soils of the cacao and coffee farms. C = clay; C.L. = clay loam; L = loam; S = sand; S.C.L. = sandy clay loam; S.L. = sandy loam.
**Table S4:** PERMANOVA of the microbiota composition and physicochemical parameters of rhizospheric soils from the cacao and coffee farms. Statistically significant differences are indicated by asterisks (*p* < 0.05).
**Table S5:** Differential abundance analysis of KEGG metabolic pathways for bacteria in the rhizospheric soils of cacao and coffee from the Amazonas region. The log_2_ Fold Change represents the change in abundance between groups; lfcSE is the associated standard error; and P_adj is the adjusted *p*‐value for multiple comparisons.
**Table S6:** Differential abundance analysis of KEGG metabolic pathways for archaea in the rhizospheric soils of cacao and coffee from the Amazonas region. The log_2_ Fold Change represents the change in abundance between groups; lfcSE is the associated standard error; and P_adj is the adjusted *p*‐value for multiple comparisons.
**Table S7:** Differential abundance analysis of guilds for fungi in the rhizospheric soils of cacao and coffee from the Amazonas region. The log_2_ Fold Change represents the change in abundance between groups; lfcSE is the associated standard error; and P_adj is the adjusted *p*‐value for multiple comparisons.

**Figure S1:** Workflow of rhizospheric soil collection from coffee (left) and cacao (right) farms in the Amazonas region. Created with BioRender.com.
**Figure S2:** Workflow of physicochemical (A) and molecular (B) analyses of rhizospheric soil samples from cacao and coffee farms in the Amazonas region. Created with BioRender.com.
**Figure S3:** Quality and Phred score for each of the 250 base pairs (bp) of the unprocessed sequences from the 16S rRNA (bacteria), 16S rRNA (archaea) and ITS (fungi) regions. High‐quality values for forward and reverse reads are represented in green, while low‐quality sequences are highlighted in red.
**Figure S4:** Phred quality scores for each of the 250 base pairs (bp) in the 16S rRNA (bacteria), 16S rRNA (archaea) and ITS (fungi) regions after filtering. High‐quality values for forward (A) and reverse (B) reads are displayed in green, while sequences with low quality are highlighted in red.
**Figure S5:** Taxonomic composition of the microbiota (i.e., bacteria, archaea and fungi) associated with cacao rhizospheric soils from Bagua and Utcubamba Provinces at the phylum (A, C, E) and class (B, D, F) levels.
**Figure S6:** Taxonomic composition of the microbiota (i.e., bacteria, archaea and fungi) associated with coffee rhizospheric soils from Luya and Rodríguez de Mendoza Provinces at the phylum (A, C, E) and class (B, D, F) levels.
**Figure S7:** Co‐occurrence network of microbiota associated with the cacao rhizospheric soils in the Amazonas, using a filter threshold of 0.001 and a correlation coefficient of 0.6.
**Figure S8:** Co‐occurrence network of microbiota associated with the coffee rhizospheric soils in the Amazonas region, using a filter threshold of 0.0005 and a correlation coefficient of 0.5.
**Figure S9:** Alpha diversity indices of the microbiota (i.e., bacteria, archaea and fungi), evaluated using the Shannon (A–C) and Simpson indices (D–F), associated with the rhizospheric soils of cacao plantations across the different districts of the Amazonas region.
**Figure S10:** Diversity comparisons of the cacao and coffee rhizospheric soil microbiota [i.e., bacteria (A), archaea (B) and fungi (C)] based on Hill numbers for taxon richness (D0), Shannon diversity (D1) and Simpson's dominance (D2).
**Figure S11:** DAPC with two linear discriminants (LD1 and LD2) of the microbiota (i.e., bacteria, archaea and fungi) associated with the rhizospheric soils of cacao (A‐C) and coffee (D‐F) farms from the Amazonas region.
**Figure S12:** db‐RDA‐CAP plot representing the variance explained by the influence of physicochemical parameters on the composition of the microbiota for both crops (G‐I).
**Figure S13:** Predicted pathways for biological functions and subfunctions of bacterial and archaeal microbiota associated with rhizospheric soils of cacao (A, B) and coffee (C, D) farms from the Amazonas region.
**Figure S14:** Bar chart showing assigned and unassigned ecological guilds, along with confidence rankings, for fungi associated with cacao (A, B) and coffee (C, D) rhizosphere soils in the Amazonas region.
**Figure S15:** Enriched pathways of microbiota associated with cacao and coffee rhizosphere soils in the Amazonas region.

## Data Availability

The datasets presented in this study can be found in online repositories. Sequence data that support the findings of this study have been deposited in the National Center for Biotechnology Information database under BioProject numbers PRJNA1085872, PRJNA1085873, PRJNA1085877 and PRJNA1085878. Additional information can be found in the Supporting Information.
