# Supplementary material for: Functional Specialisation and Diversity Shape the Rhizosphere Microbiota of Cacao and Coffee in the Amazonas Region, Peru
Source: Environ Microbiol Rep. 2025 Dec 9;17(6):e70259. doi: 10.1111/1758-2229.70259 (PMC12688408; doi:10.1111/1758-2229.70259)
Supplement: Supplementary file 1 — Table S1: Sampling information for the cacao and coffee rhizospheric soil samples obtained from Amazonas region. Table S2: Read quality processing results for the microbiota associated with rhizospheric soil samples from cacao and coffee farms. Table S3: Physicochemical parameters measured at each sampling point from the rhizospheric soils of the cacao and coffee farms. C = clay; C.L. = clay loam; L = loam; S = sand; S.C.L. = sandy clay loam; S.L. = sandy loam. Table S4: PERMANOVA of the microbiota composition and physicochemical parameters of rhizospheric soils from the cacao and coffee farms. Statistically significant differences are indicated by asterisks (p < 0.05). Table S5: Differential abundance analysis of KEGG metabolic pathways for bacteria in the rhizospheric soils of cacao and coffee from the Amazonas region. The log2 Fold Change represents the change in abundance between groups; lfcSE is the associated standard error; and P_adj is the adjusted p‐value for multiple comparisons. Table S6: Differential abundance analysis of KEGG metabolic pathways for archaea in the rhizospheric soils of cacao and coffee from the Amazonas region. The log2 Fold Change represents the change in abundance between groups; lfcSE is the associated standard error; and P_adj is the adjusted p‐value for multiple comparisons. Table S7: Differential abundance analysis of guilds for fungi in the rhizospheric soils of cacao and coffee from the Amazonas region. The log2 Fold Change represents the change in abundance between groups; lfcSE is the associated standard error; and P_adj is the adjusted p‐value for multiple comparisons. [file EMI4-17-e70259-s002.docx]

**Supplemental table**

**Table S1.** Sampling information for the cacao and coffee rhizospheric soil samples obtained from Amazonas region.

| **Soil crops** | **Microbial group** | **Bioproject accession** | **SRR accession** | **Codes** | **Collection Date** | **Coordinates** | | **Location** |
| --- | --- | --- | --- | --- | --- | --- | --- | --- |
|  |  |  |  |  |  | **East** | **South** |  |
| **Cacao samples** | **Bacteria** | PRJNA1085872 | SRR28278099 | ARM-A | 21/06/2023 | -5.437249 | -78.442347 | Aramango, Bagua |
|  |  | PRJNA1085872 | SRR28278098 | ARM-B | 21/06/2023 | -5.437652 | -78.442522 | Aramango, Bagua |
|  |  | PRJNA1085872 | SRR28278088 | ARM-C | 21/06/2023 | -5.433416 | -78.441538 | Aramango, Bagua |
|  |  | PRJNA1085872 | SRR28278087 | CPL-A | 21/06/2023 | -5.661978 | -78.380337 | Copallin, Bagua |
|  |  | PRJNA1085872 | SRR28278086 | CPL-B | 21/06/2023 | -5.662414 | -78.38135 | Copallin, Bagua |
|  |  | PRJNA1085872 | SRR28278085 | CPL-C | 21/06/2023 | -5.664838 | -78.379234 | Copallin, Bagua |
|  |  | PRJNA1085872 | SRR28278084 | LPC-A | 21/06/2023 | -5.619701 | -78.424009 | La Peca, Bagua |
|  |  | PRJNA1085872 | SRR28278083 | LPC-B | 21/06/2023 | -5.62162 | -78.418581 | La Peca, Bagua |
|  |  | PRJNA1085872 | SRR28278082 | LPC-C | 21/06/2023 | -5.625778 | -78.397333 | La Peca, Bagua |
|  |  | PRJNA1085873 | SRR28278081 | CJR-A | 21/06/2023 | -5.742306 | -78.295304 | Cajaruro, Utcubamba |
|  |  | PRJNA1085872 | SRR28278097 | CJR-B | 21/06/2023 | -5.753404 | -78.291556 | Cajaruro, Utcubamba |
|  |  | PRJNA1085872 | SRR28278096 | CJR-C | 21/06/2023 | -5.746883 | -78.286992 | Cajaruro, Utcubamba |
|  | **Fungi** | PRJNA1085878 | SRR28278207 | ARM-A | 21/06/2023 | -5.437249 | -78.442347 | Aramango, Bagua |
|  |  | PRJNA1085878 | SRR28278206 | ARM-B | 21/06/2023 | -5.437652 | -78.442522 | Aramango, Bagua |
|  |  | PRJNA1085878 | SRR28278196 | ARM-C | 21/06/2023 | -5.433416 | -78.441538 | Aramango, Bagua |
|  |  | PRJNA1085878 | SRR28278195 | CPL-A | 21/06/2023 | -5.661978 | -78.380337 | Copallin, Bagua |
|  |  | PRJNA1085878 | SRR28278194 | CPL-B | 21/06/2023 | -5.662414 | -78.38135 | Copallin, Bagua |
|  |  | PRJNA1085878 | SRR28278193 | CPL-C | 21/06/2023 | -5.664838 | -78.379234 | Copallin, Bagua |
|  |  | PRJNA1085878 | SRR28278192 | LPC-A | 21/06/2023 | -5.619701 | -78.424009 | La Peca, Bagua |
|  |  | PRJNA1085878 | SRR28278191 | LPC-B | 21/06/2023 | -5.62162 | -78.418581 | La Peca, Bagua |
|  |  | PRJNA1085878 | SRR28278190 | LPC-C | 21/06/2023 | -5.625778 | -78.397333 | La Peca, Bagua |
|  |  | PRJNA1085878 | SRR28278189 | CJR-A | 21/06/2023 | -5.742306 | -78.295304 | Cajaruro, Utcubamba |
|  |  | PRJNA1085878 | SRR28278205 | CJR-B | 21/06/2023 | -5.753404 | -78.291556 | Cajaruro, Utcubamba |
|  |  | PRJNA1085878 | SRR28278204 | CJR-C | 21/06/2023 | -5.746883 | -78.286992 | Cajaruro, Utcubamba |
|  | **Archaea** | PRJNA1085877 | SRR28292700 | ARM-A | 21/06/2023 | -5.437249 | -78.442347 | Aramango, Bagua |
|  |  | PRJNA1085877 | SRR28292699 | ARM-B | 21/06/2023 | -5.437652 | -78.442522 | Aramango, Bagua |
|  |  | PRJNA1085877 | SRR28292708 | ARM-C | 21/06/2023 | -5.433416 | -78.441538 | Aramango, Bagua |
|  |  | PRJNA1085877 | SRR28292707 | CPL-A | 21/06/2023 | -5.661978 | -78.380337 | Copallin, Bagua |
|  |  | PRJNA1085877 | SRR28292706 | CPL-B | 21/06/2023 | -5.662414 | -78.38135 | Copallin, Bagua |
|  |  | PRJNA1085877 | SRR28292705 | CPL-C | 21/06/2023 | -5.664838 | -78.379234 | Copallin, Bagua |
|  |  | PRJNA1085877 | SRR28292704 | LPC-A | 21/06/2023 | -5.619701 | -78.424009 | La Peca, Bagua |
|  |  | PRJNA1085877 | SRR28292703 | LPC-B | 21/06/2023 | -5.62162 | -78.418581 | La Peca, Bagua |
|  |  | PRJNA1085877 | SRR28292702 | LPC-C | 21/06/2023 | -5.625778 | -78.397333 | La Peca, Bagua |
|  |  | PRJNA1085877 | SRR28292701 | CJR-A | 21/06/2023 | -5.742306 | -78.295304 | Cajaruro, Utcubamba |
|  |  | PRJNA1085877 | SRR28292698 | CJR-B | 21/06/2023 | -5.753404 | -78.291556 | Cajaruro, Utcubamba |
|  |  | PRJNA1085877 | SRR28292697 | CJR-C | 21/06/2023 | -5.746883 | -78.286992 | Cajaruro, Utcubamba |
| **Coffee samples** | **Bacteria** | PRJNA1085872 | SRR28278095 | COL-A | 11/07/2023 | -6.274361 | -78.172223 | Collonce, Ocumal, Luya |
|  |  | PRJNA1085872 | SRR28278094 | SPB-B | 11/07/2023 | -6.281985 | -78.21085 | San Pablo, Lonya Chico, Luya |
|  |  | PRJNA1085872 | SRR28278093 | LIP-A | 11/07/2023 | -6.265982 | -78.210046 | Limapampa, Ocumal, Luya |
|  |  | PRJNA1085872 | SRR28278092 | PRV-A | 11/07/2023 | -6.274823 | -78.16968 | El Progreso, Ocumla, Luya |
|  |  | PRJNA1085872 | SRR28278091 | CHU-A1 | 11/07/2023 | -6.241703 | -78.154925 | Chuquimal, Ocumal, Luya |
|  |  | PRJNA1085872 | SRR28278090 | ACH-A | 24/07/2023 | -6.541572 | -77.410553 | Achamal, Chirimoto, Rodríguez de Mendoza |
|  |  | PRJNA1085872 | SRR28278089 | HMB-A | 24/07/2023 | -6.541805 | -77.41089 | Huambo, Rodríguez de Mendoza |
|  | **Fungi** | PRJNA1085878 | SRR28278203 | COL-A | 11/07/2023 | -6.274361 | -78.172223 | Collonce, Ocumal, Luya |
|  |  | PRJNA1085878 | SRR28278202 | SPB-B | 11/07/2023 | -6.281985 | -78.21085 | San Pablo, Lonya Chico, Luya |
|  |  | PRJNA1085878 | SRR28278201 | LIP-A | 11/07/2023 | -6.265982 | -78.210046 | Limapampa, Ocumal, Luya |
|  |  | PRJNA1085878 | SRR28278200 | PRV-A | 11/07/2023 | -6.274823 | -78.16968 | El Progreso, Ocumla, Luya |
|  |  | PRJNA1085878 | SRR28278199 | CHU-A1 | 11/07/2023 | -6.241703 | -78.154925 | Chuquimal, Ocumal, Luya |
|  |  | PRJNA1085878 | SRR28278198 | ACH-A | 24/07/2023 | -6.541572 | -77.410553 | Achamal, Chirimoto, Rodríguez de Mendoza |
|  |  | PRJNA1085878 | SRR28278197 | HMB-A | 24/07/2023 | -6.541805 | -77.41089 | Huambo, Rodríguez de Mendoza |
|  | **Archaea** | PRJNA1085877 | SRR28292715 | COL-A | 11/07/2023 | -6.274361 | -78.172223 | Collonce, Ocumal, Luya |
|  |  | PRJNA1085877 | SRR28292714 | SPB-B | 11/07/2023 | -6.281985 | -78.21085 | San Pablo, Lonya Chico, Luya |
|  |  | PRJNA1085877 | SRR28292713 | LIP-A | 11/07/2023 | -6.265982 | -78.210046 | Limapampa, Ocumal, Luya |
|  |  | PRJNA1085877 | SRR28292712 | PRV-A | 11/07/2023 | -6.274823 | -78.16968 | El Progreso, Ocumla, Luya |
|  |  | PRJNA1085877 | SRR28292711 | CHU-A1 | 11/07/2023 | -6.241703 | -78.154925 | Chuquimal, Ocumal, Luya |
|  |  | PRJNA1085877 | SRR28292710 | ACH-A | 24/07/2023 | -6.541572 | -77.410553 | Achamal, Chirimoto, Rodríguez de Mendoza |
|  |  | PRJNA1085877 | SRR28292709 | HMB-A | 24/07/2023 | -6.541805 | -77.41089 | Huambo, Rodríguez de Mendoza |

**Table S2.** Read quality processing results for the microbiota associated with rhizospheric soil samples from cacao and coffee farms.

| **Crops** | **Marker** | **Accession/ GenBankID** | **BioSample** | **Sequencing code** | **Input** | **Filtered** | **DadaF** | **DadaR** | **Merged** | **Nonchim** |
| --- | --- | --- | --- | --- | --- | --- | --- | --- | --- | --- |
| Cacao | 16SrRNA Bacteria | SRR28278099 | SAMN40349095 | 16S-01-ARM-A | 90854 | 90854 | 77312 | 74848 | 47103 | 37756 |
|  |  | SRR28278098 | SAMN40349096 | 16S-02-ARM-B | 42499 | 42499 | 37459 | 36523 | 20092 | 14012 |
|  |  | SRR28278088 | SAMN40349097 | 16S-03-ARM-C | 51237 | 51237 | 46051 | 44402 | 26690 | 21231 |
|  |  | SRR28278087 | SAMN40349098 | 16S-04-CPL-A | 106571 | 106571 | 98509 | 95911 | 62831 | 48487 |
|  |  | SRR28278086 | SAMN40349099 | 16S-05-CPL-B | 38169 | 38169 | 33958 | 32323 | 18440 | 15204 |
|  |  | SRR28278085 | SAMN40349100 | 16S-06-CPL-C | 70218 | 70218 | 64115 | 62842 | 39004 | 30515 |
|  |  | SRR28278084 | SAMN40349101 | 16S-07-LPC-A | 63554 | 63554 | 56513 | 55448 | 30639 | 23058 |
|  |  | SRR28278083 | SAMN40349102 | 16S-08-LPC-B | 1518271 | 1518271 | 1469663 | 1466389 | 1172225 | 470687 |
|  |  | SRR28278082 | SAMN40349103 | 16S-09-LPC-C | 244897 | 244897 | 229109 | 227763 | 164643 | 99585 |
|  |  | SRR28278081 | SAMN40349104 | 16S-10-CJR-A | 38282 | 38282 | 34471 | 33362 | 23299 | 17921 |
|  |  | SRR28278097 | SAMN40349105 | 16S-11-CJR-B | 98058 | 98058 | 90678 | 89847 | 63845 | 41372 |
|  |  | SRR28278096 | SAMN40349106 | 16S-12-CJR-C | 185645 | 185645 | 175434 | 173880 | 133938 | 78797 |
|  | ITS Fungi | SRR28278207 | SAMN40349156 | ITS-01-ARM-A | 129183 | 27669 | 26110 | 25446 | 21484 | 18654 |
|  |  | SRR28278206 | SAMN40349157 | ITS-02-ARM-B | 115782 | 42685 | 38100 | 37119 | 31234 | 27254 |
|  |  | SRR28278196 | SAMN40349158 | ITS-03-ARM-C | 65699 | 24969 | 21219 | 20623 | 17912 | 14860 |
|  |  | SRR28278195 | SAMN40349159 | ITS-04-CPL-A | 69429 | 25071 | 17865 | 16879 | 13531 | 12159 |
|  |  | SRR28278194 | SAMN40349160 | ITS-05-CPL-B | 112770 | 38234 | 30143 | 28119 | 20984 | 19369 |
|  |  | SRR28278193 | SAMN40349161 | ITS-06-CPL-C | 119820 | 35942 | 30323 | 28572 | 21555 | 19205 |
|  |  | SRR28278192 | SAMN40349162 | ITS-07-LPC-A | 154956 | 55529 | 49318 | 46152 | 35668 | 33208 |
|  |  | SRR28278191 | SAMN40349163 | ITS-08-LPC-B | 65729 | 23914 | 20534 | 19343 | 15131 | 14067 |
|  |  | SRR28278190 | SAMN40349164 | ITS-09-LPC-C1 | 281057 | 137028 | 115349 | 112424 | 88010 | 87703 |
|  |  | SRR28278189 | SAMN40349165 | ITS-10-CJR-A | 247795 | 104434 | 81281 | 78843 | 46508 | 45786 |
|  |  | SRR28278205 | SAMN40349166 | ITS-11-CJR-B | 200994 | 75803 | 54873 | 52562 | 19512 | 19506 |
|  |  | SRR28278204 | SAMN40349167 | ITS-12-CJR-C | 46809 | 17780 | 13197 | 11640 | 9296 | 8866 |
|  | 16SrRNA Archaea | SRR28292700 | SAMN40349133 | 18S-01-ARM-A | 53971 | 27694 | 23150 | 21439 | 14656 | 8778 |
|  |  | SRR28292699 | SAMN40349134 | 18S-02-ARM-B | 345888 | 203015 | 181017 | 174649 | 144358 | 55783 |
|  |  | SRR28292708 | SAMN40349135 | 18S-03-ARM-C | 218088 | 131803 | 107349 | 102180 | 80781 | 36532 |
|  |  | SRR28292707 | SAMN40349136 | 18S-04-CPL-A | 177897 | 101297 | 74323 | 69406 | 57047 | 23342 |
|  |  | SRR28292706 | SAMN40349137 | 18S-05-CPL-B | 256166 | 145227 | 102882 | 98629 | 78827 | 29364 |
|  |  | SRR28292705 | SAMN40349138 | 18S-06-CPL-C | 270480 | 155226 | 121451 | 114640 | 91949 | 36284 |
|  |  | SRR28292704 | SAMN40349139 | 18S-07-LPC-A | 180220 | 112867 | 109525 | 109087 | 98716 | 29468 |
|  |  | SRR28292703 | SAMN40349140 | 18S-08-LPC-B | 124561 | 85784 | 82405 | 81510 | 74188 | 25488 |
|  |  | SRR28292702 | SAMN40349141 | 18S-09-LPC-C | 215868 | 127374 | 103481 | 98764 | 81731 | 30255 |
|  |  | SRR28292701 | SAMN40349142 | 18S-10-CJR-A | 128962 | 74263 | 58561 | 54361 | 43137 | 20496 |
|  |  | SRR28292698 | SAMN40349143 | 18S-11-CJR-B | 253819 | 154302 | 128847 | 122764 | 104731 | 41445 |
|  |  | SRR28292697 | SAMN40349144 | 18S-12-CJR-C | 122095 | 74092 | 58922 | 54940 | 46895 | 16260 |
| Coffee | 16SrRNA | SRR28278095 | SAMN40349107 | 16S-13-COL-A | 91155 | 71023 | 63788 | 62399 | 35736 | 30390 |
|  |  | SRR28278094 | SAMN40349108 | 16S-14-SPB-B | 48703 | 35618 | 31654 | 30228 | 17515 | 14322 |
|  |  | SRR28278093 | SAMN40349109 | 16S-15-LIP-A | 146277 | 114144 | 106376 | 103600 | 65153 | 48129 |
|  |  | SRR28278092 | SAMN40349110 | 16S-16-PRV-A | 83220 | 66660 | 60429 | 57941 | 33942 | 28150 |
|  |  | SRR28278091 | SAMN40349111 | 16S-17-CHU-A1 | 303135 | 227506 | 216390 | 210873 | 155096 | 104458 |
|  |  | SRR28278090 | SAMN40349112 | 16S-18-ACH-A | 92659 | 73436 | 66809 | 64717 | 40024 | 30292 |
|  |  | SRR28278089 | SAMN40349113 | 16S-19-HMP-A | 59022 | 46276 | 40998 | 39468 | 22125 | 18814 |
|  | ITS Fungi | SRR28278203 | SAMN40349168 | ITS-13-COL-A | 77725 | 29657 | 23931 | 21698 | 16105 | 14778 |
|  |  | SRR28278202 | SAMN40349169 | ITS-14-SPB-B | 98888 | 23159 | 17316 | 15748 | 11493 | 10553 |
|  |  | SRR28278201 | SAMN40349170 | ITS-15-LIP-A | 136725 | 19876 | 11079 | 11249 | 7458 | 7084 |
|  |  | SRR28278200 | SAMN40349171 | ITS-16-PRV-A | 59788 | 19556 | 15261 | 14626 | 11046 | 9668 |
|  |  | SRR28278199 | SAMN40349172 | ITS-17-CHU-A1 | 119235 | 29223 | 22632 | 20593 | 14103 | 12999 |
|  |  | SRR28278198 | SAMN40349173 | ITS-18-ACH-A | 110064 | 39019 | 31577 | 29660 | 21428 | 19723 |
|  |  | SRR28278197 | SAMN40349174 | ITS-19-HMP-A | 177301 | 31195 | 23523 | 20344 | 15425 | 14529 |
|  | 16SrRNA Archaea | SRR28292715 | SAMN40349145 | 18S-13-COL-A | 294861 | 195923 | 180717 | 176883 | 151684 | 49782 |
|  |  | SRR28292714 | SAMN40349146 | 18S-14-SPB-B | 358250 | 201734 | 170425 | 157118 | 129000 | 62671 |
|  |  | SRR28292713 | SAMN40349147 | 18S-15-LIP-A | 324875 | 170976 | 136434 | 120245 | 97204 | 49368 |
|  |  | SRR28292712 | SAMN40349148 | 18S-16-PRV-A | 250482 | 136325 | 101449 | 89299 | 70809 | 33756 |
|  |  | SRR28292711 | SAMN40349149 | 18S-17-CHU-A1 | 157060 | 70018 | 52657 | 48429 | 38331 | 17126 |
|  |  | SRR28292710 | SAMN40349150 | 18S-18-ACH-A | 328123 | 160225 | 118942 | 97562 | 67732 | 51544 |
|  |  | SRR28292709 | SAMN40349151 | 18S-19-HMP-A | 516035 | 305385 | 274323 | 264458 | 223336 | 74054 |

**Table S3.** Physicochemical parameters measured at each sampling point from the rhizospheric soils of the cacao and coffee farms. S.C.L. = sandy clay loam; C.L. = clay loam; S.L. = sandy loam; C = clay; L = loam; S = sand.

| **Crops** | **Sample ID** | **Location** | | **Soil parameters** | | | | | | | | | | | | | | | | | |
| --- | --- | --- | --- | --- | --- | --- | --- | --- | --- | --- | --- | --- | --- | --- | --- | --- | --- | --- | --- | --- | --- |
|  |  | **Province** | **District** | **pH** | **EC** | **CaCO3** | **OM** | **P** | **K** | **Sand (%)** | **Silt (%)** | **Clay (%)** | **CEC** | **Ca^+2^** | **Mg^+2^** | **K^+^** | **Na^+^** | **Al^+3^** | **total**  **Cd** | **Soluble**  **Cd** | **Textural Class** |
| Cacao | 01-ARM-A | Bagua | Aramango | 6.13 | 0.28 | 0 | 3.86 | 4.06 | 94.5 | 52.5 | 23 | 24.5 | 19.72 | 16.51 | 1.11 | 0.32 | 0.12 | 0.04 | 1.47 | 0.05 | S.C.L. |
|  | 02-ARM-B | Bagua | Aramango | 6.88 | 0.24 | 0.09 | 3.45 | 5.19 | 121.5 | 45 | 26 | 29 | 17.32 | 14.03 | 2.62 | 0.34 | 0.12 | 0 | 1.3 | 0.04 | C.L |
|  | 03-ARM-C | Bagua | Aramango | 6.84 | 0.23 | 0.18 | 2.68 | 4.26 | 141.67 | 48.67 | 20 | 31.33 | 18.51 | 14.99 | 2.28 | 0.55 | 0.11 | 0.08 | 1.36 | 0.05 | S.C.L. |
|  | 04-CPL-A | Bagua | Copallin | 7.05 | 0.65 | 16.21 | 10.11 | 3.56 | 198.33 | 35.33 | 27.33 | 37.33 | 32.37 | 28.15 | 2.5 | 0.53 | 0.1 | 0 | 2.88 | 0.08 | C.L. |
|  | 05-CPL-B | Bagua | Copallin | 7.66 | 0.62 | 27.12 | 6.23 | 2.5 | 155 | 25.33 | 28.67 | 46 | 24.37 | 21.22 | 2.55 | 0.43 | 0.17 | 0 | 3.65 | 0.07 | S.C.L. |
|  | 06-CPL-C | Bagua | Copallin | 7.82 | 0.56 | 35.32 | 9.21 | 3.82 | 199 | 34 | 28.67 | 37.33 | 24.75 | 21.6 | 2.5 | 0.57 | 0.08 | 0 | 3.96 | 0.06 | C. |
|  | 07-LPC-A | Bagua | La Peca | 6.6 | 0.44 | 0 | 5.59 | 9.52 | 162.67 | 44.67 | 20.67 | 34.67 | 20.48 | 15.3 | 3.76 | 0.47 | 0.16 | 0 | 1.29 | 0.06 | C. |
|  | 08-LPC-B | Bagua | La Peca | 6.31 | 0.29 | 0 | 4.34 | 6.89 | 91.33 | 54 | 19.33 | 26.67 | 16.64 | 11.18 | 3.81 | 0.26 | 0.1 | 0.05 | 2.22 | 0.1 | C. |
|  | 09-LPC-C | Bagua | La Peca | 6.52 | 0.53 | 0.09 | 8.38 | 5.16 | 215.33 | 30 | 21.33 | 48.67 | 32.48 | 28.1 | 2.33 | 0.57 | 0.21 | 0 | 1.55 | 0.08 | C.L. |
|  | 10-CJR-A | Utcubamba | Cajaruro | 6.38 | 0.54 | 0.31 | 2.75 | 2.18 | 158 | 50.5 | 15.5 | 34 | 22.96 | 17.99 | 2.41 | 0.4 | 0.21 | 0.06 | 1.06 | 0.07 | S.C.L. |
|  | 11-CJR-B | Utcubamba | Cajaruro | 6.46 | 0.44 | 0.09 | 2.25 | 3.07 | 164.5 | 49.5 | 16 | 34.5 | 27.72 | 21.6 | 2.78 | 0.48 | 0.38 | 0.03 | 0.89 | 0.07 | S.C.L. |
|  | 12-CJR-C | Utcubamba | Cajaruro | 5.91 | 0.26 | 0 | 2.54 | 1.48 | 55.67 | 52 | 17.33 | 30.67 | 16.43 | 13.15 | 1.27 | 0.16 | 0.18 | 0.07 | 1.05 | 0.04 | S.C.L. |
| Coffee | 13-COL-A-1 | Luya | Collonce | 7.11 | 0.29 | 0.54 | 3.6 | 3.05 | 83 | 59 | 23 | 18 | 14.4 | 10.49 | 3.25 | 0.22 | 0.45 | 0 | 0.68 | 0.03 | S.L. |
|  | 14-SPB-B-1 | Luya | San Pablo | 4.87 | 0.2 | 0 | 6.45 | 11.1 | 62.5 | 67 | 22 | 11 | 14.08 | 6.72 | 0.39 | 0.19 | 0.54 | 0.13 | 0.39 | 0.02 | S.L. |
|  | 15-LIP-A-1 | Luya | Limapampa | 4.78 | 0.91 | 0 | 4.43 | 8.4 | 96 | 57 | 25 | 18 | 14.88 | 7.05 | 2.55 | 0.23 | 0.93 | 0.15 | 0.44 | 0.02 | S.L. |
|  | 16--PRV-A-1 | Luya | El Progreso | 5.15 | 0.56 | 0 | 4.57 | 7.7 | 102 | 59 | 23 | 18 | 12 | 6.12 | 2.39 | 0.25 | 0.53 | 0.1 | 0.51 | 0.02 | S.L. |
|  | 17-CHU-A1-1 | Luya | Chuquimal | 5.48 | 0.12 | 0 | 4.32 | 2.07 | 173.67 | 41.67 | 37 | 21.33 | 16.43 | 10.09 | 1.97 | 0.43 | 0.59 | 0.07 | 0.33 | 0.02 | L. |
|  | 18-ACH-A-1 | R. Mendoza | Achamal | 4.05 | 0.22 | 0 | 3.78 | 9.03 | 115.67 | 55 | 33 | 12 | 12.21 | 2.46 | 0.35 | 0.3 | 0.61 | 1.87 | 0.19 | 0.01 | S.L. |
|  | 19-HMB-A-1 | R. Mendoza | Huambo | 4.19 | 0.13 | 0 | 4.16 | 1.1 | 80 | 55 | 25 | 20 | 10.4 | 2.39 | 0.94 | 0.17 | 0.58 | 1.05 | 0.45 | 0.01 | S.C.L. |

**Table S4.** PERMANOVA of the microbiota composition and physicochemical parameters of rhizospheric soils from the cacao and coffee farms. Statistically significant differences are indicated by asterisks (p < 0.05).

| **Crops** | **Microorganisms** | **Parameters** | **Df** | **SumOfSqs** | **F** | **Pr (>F)** |
| --- | --- | --- | --- | --- | --- | --- |
| Cacao | 16S Bacteria | K | 1 | 0.48463 | 0.9901 | 0.601 |
|  |  | Silt | 1 | 0.50539 | 1.0325 | 0.156 |
|  |  | CIC | 1 | 0.51807 | 1.0584 | 0.055 |
|  |  | Ca | 1 | 0.47152 | 0.9633 | 0.875 |
|  |  | Mg | 1 | 0.48702 | 0.995 | 0.569 |
|  |  | Al | 1 | 0.47933 | 0.9793 | 0.737 |
|  |  | Total Cd | 1 | 0.49229 | 1.0058 | 0.429 |
|  |  | Soluble Cd | 1 | 0.48353 | 0.9879 | 0.67 |
|  |  | Textural Class | 2 | 0.9552 | 0.9758 | 0.798 |
|  |  | Residual | 1 | 0.48946 |  |  |
|  | 16S Archaea | K | 1 | 0.39445 | 1.2324 | 0.296 |
|  |  | Silt | 1 | 0.55334 | 1.7288 | **0.045 *** |
|  |  | CIC | 1 | 0.3273 | 1.0226 | 0.489 |
|  |  | Ca | 1 | 0.40286 | 1.2587 | 0.284 |
|  |  | Mg | 1 | 0.46346 | 1.448 | 0.151 |
|  |  | Al | 1 | 0.28718 | 0.8973 | 0.655 |
|  |  | Total Cd | 1 | 0.32982 | 1.0305 | 0.469 |
|  |  | Soluble Cd | 1 | 0.41681 | 1.3023 | 0.217 |
|  |  | Textural Class | 2 | 0.45809 | 0.7156 | 0.907 |
|  |  | Residual | 1 | 0.32007 |  |  |
|  | ITS Fungi | K | 1 | 0.49611 | 1.1594 | 0.132 |
|  |  | Silt | 1 | 0.58149 | 1.3589 | **0.023 *** |
|  |  | CIC | 1 | 0.44791 | 1.0468 | 0.338 |
|  |  | Ca | 1 | 0.46484 | 1.0864 | 0.26 |
|  |  | Mg | 1 | 0.45303 | 1.0587 | 0.31 |
|  |  | Al | 1 | 0.44866 | 1.0485 | 0.338 |
|  |  | Total Cd | 1 | 0.5082 | 1.1877 | 0.103 |
|  |  | Soluble Cd | 1 | 0.45251 | 1.0575 | 0.338 |
|  |  | Textural Class | 2 | 0.74929 | 0.8756 | 0.871 |
|  |  | Residual | 1 | 0.42789 |  |  |
| Coffee | 16S Bacteria | EC | 1 | 0.47776 | 1.0193 | 0.409 |
|  |  | CaCO3 | 1 | 0.50188 | 1.0708 | 0.099 |
|  |  | OM | 1 | 0.48703 | 1.0391 | 0.259 |
|  |  | Silt | 1 | 0.48305 | 1.0306 | 0.266 |
|  |  | Ca | 1 | 0.46435 | 0.9907 | 0.559 |
|  |  | Residual | 1 | 0.4687 | - |  |
|  | 16S Archaea | EC | 1 | 0.46329 | 1.245 | 0.099 |
|  |  | CaCO3 | 1 | 0.38412 | 1.0322 | 0.465 |
|  |  | OM | 1 | 0.50805 | 1.3653 | 0.061 |
|  |  | Silt | 1 | 0.42135 | 1.1323 | 0.267 |
|  |  | Ca | 1 | 0.47092 | 1.2655 | 0.131 |
|  |  | Residual | 1 | 0.37211 | - | - |
|  | ITS Fungi | EC | 1 | 0.42174 | 0.8385 | 0.902 |
|  |  | CaCO3 | 1 | 0.50634 | 1.0067 | 0.403 |
|  |  | OM | 1 | 0.46378 | 0.922 | 0.738 |
|  |  | Silt | 1 | 0.46291 | 0.9203 | 0.765 |
|  |  | Ca | 1 | 0.39267 | 0.7807 | 0.956 |
|  |  | Residual | 1 | 0.50298 |  |  |

**Table S5.** Differential abundance analysis of KEGG metabolic pathways for bacteria in the rhizospheric soils of cacao and coffee from the Amazonas region. The log₂ Fold Change represents the change in abundance between groups; lfcSE is the associated standard error; and P_adj is the adjusted p-value for multiple comparisons.

| **Metabolic pathway** | **Base Mean** | **log2 FoldChange** | **lfcSE** | **Stat** | **P_value** | **P_adj** | **Significance** | **Enriched in** |
| --- | --- | --- | --- | --- | --- | --- | --- | --- |
| Other carbon fixation pathways | 832078.691 | 0.072440609 | 0.01254119 | 5.776214887 | 0.00000001 | 0.00000087 | *** | Cacao |
| Sulfur metabolism | 212794.7356 | -0.183477142 | 0.034991642 | -5.243456127 | 0.00000016 | 0.00000898 | *** | Coffee |
| Terpenoid backbone biosynthesis | 380022.1835 | 0.058383302 | 0.011519152 | 5.068368186 | 0.00000040 | 0.00001525 | *** | Cacao |
| Homologous recombination | 565776.1729 | 0.072421668 | 0.014804143 | 4.891986634 | 0.00000100 | 0.00002358 | *** | Cacao |
| Phenylalanine, tyrosine and tryptophan biosynthesis | 616606.0183 | 0.072918584 | 0.014927107 | 4.884977814 | 0.00000103 | 0.00002358 | *** | Cacao |
| Polyketide sugar unit biosynthesis | 138551.3972 | 0.119552224 | 0.025828819 | 4.628636957 | 0.00000368 | 0.00005908 | *** | Cacao |
| Metabolism of xenobiotics by cytochrome P450 | 77279.36439 | -0.343051266 | 0.074512718 | -4.603929059 | 0.00000415 | 0.00005908 | *** | Coffee |
| Drug metabolism - cytochrome P450 | 82689.92934 | -0.367161128 | 0.079711809 | -4.606107088 | 0.00000410 | 0.00005908 | *** | Coffee |
| Ribosome | 1700531.519 | 0.082856949 | 0.018562229 | 4.463739311 | 0.00000805 | 0.00010202 | *** | Cacao |
| Aminoacyl-tRNA biosynthesis | 860049.5086 | 0.081254373 | 0.018335827 | 4.431453936 | 0.00000936 | 0.00010670 | *** | Cacao |
| Drug metabolism - other enzymes | 153006.6522 | 0.073997079 | 0.017180196 | 4.307114938 | 0.00001654 | 0.00015713 | *** | Cacao |
| Nitrogen metabolism | 402599.4677 | -0.059839376 | 0.013834785 | -4.32528414 | 0.00001523 | 0.00015713 | *** | Coffee |
| Naphthalene degradation | 89759.35253 | -0.277807438 | 0.065420803 | -4.24646939 | 0.00002172 | 0.00019044 | *** | Coffee |
| Vitamin B6 metabolism | 148843.1787 | 0.058927816 | 0.013970443 | 4.218034967 | 0.00002464 | 0.00020067 | *** | Cacao |
| Biofilm formation - Vibrio cholerae | 57359.09415 | 0.173693243 | 0.041569104 | 4.178421629 | 0.00002935 | 0.00022309 | *** | Cacao |
| Lipoic acid metabolism | 71537.91587 | 0.094891324 | 0.023218663 | 4.086855652 | 0.00004373 | 0.00031155 | *** | Cacao |
| Bacterial chemotaxis | 312638.9616 | -0.144245227 | 0.035486572 | -4.064783389 | 0.00004808 | 0.00032240 | *** | Coffee |
| Tyrosine metabolism | 217158.1853 | -0.105885189 | 0.026407094 | -4.009725133 | 0.00006079 | 0.00038500 | *** | Coffee |
| Nicotinate and nicotinamide metabolism | 333391.8968 | 0.05775986 | 0.014522107 | 3.97737459 | 0.00006968 | 0.00039804 | *** | Cacao |
| Pyrimidine metabolism | 1149805.011 | 0.054570255 | 0.013721951 | 3.976858223 | 0.00006983 | 0.00039804 | *** | Cacao |
| RNA polymerase | 506199.9785 | 0.061480922 | 0.015509016 | 3.964205285 | 0.00007364 | 0.00039976 | *** | Cacao |
| Protein export | 481150.1664 | 0.075633572 | 0.019522048 | 3.874264216 | 0.00010695 | 0.00055418 | *** | Cacao |
| Citrate cycle (TCA cycle) | 642308.0585 | 0.032832778 | 0.00850448 | 3.86064512 | 0.00011309 | 0.00056052 | *** | Cacao |
| Photosynthesis | 269803.1473 | 0.07632448 | 0.020013559 | 3.813638492 | 0.00013694 | 0.00064968 | *** | Cacao |
| Histidine metabolism | 399076.0193 | 0.048292764 | 0.012695815 | 3.803833328 | 0.00014247 | 0.00064968 | *** | Cacao |
| DNA replication | 422876.4047 | 0.044230847 | 0.012003124 | 3.684944656 | 0.00022875 | 0.00097777 | *** | Cacao |
| Mismatch repair | 500720.3396 | 0.051073118 | 0.013871716 | 3.681816839 | 0.00023158 | 0.00097777 | *** | Cacao |
| Caprolactam degradation | 54761.40521 | -0.278950228 | 0.076819154 | -3.6312588 | 0.00028204 | 0.00114831 | ** | Coffee |
| Flagellar assembly | 427365.6049 | -0.161666361 | 0.044928569 | -3.598297539 | 0.00032031 | 0.00125914 | ** | Coffee |
| Chlorocyclohexane and chlorobenzene degradation | 48280.38113 | -0.196035034 | 0.054962075 | -3.566732755 | 0.00036146 | 0.00137355 | ** | Coffee |
| Starch and sucrose metabolism | 419455.3223 | -0.102443104 | 0.02896833 | -3.536382815 | 0.00040565 | 0.00146738 | ** | Coffee |
| Chloroalkane and chloroalkene degradation | 132020.6955 | -0.164625485 | 0.046686338 | -3.526202523 | 0.00042156 | 0.00146738 | ** | Coffee |
| Huntington disease | 73906.19891 | -0.146172358 | 0.041476779 | -3.524197393 | 0.00042477 | 0.00146738 | ** | Coffee |
| One carbon pool by folate | 335573.2473 | 0.049581394 | 0.014248272 | 3.479818033 | 0.00050175 | 0.00168235 | ** | Cacao |
| Aminobenzoate degradation | 159369.3883 | -0.257715245 | 0.074678374 | -3.451002365 | 0.00055851 | 0.00181914 | ** | Coffee |
| Porphyrin metabolism | 528107.8563 | -0.069804193 | 0.020344051 | -3.431184545 | 0.00060095 | 0.00190301 | ** | Coffee |
| Toluene degradation | 149621.7583 | 0.070162004 | 0.020854923 | 3.364289823 | 0.00076741 | 0.00236445 | ** | Cacao |
| Phenylalanine metabolism | 221183.4474 | -0.110793957 | 0.033813827 | -3.276587326 | 0.00105070 | 0.00306758 | ** | Coffee |
| Alzheimer disease | 72646.56102 | -0.115201056 | 0.03523214 | -3.269771756 | 0.00107634 | 0.00306758 | ** | Coffee |
| Purine metabolism | 1512654.142 | 0.023913838 | 0.007299466 | 3.276107905 | 0.00105248 | 0.00306758 | ** | Cacao |
| ABC transporters | 1699812.28 | -0.189587813 | 0.058436742 | -3.244325532 | 0.00117729 | 0.00327344 | ** | Coffee |
| Nucleotide excision repair | 287327.5444 | 0.038492428 | 0.012400883 | 3.104007172 | 0.00190919 | 0.00518208 | ** | Cacao |
| Two-component system | 1173378.696 | -0.056112547 | 0.018171139 | -3.088003731 | 0.00201506 | 0.00534225 | ** | Coffee |
| Tetracycline biosynthesis | 124149.3877 | 0.103346686 | 0.035236833 | 2.932916435 | 0.00335794 | 0.00870012 | ** | Cacao |
| Limonene degradation | 89103.3528 | -0.222764299 | 0.076864541 | -2.89814129 | 0.00375381 | 0.00950966 | ** | Coffee |
| Protein processing in endoplasmic reticulum | 61092.67992 | 0.069964146 | 0.024206459 | 2.890308978 | 0.00384863 | 0.00953792 | ** | Cacao |
| Sulfur relay system | 277632.5392 | 0.038474126 | 0.013401936 | 2.870788655 | 0.00409449 | 0.00993132 | ** | Cacao |
| Glutathione metabolism | 279783.2307 | -0.079767438 | 0.027942495 | -2.854699914 | 0.00430775 | 0.01023091 | * | Coffee |
| Methane metabolism | 567025.3933 | -0.095073172 | 0.033721687 | -2.819348006 | 0.00481213 | 0.01097166 | * | Coffee |
| Glyoxylate and dicarboxylate metabolism | 518662.6572 | -0.064610925 | 0.022884377 | -2.823363932 | 0.00475226 | 0.01097166 | * | Coffee |
| Streptomycin biosynthesis | 259185.3182 | 0.03948579 | 0.014056148 | 2.809147281 | 0.00496729 | 0.01110336 | * | Cacao |
| Ascorbate and aldarate metabolism | 104406.8226 | -0.081169364 | 0.029035272 | -2.795543431 | 0.00518125 | 0.01135890 | * | Coffee |
| Biosynthesis of vancomycin group antibiotics | 46779.78983 | 0.087096619 | 0.031446083 | 2.769712782 | 0.00561057 | 0.01206803 | * | Cacao |
| Novobiocin biosynthesis | 108364.8529 | 0.062575104 | 0.022664224 | 2.760963955 | 0.00576310 | 0.01216655 | * | Cacao |
| Fatty acid degradation | 362326.4115 | -0.118359721 | 0.043033865 | -2.750385595 | 0.00595252 | 0.01233794 | * | Coffee |
| Peroxisome | 174829.6979 | -0.072345021 | 0.026497642 | -2.730243785 | 0.00632875 | 0.01288353 | * | Coffee |
| Styrene degradation | 62979.16434 | -0.214435654 | 0.080686453 | -2.657641335 | 0.00786896 | 0.01573792 | * | Coffee |
| PPAR signaling pathway | 143866.3887 | -0.104626769 | 0.039631133 | -2.640014568 | 0.00829025 | 0.01629462 | * | Coffee |
| beta-Alanine metabolism | 199471.7309 | -0.115769152 | 0.044419032 | -2.606296156 | 0.00915273 | 0.01768493 | * | Coffee |
| Nitrotoluene degradation | 70560.01099 | -0.273331703 | 0.106743228 | -2.560646769 | 0.01044775 | 0.01954247 | * | Coffee |
| Pantothenate and CoA biosynthesis | 473509.1456 | 0.021153078 | 0.008261819 | 2.560341411 | 0.01045694 | 0.01954247 | * | Cacao |
| Alanine, aspartate and glutamate metabolism | 641398.4405 | 0.020381231 | 0.008032338 | 2.537397011 | 0.01116802 | 0.02020880 | * | Cacao |
| Butanoate metabolism | 553901.4826 | -0.103542873 | 0.040720419 | -2.542775263 | 0.01099759 | 0.02020880 | * | Coffee |
| Peptidoglycan biosynthesis | 498333.2964 | 0.033593897 | 0.013433069 | 2.500835499 | 0.01239007 | 0.02206981 | * | Cacao |
| Biosynthesis of unsaturated fatty acids | 209803.6158 | -0.035455701 | 0.014247171 | -2.488613486 | 0.01282423 | 0.02249173 | * | Coffee |
| Inositol phosphate metabolism | 114452.3002 | -0.134933385 | 0.054529281 | -2.474512463 | 0.01334182 | 0.02304496 | * | Coffee |
| Arginine and proline metabolism | 784025.0313 | -0.039006461 | 0.015962569 | -2.443620538 | 0.01454071 | 0.02474091 | * | Coffee |
| Tryptophan metabolism | 301936.426 | -0.100418438 | 0.041513765 | -2.418919086 | 0.01556670 | 0.02609712 | * | Coffee |
| Lipopolysaccharide biosynthesis | 380901.5054 | 0.153994983 | 0.063820712 | 2.412931139 | 0.01582481 | 0.02614534 | * | Cacao |
| Benzoate degradation | 208475.5261 | -0.176750965 | 0.075773252 | -2.332630057 | 0.01966757 | 0.03203004 | * | Coffee |
| Fatty acid biosynthesis | 489827.6118 | 0.030755332 | 0.013228491 | 2.324931295 | 0.02007563 | 0.03223412 | * | Cacao |
| Cyanoamino acid metabolism | 157648.3652 | -0.088200787 | 0.039271643 | -2.245915339 | 0.02470943 | 0.03912327 | * | Coffee |
| Glycolysis / Gluconeogenesis | 672846.4261 | -0.040505607 | 0.018207858 | -2.224622276 | 0.02610661 | 0.04076922 | * | Coffee |
| Oxidative phosphorylation | 1205046.29 | 0.037875033 | 0.017147403 | 2.208791225 | 0.02718917 | 0.04179342 | * | Cacao |
| Tropane, piperidine and pyridine alkaloid biosynthesis | 95581.12242 | 0.029549118 | 0.01340456 | 2.204407909 | 0.02749567 | 0.04179342 | * | Cacao |
| Pyruvate metabolism | 763318.6127 | -0.036220674 | 0.017057429 | -2.123454488 | 0.03371579 | 0.05057368 | ns | Cacao & coffee |
| Pathways in cancer | 59360.09901 | -0.072145856 | 0.034423356 | -2.095840317 | 0.03609636 | 0.05275622 | ns | Cacao & coffee |
| Pentose phosphate pathway | 510429.5526 | -0.027749796 | 0.013232575 | -2.097082141 | 0.03598630 | 0.05275622 | ns | Cacao & coffee |
| Lysine degradation | 227013.0899 | -0.076640505 | 0.03669165 | -2.088772374 | 0.03672822 | 0.05300021 | ns | Cacao & coffee |
| Base excision repair | 316117.3177 | -0.022698143 | 0.011198238 | -2.026938847 | 0.04266867 | 0.06080285 | ns | Cacao & coffee |
| Lysine biosynthesis | 450823.6417 | 0.013619186 | 0.006797189 | 2.003649783 | 0.04510759 | 0.06348476 | ns | Cacao & coffee |
| Isoquinoline alkaloid biosynthesis | 51928.86694 | 0.050393488 | 0.026422945 | 1.90718665 | 0.05649642 | 0.07854381 | ns | Cacao & coffee |
| Phosphotransferase system (PTS) | 83619.87423 | -0.164706916 | 0.087673249 | -1.87864505 | 0.06029298 | 0.08281204 | ns | Cacao & coffee |
| Valine, leucine and isoleucine degradation | 524307.0989 | -0.064463506 | 0.035783272 | -1.801498363 | 0.07162437 | 0.09720450 | ns | Cacao & coffee |
| Amino sugar and nucleotide sugar metabolism | 785765.2411 | 0.018355414 | 0.010308012 | 1.780693904 | 0.07496247 | 0.10053790 | ns | Cacao & coffee |
| Folate biosynthesis | 224557.8585 | 0.016788079 | 0.010332188 | 1.62483298 | 0.10419815 | 0.13812313 | ns | Cacao & coffee |
| Galactose metabolism | 289488.9185 | -0.051361269 | 0.031987187 | -1.605682567 | 0.10834367 | 0.14196757 | ns | Cacao & coffee |
| Neomycin, kanamycin and gentamicin biosynthesis | 48942.00392 | -0.059552753 | 0.037702767 | -1.579532689 | 0.11421392 | 0.14795895 | ns | Cacao & coffee |
| RNA degradation | 383354.3089 | 0.030194426 | 0.019672625 | 1.534844822 | 0.12482195 | 0.15988430 | ns | Cacao & coffee |
| Thiamine metabolism | 254707.0328 | 0.018454655 | 0.012231579 | 1.508771211 | 0.13135726 | 0.16455745 | ns | Cacao & coffee |
| Fructose and mannose metabolism | 343279.4569 | 0.027693266 | 0.018320135 | 1.51163004 | 0.13062801 | 0.16455745 | ns | Cacao & coffee |
| Plant-pathogen interaction | 97323.92403 | -0.023482046 | 0.015983039 | -1.469185337 | 0.14178253 | 0.17568704 | ns | Cacao & coffee |
| Bacterial secretion system | 548652.5271 | 0.041741324 | 0.029311002 | 1.424083828 | 0.15442221 | 0.18727800 | ns | Cacao & coffee |
| Phosphatidylinositol signaling system | 81308.20066 | 0.022276542 | 0.015600732 | 1.427916479 | 0.15331590 | 0.18727800 | ns | Cacao & coffee |
| Selenocompound metabolism | 357959.9317 | 0.016239433 | 0.012663613 | 1.282369641 | 0.19971301 | 0.23965561 | ns | Cacao & coffee |
| Sphingolipid metabolism | 57446.39824 | -0.069854829 | 0.055536493 | -1.257818523 | 0.20845740 | 0.24754316 | ns | Cacao & coffee |
| Pentose and glucuronate interconversions | 243364.5655 | -0.033118478 | 0.028405819 | -1.165904698 | 0.24365298 | 0.28635505 | ns | Cacao & coffee |
| Cell cycle - Caulobacter | 409189.4929 | -0.018252691 | 0.01701506 | -1.072737379 | 0.28338896 | 0.32965655 | ns | Cacao & coffee |
| Biotin metabolism | 95358.72536 | 0.040058093 | 0.03819377 | 1.048812214 | 0.29426456 | 0.33885009 | ns | Cacao & coffee |
| Propanoate metabolism | 604494.4508 | -0.017913762 | 0.019658252 | -0.911259163 | 0.36215884 | 0.41286108 | ns | Cacao & coffee |
| Insulin signaling pathway | 43171.18604 | 0.018804747 | 0.021075158 | 0.892270753 | 0.37224783 | 0.42016091 | ns | Cacao & coffee |
| Arachidonic acid metabolism | 63569.37628 | 0.022846575 | 0.029323267 | 0.779127879 | 0.43590439 | 0.48718726 | ns | Cacao & coffee |
| Glycerolipid metabolism | 208079.1438 | -0.015222838 | 0.019793967 | -0.769064537 | 0.44185500 | 0.48904340 | ns | Cacao & coffee |
| Ubiquinone and other terpenoid-quinone biosynthesis | 222770.6263 | 0.015321014 | 0.020970677 | 0.73059226 | 0.46502824 | 0.50974250 | ns | Cacao & coffee |
| Biosynthesis of ansamycins | 53791.90376 | -0.018800503 | 0.026169539 | -0.718411704 | 0.47250347 | 0.51300377 | ns | Cacao & coffee |
| Taurine and hypotaurine metabolism | 109309.942 | -0.013570407 | 0.022883391 | -0.593024328 | 0.55316487 | 0.59491316 | ns | Cacao & coffee |
| Cysteine and methionine metabolism | 553279.3704 | 0.006761679 | 0.012244206 | 0.55223497 | 0.58078738 | 0.61878281 | ns | Cacao & coffee |
| Riboflavin metabolism | 148525.341 | -0.005125262 | 0.009751496 | -0.525587319 | 0.59917497 | 0.63246247 | ns | Cacao & coffee |
| Glycine, serine and threonine metabolism | 673088.0586 | 0.003263889 | 0.007221599 | 0.451962102 | 0.65129628 | 0.68117226 | ns | Cacao & coffee |
| Carbon fixation by Calvin cycle | 394965.0909 | 0.003408558 | 0.009498136 | 0.358866023 | 0.71969532 | 0.74586606 | ns | Cacao & coffee |
| Glycerophospholipid metabolism | 402314.488 | -0.002811863 | 0.011295142 | -0.248944441 | 0.80340376 | 0.81775025 | ns | Cacao & coffee |
| Adipocytokine signaling pathway | 72957.21173 | -0.007114737 | 0.027640533 | -0.257402307 | 0.79686822 | 0.81775025 | ns | Cacao & coffee |
| Valine, leucine and isoleucine biosynthesis | 569704.6985 | -0.0017484 | 0.009432956 | -0.185350195 | 0.85295437 | 0.86050264 | ns | Cacao & coffee |
| C5-Branched dibasic acid metabolism | 217395.8886 | 0.000508371 | 0.01023759 | 0.049657333 | 0.96039546 | 0.96039546 | ns | Cacao & coffee |

**Table S6.** Differential abundance analysis of KEGG metabolic pathways for archaea in the rhizospheric soils of cacao and coffee from the Amazonas region. The log₂ Fold Change represents the change in abundance between groups; lfcSE is the associated standard error; and P_adj is the adjusted p-value for multiple comparisons.

| **Metabolic pathway (KEGG)** | **Base Mean** | **log2 FoldChange** | **lfcSE** | **Stat** | **P_value** | **P_adj** | **Significance** | **Enriched in** |
| --- | --- | --- | --- | --- | --- | --- | --- | --- |
| Carbon fixation by Calvin cycle | 218390.65 | -0.23402667 | 0.04209635 | -5.55930957 | 2.71E-08 | 2.95E-06 | *** | Coffee |
| Lysine biosynthesis | 255918.895 | -0.10336695 | 0.02424457 | -4.26350919 | 2.01E-05 | 0.00085357 | *** | Coffee |
| Isoquinoline alkaloid biosynthesis | 26629.9782 | -0.27078685 | 0.06540571 | -4.14011003 | 3.47E-05 | 0.00085357 | *** | Coffee |
| Chlorocyclohexane and chlorobenzene degradation | 31973.1278 | 0.61461763 | 0.14906792 | 4.12307112 | 3.74E-05 | 0.00085357 | *** | Cacao |
| Tyrosine metabolism | 113397.365 | 0.35534526 | 0.08730547 | 4.07013746 | 4.70E-05 | 0.00085357 | *** | Cacao |
| Nitrogen metabolism | 319312.653 | 0.39616316 | 0.09666255 | 4.09841428 | 4.16E-05 | 0.00085357 | *** | Cacao |
| Chloroalkane and chloroalkene degradation | 66309.4927 | 0.77321166 | 0.19632418 | 3.93844336 | 8.20E-05 | 0.00089571 | *** | Cacao |
| Naphthalene degradation | 47790.5406 | 0.77734952 | 0.20174923 | 3.8530483 | 0.00011666 | 0.00089571 | *** | Cacao |
| Cysteine and methionine metabolism | 374332.232 | 0.08343273 | 0.02157322 | 3.86742107 | 0.00010999 | 0.00089571 | *** | Cacao |
| Insulin signaling pathway | 24330.5199 | -0.19319551 | 0.04901563 | -3.94150822 | 8.10E-05 | 0.00089571 | *** | Coffee |
| Fructose and mannose metabolism | 178402.8 | 0.19534973 | 0.05064915 | 3.85691988 | 0.00011482 | 0.00089571 | *** | Cacao |
| Alzheimer disease | 34008.1376 | 0.63588756 | 0.1621139 | 3.92247401 | 8.76E-05 | 0.00089571 | *** | Cacao |
| Metabolism of xenobiotics by cytochrome P450 | 46437.7676 | 0.82589457 | 0.2127667 | 3.88169093 | 0.00010373 | 0.00089571 | *** | Cacao |
| Citrate cycle (TCA cycle) | 357825.124 | -0.14397351 | 0.03749756 | -3.83954382 | 0.00012326 | 0.00089571 | *** | Coffee |
| Drug metabolism - cytochrome P450 | 47045.8546 | 0.80815572 | 0.2071796 | 3.90074947 | 9.59E-05 | 0.00089571 | *** | Cacao |
| Phenylalanine, tyrosine and tryptophan biosynthesis | 370971.812 | -0.1697233 | 0.04503716 | -3.76851664 | 0.00016422 | 0.00111875 | ** | Coffee |
| RNA degradation | 174345.897 | -0.20239192 | 0.05402564 | -3.74621935 | 0.00017952 | 0.00113841 | ** | Coffee |
| Parkinson disease | 29780.2276 | 0.93418393 | 0.25014124 | 3.73462577 | 0.00018799 | 0.00113841 | ** | Cacao |
| RNA polymerase | 264846.069 | -0.15737331 | 0.04302568 | -3.65766028 | 0.00025453 | 0.00146019 | ** | Coffee |
| Nicotinate and nicotinamide metabolism | 191492.653 | 0.06926968 | 0.01914878 | 3.61744644 | 0.00029752 | 0.0014741 | ** | Cacao |
| Dioxin degradation | 30396.8407 | 0.77611343 | 0.21380935 | 3.62993217 | 0.0002835 | 0.0014741 | ** | Cacao |
| Bisphenol degradation | 28887.4029 | 1.076268 | 0.29693662 | 3.62457145 | 0.00028944 | 0.0014741 | ** | Cacao |
| Nucleotide excision repair | 183522.428 | 0.12990831 | 0.03645729 | 3.56330136 | 0.00036622 | 0.00173556 | ** | Cacao |
| Arginine and proline metabolism | 408328.384 | 0.0996836 | 0.02825316 | 3.52822889 | 0.00041835 | 0.00190001 | ** | Cacao |
| Butanoate metabolism | 315262.795 | 0.07169545 | 0.02070836 | 3.46214967 | 0.00053588 | 0.00233643 | ** | Cacao |
| Fluorobenzoate degradation | 31281.7924 | 0.5980493 | 0.17653751 | 3.38766141 | 0.00070491 | 0.00274412 | ** | Cacao |
| Ubiquinone and other terpenoid-quinone biosynthesis | 142662.325 | 0.38392929 | 0.11267444 | 3.4074212 | 0.0006558 | 0.00274412 | ** | Cacao |
| Terpenoid backbone biosynthesis | 188063.017 | -0.13306758 | 0.03921567 | -3.39322523 | 0.00069075 | 0.00274412 | ** | Coffee |
| Retinol metabolism | 44071.2851 | 0.84018074 | 0.25650525 | 3.27549137 | 0.00105478 | 0.00396453 | ** | Cacao |
| Cardiac muscle contraction | 28647.6444 | 0.9640013 | 0.29798299 | 3.23508839 | 0.00121605 | 0.00441832 | ** | Cacao |
| Atrazine degradation | 40892.0025 | 1.14034294 | 0.36791951 | 3.09943593 | 0.0019389 | 0.00681741 | ** | Cacao |
| Pentose phosphate pathway | 191640.172 | -0.30451116 | 0.10133248 | -3.00506977 | 0.0026552 | 0.00904427 | ** | Coffee |
| Other carbon fixation pathways | 467732.375 | -0.06085418 | 0.02080077 | -2.92557318 | 0.00343822 | 0.01135655 | * | Coffee |
| Porphyrin metabolism | 537653.338 | 0.28183088 | 0.09817015 | 2.87084082 | 0.00409382 | 0.01312429 | * | Cacao |
| Non-homologous end-joining | 47393.2067 | 0.5473045 | 0.19268584 | 2.84039809 | 0.00450573 | 0.01403212 | * | Cacao |
| Thiamine metabolism | 141699.584 | -0.11129589 | 0.03936485 | -2.82729112 | 0.00469436 | 0.01421349 | * | Coffee |
| Glutathione metabolism | 82568.4116 | 0.31260308 | 0.11532023 | 2.71073923 | 0.00671334 | 0.01977714 | * | Cacao |
| Ribosome | 1328469.75 | -0.17086964 | 0.0639184 | -2.67324636 | 0.00751211 | 0.02154788 | * | Coffee |
| Streptomycin biosynthesis | 90813.7571 | -0.35741195 | 0.13456116 | -2.65613003 | 0.00790431 | 0.02209154 | * | Coffee |
| Pyruvate metabolism | 320348.601 | -0.2395239 | 0.09190817 | -2.6061219 | 0.00915739 | 0.02495388 | * | Coffee |
| Phosphatidylinositol signaling system | 26596.5828 | -0.23777958 | 0.0915563 | -2.59708602 | 0.00940184 | 0.02499513 | * | Coffee |
| DNA replication | 173900.208 | -0.16388445 | 0.06491409 | -2.52463607 | 0.01158182 | 0.02944759 | * | Coffee |
| Peroxisome | 76397.7304 | -0.2785535 | 0.11038066 | -2.52357168 | 0.01161694 | 0.02944759 | * | Coffee |
| Glycolysis / Gluconeogenesis | 296615.068 | -0.24926942 | 0.09922448 | -2.51217656 | 0.0119989 | 0.02972456 | * | Coffee |
| Toluene degradation | 120607.905 | 0.32121446 | 0.13743796 | 2.33715971 | 0.01943088 | 0.04706591 | * | Cacao |
| Pyrimidine metabolism | 823267.461 | -0.09300185 | 0.04061758 | -2.28969457 | 0.02203903 | 0.05222292 | ns | Cacao & coffee |
| Histidine metabolism | 191256.589 | -0.10776192 | 0.05002389 | -2.15420915 | 0.03122377 | 0.07241258 | ns | Cacao & coffee |
| Sulfur relay system | 197527.695 | 0.13092106 | 0.06262668 | 2.09049984 | 0.03657292 | 0.08305101 | ns | Cacao & coffee |
| Ribosome biogenesis in eukaryotes | 159426.568 | -0.29048969 | 0.14373232 | -2.02104638 | 0.04327496 | 0.09626472 | ns | Cacao & coffee |
| Protein export | 242204.663 | -0.05631673 | 0.02807726 | -2.00577735 | 0.04488001 | 0.09783842 | ns | Cacao & coffee |
| Galactose metabolism | 90818.3463 | -0.30091685 | 0.15180058 | -1.98231683 | 0.04744379 | 0.10139948 | ns | Cacao & coffee |
| Benzoate degradation | 85639.1224 | 0.21022265 | 0.10804707 | 1.94565809 | 0.05169582 | 0.1083624 | ns | Cacao & coffee |
| Huntington disease | 88641.9133 | 0.2406703 | 0.12546452 | 1.91823398 | 0.05508135 | 0.11328051 | ns | Cacao & coffee |
| Inositol phosphate metabolism | 85835.363 | -0.11257255 | 0.05982494 | -1.88169955 | 0.05987682 | 0.12086247 | ns | Cacao & coffee |
| Aminoacyl-tRNA biosynthesis | 615811.854 | -0.10142269 | 0.05475177 | -1.85240953 | 0.06396704 | 0.12677104 | ns | Cacao & coffee |
| Pantothenate and CoA biosynthesis | 236137.765 | 0.04145494 | 0.02260461 | 1.83391577 | 0.0666665 | 0.12976158 | ns | Cacao & coffee |
| Propanoate metabolism | 244168.528 | -0.13167233 | 0.07285314 | -1.8073666 | 0.07070514 | 0.13520807 | ns | Cacao & coffee |
| Vitamin B6 metabolism | 84115.9752 | 0.06571874 | 0.03802169 | 1.72845395 | 0.08390687 | 0.15768705 | ns | Cacao & coffee |
| Riboflavin metabolism | 85743.3449 | 0.08727666 | 0.05102169 | 1.71057941 | 0.08715878 | 0.15833846 | ns | Cacao & coffee |
| Tropane, piperidine and pyridine alkaloid biosynthesis | 62281.8058 | 0.10009505 | 0.05849712 | 1.7111107 | 0.08706068 | 0.15833846 | ns | Cacao & coffee |
| Glycine, serine and threonine metabolism | 370515.775 | -0.04783669 | 0.0282437 | -1.69371184 | 0.09032005 | 0.16139157 | ns | Cacao & coffee |
| Cyanoamino acid metabolism | 37454.7534 | -0.39610586 | 0.23583751 | -1.67957106 | 0.0930408 | 0.16357173 | ns | Cacao & coffee |
| Purine metabolism | 1006437.4 | -0.0499436 | 0.03107318 | -1.6072895 | 0.10799088 | 0.18684136 | ns | Cacao & coffee |
| Base excision repair | 196435.478 | -0.04343834 | 0.02914834 | -1.4902505 | 0.13615838 | 0.23189474 | ns | Cacao & coffee |
| Starch and sucrose metabolism | 55481.6488 | -0.82601525 | 0.56553737 | -1.46058474 | 0.14412944 | 0.24169398 | ns | Cacao & coffee |
| Alanine, aspartate and glutamate metabolism | 514034.587 | 0.09385725 | 0.0700247 | 1.34034491 | 0.18013324 | 0.29749277 | ns | Cacao & coffee |
| C5-Branched dibasic acid metabolism | 180314.416 | 0.08521664 | 0.06752187 | 1.26205981 | 0.20692727 | 0.33664286 | ns | Cacao & coffee |
| Lipopolysaccharide biosynthesis | 45694.7501 | -0.33683881 | 0.28175174 | -1.19551636 | 0.23188535 | 0.37169857 | ns | Cacao & coffee |
| Proteasome | 86503.4714 | 0.24037569 | 0.21841589 | 1.10054122 | 0.27109638 | 0.4282537 | ns | Cacao & coffee |
| Protein processing in endoplasmic reticulum | 228834.506 | 0.21815399 | 0.2114134 | 1.03188346 | 0.30212672 | 0.47045446 | ns | Cacao & coffee |
| Aminobenzoate degradation | 46083.0224 | 0.15580582 | 0.15696439 | 0.99261893 | 0.3208957 | 0.49264269 | ns | Cacao & coffee |
| Cell cycle - Caulobacter | 77158.3082 | -0.24219084 | 0.24793992 | -0.9768126 | 0.32866194 | 0.49755765 | ns | Cacao & coffee |
| Bacterial secretion system | 170904.554 | -0.08101323 | 0.08513634 | -0.95157048 | 0.34131486 | 0.50963451 | ns | Cacao & coffee |
| Fatty acid biosynthesis | 75853.4132 | -0.24530038 | 0.26744053 | -0.91721471 | 0.35903014 | 0.52883495 | ns | Cacao & coffee |
| Drug metabolism - other enzymes | 88058.4447 | -0.02801507 | 0.03085354 | -0.90800182 | 0.36387726 | 0.52883495 | ns | Cacao & coffee |
| Oxidative phosphorylation | 641283.932 | 0.04410277 | 0.04939432 | 0.89287134 | 0.37192608 | 0.53246483 | ns | Cacao & coffee |
| Biosynthesis of unsaturated fatty acids | 53115.1357 | -0.13309762 | 0.15193787 | -0.87600028 | 0.38102988 | 0.53246483 | ns | Cacao & coffee |
| Basal transcription factors | 119399.621 | 0.20688894 | 0.23587461 | 0.87711408 | 0.38042468 | 0.53246483 | ns | Cacao & coffee |
| Novobiocin biosynthesis | 66657.0899 | -0.03234416 | 0.0379519 | -0.8522407 | 0.39408051 | 0.54005215 | ns | Cacao & coffee |
| Two-component system | 357357.508 | 0.11280015 | 0.13299949 | 0.84812466 | 0.39636855 | 0.54005215 | ns | Cacao & coffee |
| Tryptophan metabolism | 83135.5534 | -0.13327025 | 0.17136661 | -0.77769088 | 0.43675127 | 0.58772702 | ns | Cacao & coffee |
| beta-Alanine metabolism | 35961.6393 | -0.21259695 | 0.29014013 | -0.73273886 | 0.46371772 | 0.60897869 | ns | Cacao & coffee |
| Biotin metabolism | 77624.4037 | 0.06865592 | 0.09366316 | 0.73300884 | 0.46355304 | 0.60897869 | ns | Cacao & coffee |
| Homologous recombination | 128764.805 | 0.1098411 | 0.16199998 | 0.67803155 | 0.49775169 | 0.63087133 | ns | Cacao & coffee |
| Fatty acid degradation | 113683.997 | 0.08032413 | 0.11690318 | 0.68709962 | 0.49201995 | 0.63087133 | ns | Cacao & coffee |
| One carbon pool by folate | 154449.019 | 0.0297568 | 0.04300413 | 0.69195215 | 0.48896738 | 0.63087133 | ns | Cacao & coffee |
| Cell cycle | 20042.3299 | -0.12657435 | 0.21175979 | -0.59772605 | 0.55002274 | 0.65165738 | ns | Cacao & coffee |
| Phenylalanine metabolism | 60753.9223 | -0.11793955 | 0.18742749 | -0.62925427 | 0.5291826 | 0.65165738 | ns | Cacao & coffee |
| Amino sugar and nucleotide sugar metabolism | 291657.005 | -0.05027233 | 0.07887791 | -0.63734362 | 0.52390105 | 0.65165738 | ns | Cacao & coffee |
| Mismatch repair | 155277.751 | -0.06772075 | 0.11093128 | -0.61047481 | 0.54154733 | 0.65165738 | ns | Cacao & coffee |
| mTOR signaling pathway | 20042.3299 | -0.12657435 | 0.21175979 | -0.59772605 | 0.55002274 | 0.65165738 | ns | Cacao & coffee |
| Hepatitis C | 20042.3299 | -0.12657435 | 0.21175979 | -0.59772605 | 0.55002274 | 0.65165738 | ns | Cacao & coffee |
| Lysine degradation | 56619.0437 | -0.1092925 | 0.19014659 | -0.57478021 | 0.56543995 | 0.66271994 | ns | Cacao & coffee |
| Various types of N-glycan biosynthesis | 20404.3522 | -0.11043341 | 0.20539004 | -0.53767655 | 0.59080037 | 0.67613571 | ns | Cacao & coffee |
| ABC transporters | 541753.769 | 0.07868381 | 0.15046711 | 0.52293026 | 0.60102278 | 0.67613571 | ns | Cacao & coffee |
| Nucleocytoplasmic transport | 223297.534 | -0.08670768 | 0.1623968 | -0.53392478 | 0.59339358 | 0.67613571 | ns | Cacao & coffee |
| Valine, leucine and isoleucine biosynthesis | 382801.921 | -0.0212454 | 0.04070318 | -0.52195918 | 0.60169875 | 0.67613571 | ns | Cacao & coffee |
| Vibrio cholerae infection | 35459.0797 | 0.10092055 | 0.25928813 | 0.38922166 | 0.69711218 | 0.77535947 | ns | Cacao & coffee |
| Peptidoglycan biosynthesis | 80635.7283 | -0.10770224 | 0.30348128 | -0.35488925 | 0.72267257 | 0.79566979 | ns | Cacao & coffee |
| Sulfur metabolism | 92882.5069 | 0.03096233 | 0.09515539 | 0.32538704 | 0.74488816 | 0.8119281 | ns | Cacao & coffee |
| Phagosome | 35736.3337 | 0.07941516 | 0.25626279 | 0.30989735 | 0.75663902 | 0.81401599 | ns | Cacao & coffee |
| Folate biosynthesis | 72892.4073 | 0.03593372 | 0.11851607 | 0.303197 | 0.76173974 | 0.81401599 | ns | Cacao & coffee |
| Glyoxylate and dicarboxylate metabolism | 221447.422 | 0.01233806 | 0.04738708 | 0.26036752 | 0.79458029 | 0.84086652 | ns | Cacao & coffee |
| N-Glycan biosynthesis | 59460.5149 | -0.03355893 | 0.15926382 | -0.21071284 | 0.83311135 | 0.87316478 | ns | Cacao & coffee |
| Valine, leucine and isoleucine degradation | 205541.042 | 0.01507548 | 0.08068863 | 0.18683524 | 0.85178982 | 0.87589708 | ns | Cacao & coffee |
| mRNA surveillance pathway | 38775.5735 | -0.03956163 | 0.20494281 | -0.19303743 | 0.84692966 | 0.87589708 | ns | Cacao & coffee |
| Biosynthesis of ansamycins | 40723.8753 | -0.0142534 | 0.11483166 | -0.12412427 | 0.90121688 | 0.91806205 | ns | Cacao & coffee |
| Selenocompound metabolism | 193830.636 | -0.00163272 | 0.02091978 | -0.07804666 | 0.93779094 | 0.94647419 | ns | Cacao & coffee |
| Methane metabolism | 509139.257 | 0.00286688 | 0.13166818 | 0.02177353 | 0.98262861 | 0.98262861 | ns | Cacao & coffee |

**Table S7.** Differential abundance analysis of guilds for fungi in the rhizospheric soils of cacao and coffee from the Amazonas region. The log₂ Fold Change represents the change in abundance between groups; lfcSE is the associated standard error; and P_adj is the adjusted p-value for multiple comparisons.

| **Guild** | **Base Mean** | **log2 FoldChange** | **lfcSE** | **Stat** | **P_value** | **P_adj** | **Significance** | **Enriched in** |
| --- | --- | --- | --- | --- | --- | --- | --- | --- |
| Fungal Parasite-\|Plant Saprotroph\|-Undefined Saprotroph | 131.1222288 | 2.984676744 | 1.06056198 | 2.814240753 | 0.00488926 | 0.251985478 | ns | Cacao & coffee |
| Dung Saprotroph-Soil Saprotroph | 11.60155321 | -7.502947691 | 2.701167595 | -2.777668333 | 0.005475047 | 0.251985478 | ns | Cacao & coffee |
| \|Lichen Parasite\| | 10.30805722 | -7.331273559 | 2.721572157 | -2.693764168 | 0.007065013 | 0.251985478 | ns | Cacao & coffee |
| Dung Saprotroph-\|Plant Saprotroph\|-Wood Saprotroph | 11.84869239 | 6.321570426 | 2.523823823 | 2.504758997 | 0.012253486 | 0.294980717 | ns | Cacao & coffee |
| Bryophyte Parasite-Plant Pathogen-Plant Saprotroph-Undefined Saprotroph-\|Wood Saprotroph\| | 6.324513806 | -6.62724442 | 2.690895361 | -2.462839885 | 0.013784146 | 0.294980717 | ns | Cacao & coffee |
| Ectomycorrhizal-Orchid Mycorrhizal-\|Plant Pathogen\|-Plant Saprotroph-Wood Saprotroph | 11.73506994 | 5.44724079 | 2.275230276 | 2.39414922 | 0.01665897 | 0.297084961 | ns | Cacao & coffee |
| Animal Pathogen-Fungal Parasite-Undefined Saprotroph | 487.6117968 | -2.634179912 | 1.144039742 | -2.302524831 | 0.021305593 | 0.325671204 | ns | Cacao & coffee |
| Bryophyte Parasite-\|Plant Saprotroph\|-Wood Saprotroph | 22.86784928 | 3.590422356 | 1.676251348 | 2.141935552 | 0.032198669 | 0.386425227 | ns | Cacao & coffee |
| Undefined Saprotroph-Wood Saprotroph | 5.26403771 | 5.138876335 | 2.422077004 | 2.12168165 | 0.033864481 | 0.386425227 | ns | Cacao & coffee |
| \|Animal Parasite\|-Animal Pathogen-Undefined Saprotroph | 3.233448757 | -5.662571149 | 2.70207783 | -2.095635842 | 0.036114507 | 0.386425227 | ns | Cacao & coffee |
| Animal Parasite-Animal Pathogen-\|Plant Saprotroph\|-Undefined Saprotroph | 39.76239312 | 2.922937289 | 1.455284305 | 2.00849915 | 0.044590276 | 0.433741778 | ns | Cacao & coffee |
| Animal Pathogen-Endophyte-Epiphyte-\|Fungal Parasite\|-Plant Pathogen-Wood Saprotroph | 12.32429499 | -3.764044294 | 2.013807697 | -1.869118039 | 0.061606394 | 0.439458942 | ns | Cacao & coffee |
| Bryophyte Parasite-Dung Saprotroph-Ectomycorrhizal-Fungal Parasite-Leaf Saprotroph-Plant Parasite-Undefined Saprotroph-Wood Saprotroph | 43.49184087 | -3.663530698 | 1.916493922 | -1.911579607 | 0.055930137 | 0.439458942 | ns | Cacao & coffee |
| Ectomycorrhizal-Endomycorrhizal-Orchid Mycorrhizal-\|Plant Pathogen\|-Plant Saprotroph-Undefined Saprotroph | 62.9494014 | 3.241862789 | 1.718149207 | 1.886834261 | 0.059182621 | 0.439458942 | ns | Cacao & coffee |
| Plant Pathogen-Wood Saprotroph | 43.2330855 | 1.981087225 | 1.020187903 | 1.941884647 | 0.052151066 | 0.439458942 | ns | Cacao & coffee |
| Ectomycorrhizal-\|Undefined Saprotroph\| | 5.220474342 | 5.143082623 | 3.041691524 | 1.690862661 | 0.090863036 | 0.486117244 | ns | Cacao & coffee |
| Plant Saprotroph-Undefined Saprotroph-\|Wood Saprotroph\| | 2.457778876 | 4.066297586 | 2.340895865 | 1.737068977 | 0.082374996 | 0.486117244 | ns | Cacao & coffee |
| \|Endophyte\|-Plant Saprotroph | 2.186738243 | 3.897941495 | 2.299138514 | 1.695392196 | 0.090001045 | 0.486117244 | ns | Cacao & coffee |
| \|Fungal Parasite\| | 3.768048714 | 3.103586248 | 1.76240668 | 1.760993239 | 0.078239547 | 0.486117244 | ns | Cacao & coffee |
| Fungal Parasite-\|Plant Pathogen\|-Plant Saprotroph-Wood Saprotroph | 2.434885328 | -5.248006893 | 3.05443792 | -1.718157982 | 0.085767803 | 0.486117244 | ns | Cacao & coffee |
| Dung Saprotroph-Ectomycorrhizal-Endophyte-Plant Saprotroph-\|Undefined Saprotroph\| | 3.340875419 | 4.502472459 | 2.709479793 | 1.661747938 | 0.096563325 | 0.492013131 | ns | Cacao & coffee |
| \|Plant Pathogen\|-Wood Saprotroph | 121.2982515 | 1.674429212 | 1.065651897 | 1.571272211 | 0.116119433 | 0.564762698 | ns | Cacao & coffee |
| Endophyte-Lichen Parasite-Plant Pathogen-Undefined Saprotroph | 1.329768052 | -4.369574303 | 3.062693733 | -1.426709519 | 0.153663641 | 0.71486998 | ns | Cacao & coffee |
| - | 14620.10821 | 0.631124497 | 0.491947584 | 1.282910045 | 0.199523593 | 0.818656629 | ns | Cacao & coffee |
| Animal Parasite-Animal Pathogen-\|Undefined Saprotroph\| | 11.20809461 | 1.64290068 | 1.787577323 | 0.919065519 | 0.358061303 | 0.818656629 | ns | Cacao & coffee |
| Animal Parasite-Dung Saprotroph-Endophyte-Fungal Parasite-Plant Pathogen-\|Undefined Saprotroph\|-Wood Saprotroph | 247.1565226 | -1.324898558 | 1.400866328 | -0.945770864 | 0.344265469 | 0.818656629 | ns | Cacao & coffee |
| Animal Parasite-Fungal Parasite-Plant Pathogen-Plant Saprotroph-\|Wood Saprotroph\| | 33.35451607 | 1.896276216 | 1.570823801 | 1.207185819 | 0.227360591 | 0.818656629 | ns | Cacao & coffee |
| Animal Parasite-Undefined Saprotroph-\|Wood Saprotroph\| | 1.137429921 | 2.962989445 | 2.719807839 | 1.089411319 | 0.275972542 | 0.818656629 | ns | Cacao & coffee |
| Dung Saprotroph-Ectomycorrhizal-Litter Saprotroph-Undefined Saprotroph | 1.735755861 | -3.120031659 | 3.051950902 | -1.022307291 | 0.306635484 | 0.818656629 | ns | Cacao & coffee |
| Dung Saprotroph-Ectomycorrhizal-Soil Saprotroph-Wood Saprotroph | 3.80809021 | -2.8955911 | 2.706415608 | -1.069898907 | 0.284664815 | 0.818656629 | ns | Cacao & coffee |
| Dung Saprotroph-Plant Saprotroph-Wood Saprotroph | 12.1266652 | -2.027277757 | 2.211766462 | -0.91658762 | 0.359358774 | 0.818656629 | ns | Cacao & coffee |
| Dung Saprotroph-Soil Saprotroph-Wood Saprotroph | 1.41766667 | 3.274187526 | 3.102215283 | 1.055435303 | 0.291226284 | 0.818656629 | ns | Cacao & coffee |
| Ectomycorrhizal-Fungal Parasite-\|Undefined Saprotroph\|-Undefined Symbiotroph | 34.37053391 | -0.972861663 | 0.982287659 | -0.990404037 | 0.321976673 | 0.818656629 | ns | Cacao & coffee |
| Endophyte-Plant Pathogen-\|Plant Saprotroph\|-Undefined Saprotroph | 4.566266573 | 2.708770089 | 2.047431766 | 1.323008724 | 0.185832477 | 0.818656629 | ns | Cacao & coffee |
| Endophyte-Plant Saprotroph-Undefined Saprotroph-\|Wood Saprotroph\| | 1.028805364 | -3.997634391 | 3.067999253 | -1.303010223 | 0.192571271 | 0.818656629 | ns | Cacao & coffee |
| Endophyte-\|Plant Pathogen\|-Plant Saprotroph | 17.66021468 | 2.298039991 | 1.931513536 | 1.189761266 | 0.234140237 | 0.818656629 | ns | Cacao & coffee |
| Lichen Parasite-\|Wood Saprotroph\| | 1.478464066 | 3.335868592 | 3.084783872 | 1.08139459 | 0.279521627 | 0.818656629 | ns | Cacao & coffee |
| \|Animal Parasite\|-Animal Pathogen-Endophyte-Plant Saprotroph-Undefined Saprotroph | 71.93733371 | 0.951264782 | 0.918628576 | 1.035527095 | 0.300422819 | 0.818656629 | ns | Cacao & coffee |
| \|Animal Parasite\|-Fungal Parasite-Undefined Saprotroph | 1.612796785 | 3.445129134 | 3.100708221 | 1.111078143 | 0.266534715 | 0.818656629 | ns | Cacao & coffee |
| \|Ectomycorrhizal\|-Undefined Saprotroph | 1.689163516 | 3.499370514 | 3.100265447 | 1.128732547 | 0.259010675 | 0.818656629 | ns | Cacao & coffee |
| \|Undefined Saprotroph\|-Undefined Symbiotroph | 251.4065951 | -1.592231194 | 1.401587917 | -1.136019492 | 0.255948406 | 0.818656629 | ns | Cacao & coffee |
| \|Wood Saprotroph\| | 82.80349771 | -1.114245321 | 0.978005875 | -1.1393033 | 0.254576673 | 0.818656629 | ns | Cacao & coffee |
| Animal Pathogen-Animal Symbiotroph-Endophyte-Plant Pathogen-\|Undefined Saprotroph\| | 0.88953311 | -3.778926024 | 3.071811902 | -1.230194473 | 0.218624289 | 0.818656629 | ns | Cacao & coffee |
| Arbuscular Mycorrhizal | 0.427937381 | -2.750532179 | 3.099769189 | -0.887334511 | 0.37489883 | 0.818656629 | ns | Cacao & coffee |
| Dung Saprotroph-Plant Saprotroph-Soil Saprotroph | 0.427937381 | -2.750532179 | 3.099769189 | -0.887334511 | 0.37489883 | 0.818656629 | ns | Cacao & coffee |
| Ectomycorrhizal-Endophyte-Plant Pathogen-\|Plant Saprotroph\|-Wood Saprotroph | 0.771604023 | -3.580075116 | 3.075809559 | -1.163945637 | 0.244446043 | 0.818656629 | ns | Cacao & coffee |
| Ectomycorrhizal-\|Plant Saprotroph\|-Undefined Saprotroph-Wood Saprotroph | 0.435315641 | -2.799536249 | 3.097949391 | -0.903673978 | 0.366168301 | 0.818656629 | ns | Cacao & coffee |
| \|Lichenized\| | 0.435315641 | -2.799536249 | 3.097949391 | -0.903673978 | 0.366168301 | 0.818656629 | ns | Cacao & coffee |
| \|Plant Pathogen\|-Plant Saprotroph-Wood Saprotroph | 0.489071292 | -2.941954152 | 3.09299002 | -0.95116833 | 0.341518932 | 0.818656629 | ns | Cacao & coffee |
| Animal Pathogen-Undefined Saprotroph | 0.946843605 | 2.656014658 | 3.109379858 | 0.854194334 | 0.392997322 | 0.828763703 | ns | Cacao & coffee |
| \|Plant Saprotroph\|-Undefined Saprotroph | 129.8937147 | 0.884959281 | 1.040452775 | 0.850552089 | 0.395018214 | 0.828763703 | ns | Cacao & coffee |
| \|Plant Pathogen\|-Undefined Saprotroph | 0.915607218 | 2.577689766 | 3.110519251 | 0.828700792 | 0.407273738 | 0.83126099 | ns | Cacao & coffee |
| \|Undefined Saprotroph\| | 505.6313388 | -0.402565588 | 0.490440362 | -0.820824752 | 0.411746098 | 0.83126099 | ns | Cacao & coffee |
| Nematophagous-Undefined Saprotroph-\|Wood Saprotroph\| | 0.94268492 | -2.457868854 | 3.068727583 | -0.800940711 | 0.42316597 | 0.834597976 | ns | Cacao & coffee |
| Plant Pathogen-Undefined Saprotroph-\|Wood Saprotroph\| | 16.56518979 | -1.056005172 | 1.335184496 | -0.790905807 | 0.428998959 | 0.834597976 | ns | Cacao & coffee |
| Plant Saprotroph-Wood Saprotroph | 0.328611642 | -2.397683664 | 3.114485756 | -0.769848974 | 0.441389485 | 0.843369194 | ns | Cacao & coffee |
| Ectomycorrhizal-\|Endophyte\|-Ericoid Mycorrhizal-Orchid Mycorrhizal-Undefined Saprotroph | 20.01904657 | -0.840237431 | 1.236292427 | -0.679642949 | 0.496730568 | 0.866488841 | ns | Cacao & coffee |
| Endomycorrhizal-Plant Pathogen-Undefined Saprotroph | 9.272917649 | -1.61610215 | 2.429665076 | -0.66515429 | 0.505951813 | 0.866488841 | ns | Cacao & coffee |
| Endophyte-Epiphyte-Fungal Parasite-Insect Parasite | 1.144346203 | -2.016201973 | 3.06151176 | -0.658564177 | 0.510175673 | 0.866488841 | ns | Cacao & coffee |
| Endophyte-Ericoid Mycorrhizal-\|Undefined Saprotroph\| | 0.645905433 | 2.124309968 | 3.117564207 | 0.681400551 | 0.495618074 | 0.866488841 | ns | Cacao & coffee |
| Endophyte-Plant Saprotroph-\|Undefined Saprotroph\| | 32.41932743 | 0.727760555 | 1.00740739 | 0.722409387 | 0.470042817 | 0.866488841 | ns | Cacao & coffee |
| Plant Pathogen-\|Plant Saprotroph\|-Undefined Saprotroph-Wood Saprotroph | 4.817012116 | 1.601091561 | 2.362262321 | 0.677778902 | 0.49791189 | 0.866488841 | ns | Cacao & coffee |
| \|Dung Saprotroph\|-Undefined Saprotroph | 27.0363532 | -0.947281899 | 1.33497044 | -0.709590168 | 0.477958318 | 0.866488841 | ns | Cacao & coffee |
| Leaf Saprotroph-Wood Saprotroph | 0.257201341 | -1.844202361 | 3.130264514 | -0.589152244 | 0.55575915 | 0.867648419 | ns | Cacao & coffee |
| Lichenized-Undefined Saprotroph | 28.81194277 | -1.819455516 | 2.994208277 | -0.607658302 | 0.543414124 | 0.867648419 | ns | Cacao & coffee |
| Plant Pathogen-\|Pollen Saprotroph\|-Undefined Parasite-Undefined Saprotroph | 0.470472792 | 1.779706862 | 3.122377718 | 0.569984487 | 0.56868822 | 0.867648419 | ns | Cacao & coffee |
| \|Animal Parasite\|-Plant Pathogen-Undefined Saprotroph | 0.470472792 | 1.779706862 | 3.122377718 | 0.569984487 | 0.56868822 | 0.867648419 | ns | Cacao & coffee |
| \|Animal Parasite\|-Undefined Saprotroph-Wood Saprotroph | 0.444157797 | 1.747642376 | 3.122832718 | 0.559633683 | 0.575729325 | 0.867648419 | ns | Cacao & coffee |
| \|Insect Pathogen\|-Undefined Saprotroph | 0.49301269 | 1.848611067 | 3.121431823 | 0.592231761 | 0.553695404 | 0.867648419 | ns | Cacao & coffee |
| \|Plant Pathogen\| | 66.53057889 | -0.833009082 | 1.427123242 | -0.58369807 | 0.559423466 | 0.867648419 | ns | Cacao & coffee |
| \|Plant Saprotroph\| | 75.7755548 | 0.865713195 | 1.518584627 | 0.570078993 | 0.568624122 | 0.867648419 | ns | Cacao & coffee |
| Animal Pathogen-Endophyte-Endosymbiont-Epiphyte-\|Undefined Saprotroph\| | 0.324092158 | -1.668948248 | 3.118782504 | -0.53512813 | 0.59256126 | 0.877574637 | ns | Cacao & coffee |
| Epiphyte | 18.9107809 | 1.138140089 | 2.162763882 | 0.52624334 | 0.598719145 | 0.877574637 | ns | Cacao & coffee |
| \|Ectomycorrhizal\| | 29.29922672 | -0.739199019 | 1.448767856 | -0.510225994 | 0.609893143 | 0.881872518 | ns | Cacao & coffee |
| Dung Saprotroph-\|Undefined Saprotroph\| | 1.440108311 | -1.409766708 | 3.05097039 | -0.46207158 | 0.64402999 | 0.885472445 | ns | Cacao & coffee |
| Plant Pathogen-Plant Saprotroph-Undefined Saprotroph-\|Wood Saprotroph\| | 0.363198757 | 1.472764613 | 3.127151237 | 0.470960469 | 0.637668965 | 0.885472445 | ns | Cacao & coffee |
| \|Animal Parasite\|-Dung Saprotroph-Nematophagous-Wood Saprotroph | 12.55403628 | -0.634404115 | 1.379007255 | -0.460044073 | 0.645484586 | 0.885472445 | ns | Cacao & coffee |
| \|Plant Pathogen\|-Plant Saprotroph | 8.596500899 | 0.924757947 | 1.937891111 | 0.477198095 | 0.633221067 | 0.885472445 | ns | Cacao & coffee |
| Endophyte-\|Epiphyte\|-Undefined Saprotroph | 0.313648528 | 1.327306259 | 3.129264525 | 0.424159175 | 0.671449732 | 0.88946006 | ns | Cacao & coffee |
| Undefined Saprotroph | 59.14260269 | -0.714543898 | 1.694907699 | -0.421582779 | 0.673329578 | 0.88946006 | ns | Cacao & coffee |
| \|Plant Saprotroph\|-Undefined Saprotroph-Wood Saprotroph | 15.05533904 | 0.729948244 | 1.704436014 | 0.4282638 | 0.668459071 | 0.88946006 | ns | Cacao & coffee |
| Dung Saprotroph-Plant Parasite-\|Plant Saprotroph\|-Undefined Saprotroph-Wood Saprotroph | 0.227334653 | 1.074090084 | 3.131928705 | 0.342948447 | 0.731637244 | 0.91068746 | ns | Cacao & coffee |
| Ectomycorrhizal-Fungal Parasite | 0.248815028 | 1.166333342 | 3.130931809 | 0.372519561 | 0.709506051 | 0.91068746 | ns | Cacao & coffee |
| Ectomycorrhizal-\|Plant Saprotroph\| | 0.242139741 | 1.102103789 | 3.131619752 | 0.351927717 | 0.724892472 | 0.91068746 | ns | Cacao & coffee |
| Undefined Saprotroph-\|Wood Saprotroph\| | 282.492242 | 0.412340569 | 1.092979245 | 0.377262945 | 0.705978207 | 0.91068746 | ns | Cacao & coffee |
| \|Pollen Saprotroph\| | 0.231267318 | 1.072774832 | 3.131943346 | 0.342526896 | 0.731954407 | 0.91068746 | ns | Cacao & coffee |
| Endophyte-Litter Saprotroph-Soil Saprotroph-Undefined Saprotroph | 42.03846589 | -0.354345047 | 1.099268464 | -0.322346231 | 0.747190413 | 0.918958324 | ns | Cacao & coffee |
| Dung Saprotroph-\|Plant Pathogen\|-Plant Saprotroph | 2.472698054 | -0.828989116 | 2.809725748 | -0.295042716 | 0.767961249 | 0.933771064 | ns | Cacao & coffee |
| Animal Pathogen-Endophyte-Plant Saprotroph-\|Undefined Saprotroph\|-Wood Saprotroph | 27.56264818 | 0.400959468 | 1.543110426 | 0.25983848 | 0.794988367 | 0.937334335 | ns | Cacao & coffee |
| Dung Saprotroph-Plant Saprotroph-\|Undefined Saprotroph\| | 41.97226284 | -0.321542544 | 1.22058173 | -0.26343385 | 0.792216203 | 0.937334335 | ns | Cacao & coffee |
| Plant Saprotroph-\|Wood Saprotroph\| | 3.42591573 | -0.651350113 | 2.534352156 | -0.257008526 | 0.797172191 | 0.937334335 | ns | Cacao & coffee |
| Animal Parasite-\|Wood Saprotroph\| | 1.411135162 | 0.749522391 | 3.075303531 | 0.243723061 | 0.807445311 | 0.939094004 | ns | Cacao & coffee |
| Animal Pathogen-Endophyte-\|Plant Pathogen\|-Plant Saprotroph-Wood Saprotroph | 0.131099302 | 0.640795368 | 3.135987117 | 0.204336097 | 0.838090864 | 0.960110172 | ns | Cacao & coffee |
| Animal Pathogen-\|Undefined Saprotroph\| | 0.121045149 | 0.604296422 | 3.136264257 | 0.192680327 | 0.847209332 | 0.960110172 | ns | Cacao & coffee |
| Wood Saprotroph | 59.9337757 | 0.272830729 | 1.562755986 | 0.174583064 | 0.861407257 | 0.960110172 | ns | Cacao & coffee |
| \|Fungal Parasite\|-Wood Saprotroph | 0.114711889 | 0.553391602 | 3.136536107 | 0.176433997 | 0.859952998 | 0.960110172 | ns | Cacao & coffee |
| Plant Pathogen-\|Wood Saprotroph\| | 16.82533953 | -0.237629293 | 1.554661457 | -0.152849543 | 0.878516927 | 0.969085683 | ns | Cacao & coffee |
| Animal Pathogen-Endophyte-Fungal Parasite-\|Undefined Saprotroph\| | 255.5196049 | 0.140721548 | 1.043718274 | 0.134827138 | 0.892748549 | 0.97473566 | ns | Cacao & coffee |
| Animal Parasite-\|Undefined Saprotroph\| | 160.220211 | 0.125446618 | 1.233642934 | 0.101687947 | 0.919004369 | 0.987997064 | ns | Cacao & coffee |
| Animal Pathogen-Dung Saprotroph-Endophyte-Lichen Parasite-Plant Pathogen-\|Undefined Saprotroph\| | 0.049162238 | 0.111326852 | 3.138745339 | 0.035468584 | 0.971706097 | 0.987997064 | ns | Cacao & coffee |
| Animal Pathogen-Plant Pathogen-\|Undefined Saprotroph\| | 58.72501995 | 0.023361681 | 1.552888548 | 0.015044016 | 0.987997064 | 0.987997064 | ns | Cacao & coffee |
| Dung Saprotroph-\|Plant Saprotroph\|-Undefined Saprotroph | 5.133935345 | 0.099135072 | 2.134149032 | 0.046451804 | 0.962950148 | 0.987997064 | ns | Cacao & coffee |
| Endophyte-Fungal Parasite-Lichen Parasite-\|Plant Pathogen\|-Plant Saprotroph-Undefined Saprotroph | 0.067247305 | 0.269838548 | 3.138007067 | 0.085990421 | 0.931474032 | 0.987997064 | ns | Cacao & coffee |
| \|Animal Parasite\|-Animal Pathogen-Clavicipitaceous Endophyte | 0.040348383 | 0.049751769 | 3.138932747 | 0.0158499 | 0.987354139 | 0.987997064 | ns | Cacao & coffee |
| \|Animal Parasite\|-Endophyte-Plant Pathogen-Undefined Saprotroph | 2.368616741 | 0.050751933 | 1.994105255 | 0.02545098 | 0.979695248 | 0.987997064 | ns | Cacao & coffee |
| \|Animal Parasite\|-Undefined Saprotroph | 6.090198895 | 0.057431914 | 2.142889946 | 0.026801149 | 0.978618336 | 0.987997064 | ns | Cacao & coffee |
| \|Dung Saprotroph\| | 0.049162238 | 0.111326852 | 3.138745339 | 0.035468584 | 0.971706097 | 0.987997064 | ns | Cacao & coffee |
| Animal Associated Biotroph-Root Associate Biotroph | 0 | 0 | 0 | 0 | 1 | NA | ns | Cacao & coffee |
| Animal Pathogen-Endophyte-Epiphyte-\|Plant Pathogen\|-Plant Saprotroph-Undefined Saprotroph | 0 | 0 | 0 | 0 | 1 | NA | ns | Cacao & coffee |
| Ectomycorrhizal | 0 | 0 | 0 | 0 | 1 | NA | ns | Cacao & coffee |
| Endophyte-Fungal Parasite-\|Plant Pathogen\|-Plant Saprotroph-Undefined Saprotroph | 0 | 0 | 0 | 0 | 1 | NA | ns | Cacao & coffee |
| Plant Pathogen-\|Plant Saprotroph\|-Undefined Saprotroph | 0 | 0 | 0 | 0 | 1 | NA | ns | Cacao & coffee |
| Plant Saprotroph-\|Undefined Saprotroph\|-Wood Saprotroph | 0 | 0 | 0 | 0 | 1 | NA | ns | Cacao & coffee |
| \|Endophyte\|-Undefined Saprotroph | 0 | 0 | 0 | 0 | 1 | NA | ns | Cacao & coffee |
| \|Plant Pathogen\|-Plant Saprotroph-Undefined Saprotroph | 0 | 0 | 0 | 0 | 1 | NA | ns | Cacao & coffee |
| Algal Parasite-\|Plant Saprotroph\|-Undefined Saprotroph | 0 | 0 | 0 | 0 | 1 | NA | ns | Cacao & coffee |
| Animal Pathogen-Plant Pathogen-Undefined Saprotroph | 0 | 0 | 0 | 0 | 1 | NA | ns | Cacao & coffee |
| Ectomycorrhizal-Fungal Parasite-Soil Saprotroph-Undefined Saprotroph | 0 | 0 | 0 | 0 | 1 | NA | ns | Cacao & coffee |
| Ectomycorrhizal-\|Plant Saprotroph\|-Wood Saprotroph | 0 | 0 | 0 | 0 | 1 | NA | ns | Cacao & coffee |
| Fungal Parasite-Plant Pathogen-Plant Saprotroph | 0 | 0 | 0 | 0 | 1 | NA | ns | Cacao & coffee |
| Plant Saprotroph-\|Pollen Saprotroph\| | 0 | 0 | 0 | 0 | 1 | NA | ns | Cacao & coffee |
