# Supplementary material for: Functional Specialisation and Diversity Shape the Rhizosphere Microbiota of Cacao and Coffee in the Amazonas Region, Peru
Source: Environ Microbiol Rep. 2025 Dec 9;17(6):e70259. doi: 10.1111/1758-2229.70259 (PMC12688408; doi:10.1111/1758-2229.70259)
Supplement: Supplementary file 2 — Figure S1: Workflow of rhizospheric soil collection from coffee (left) and cacao (right) farms in the Amazonas region. Created with BioRender.com. Figure S2: Workflow of physicochemical (A) and molecular (B) analyses of rhizospheric soil samples from cacao and coffee farms in the Amazonas region. Created with BioRender.com. Figure S3: Quality and Phred score for each of the 250 base pairs (bp) of the unprocessed sequences from the 16S rRNA (bacteria), 16S rRNA (archaea) and ITS (fungi) regions. High‐quality values for forward and reverse reads are represented in green, while low‐quality sequences are highlighted in red. Figure S4: Phred quality scores for each of the 250 base pairs (bp) in the 16S rRNA (bacteria), 16S rRNA (archaea) and ITS (fungi) regions after filtering. High‐quality values for forward (A) and reverse (B) reads are displayed in green, while sequences with low quality are highlighted in red. Figure S5: Taxonomic composition of the microbiota (i.e., bacteria, archaea and fungi) associated with cacao rhizospheric soils from Bagua and Utcubamba Provinces at the phylum (A, C, E) and class (B, D, F) levels. Figure S6: Taxonomic composition of the microbiota (i.e., bacteria, archaea and fungi) associated with coffee rhizospheric soils from Luya and Rodríguez de Mendoza Provinces at the phylum (A, C, E) and class (B, D, F) levels. Figure S7: Co‐occurrence network of microbiota associated with the cacao rhizospheric soils in the Amazonas, using a filter threshold of 0.001 and a correlation coefficient of 0.6. Figure S8: Co‐occurrence network of microbiota associated with the coffee rhizospheric soils in the Amazonas region, using a filter threshold of 0.0005 and a correlation coefficient of 0.5. Figure S9: Alpha diversity indices of the microbiota (i.e., bacteria, archaea and fungi), evaluated using the Shannon (A–C) and Simpson indices (D–F), associated with the rhizospheric soils of cacao plantations across the different districts of the Amazonas region. [file EMI4-17-e70259-s001.docx]

**Supplemental figures**


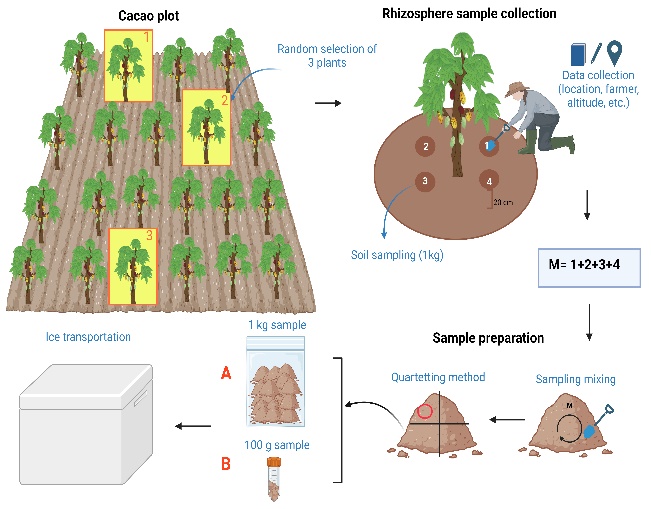

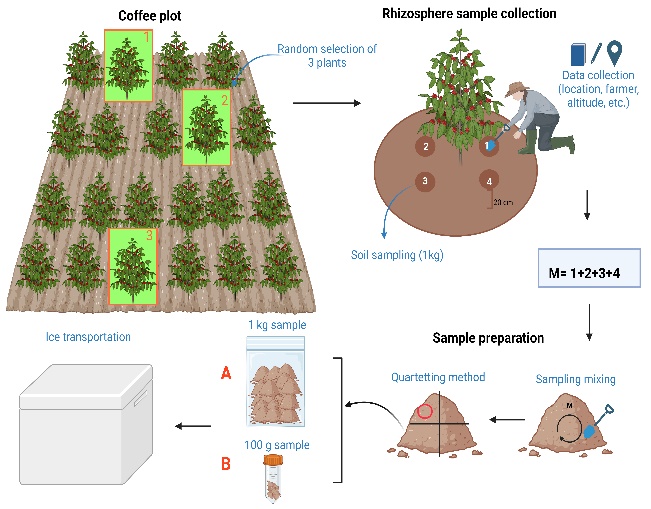


**Figure S1.** Workflow of rhizospheric soil collection from coffee (left) and cacao (right) farms in the Amazonas region. Created with BioRender.com


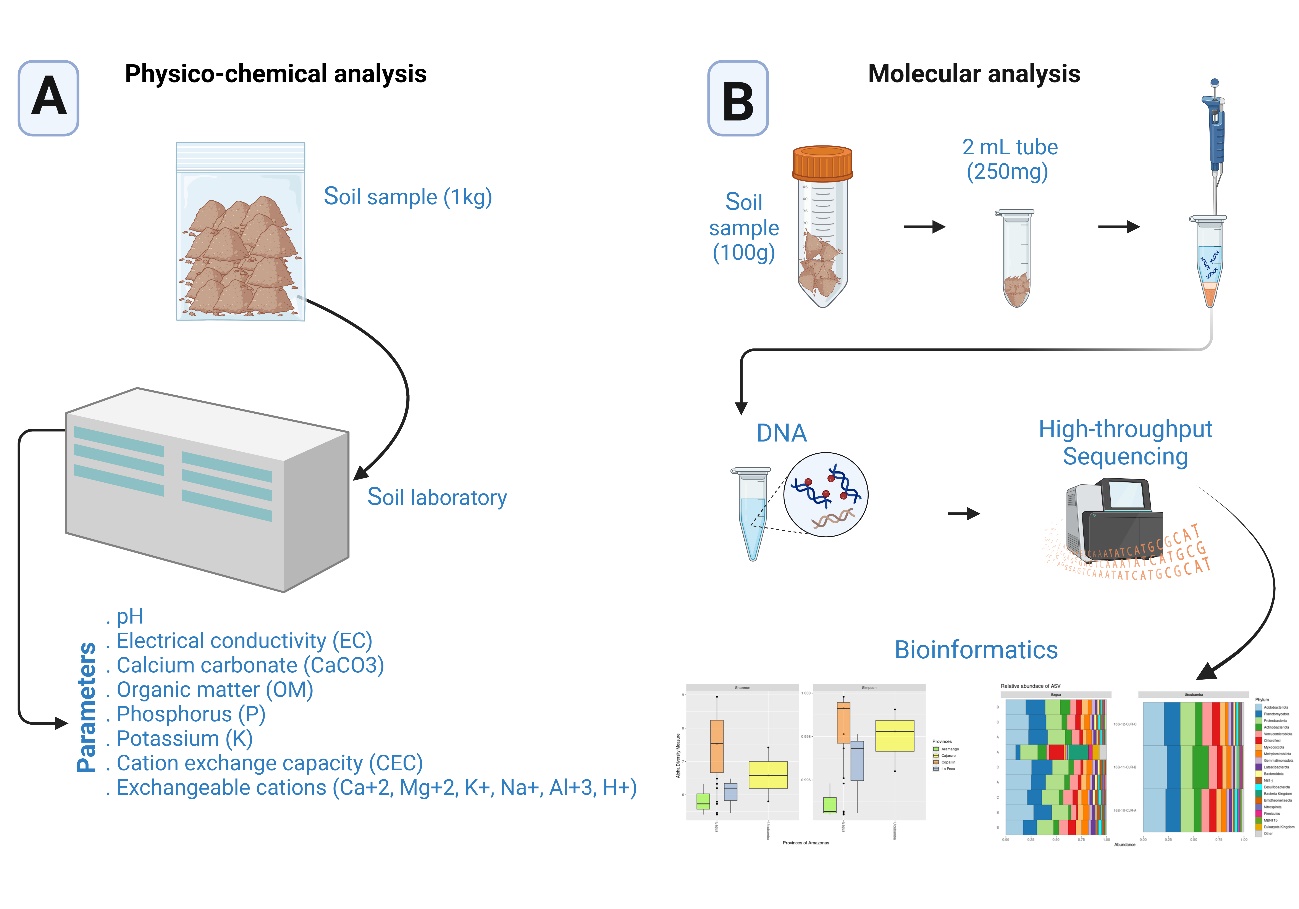


**Figure S2.** Workflow of physicochemical (A) and molecular (B) analyses of rhizospheric soil samples from cacao and coffee farms in the Amazonas region. Created with BioRender.com


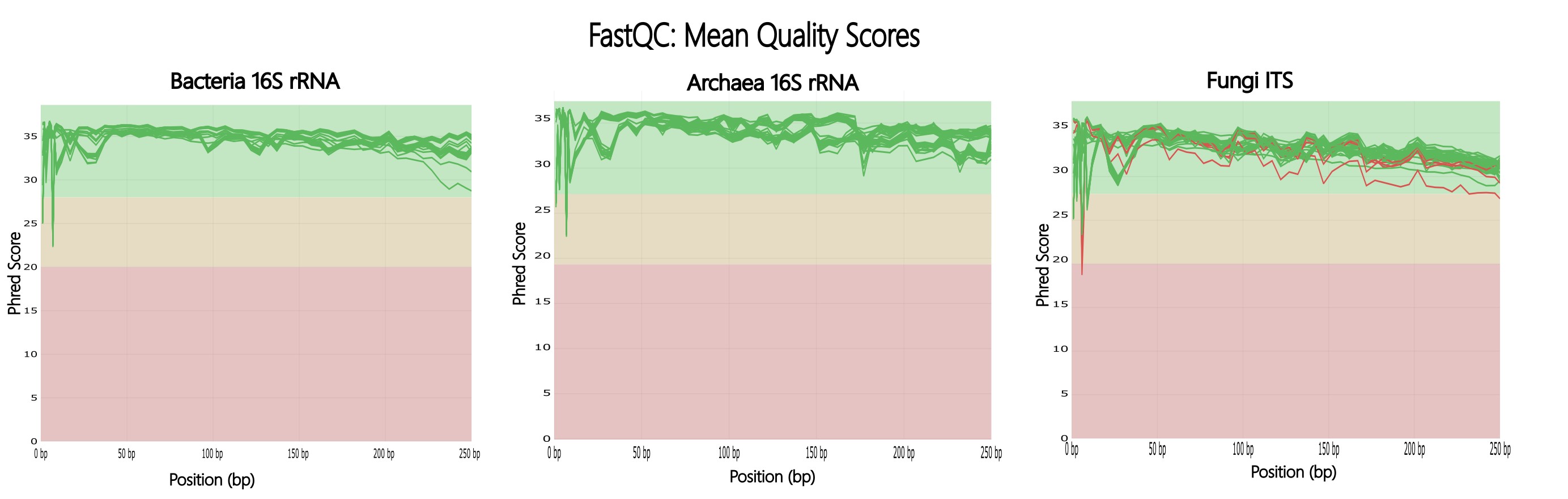


**Figure S3.** Quality and Phred score for each of the 250 base pairs (bp) of the unprocessed sequences from the 16S rRNA (bacteria), 16S rRNA (archaea), and ITS (fungi) regions. High-quality values for forward and reverse reads are represented in green, while low-quality sequences are highlighted in red.


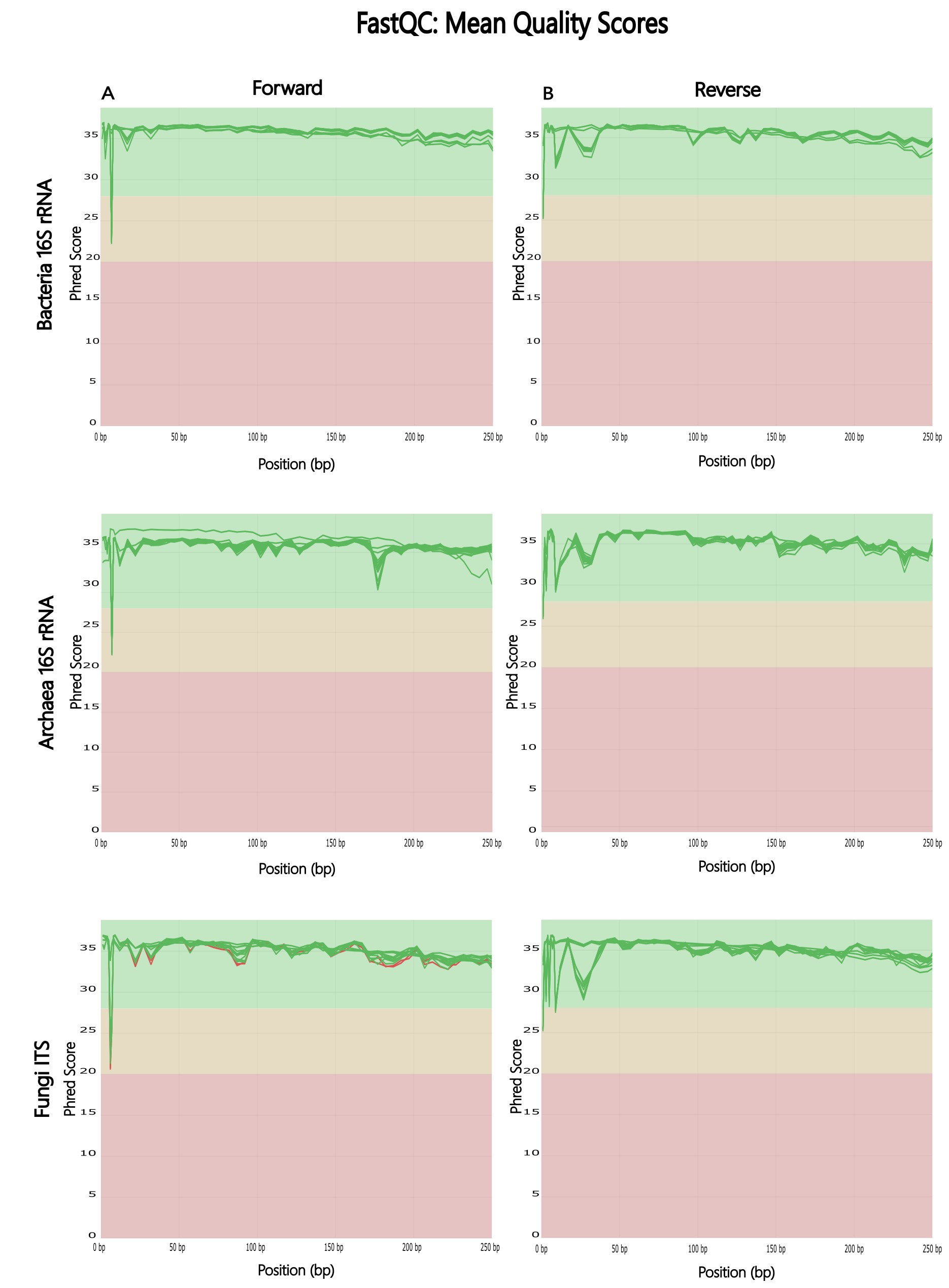


**Figure S4.** Phred quality scores for each of the 250 base pairs (bp) in the 16S rRNA (bacteria), 16S rRNA (archaea), and ITS (fungi) regions after filtering. High-quality values for forward (A) and reverse (B) reads are displayed in green, while sequences with low quality are highlighted in red.





**Figure S5.** Taxonomic composition of the microbiota (i.e., bacteria, archaea, and fungi) associated with cacao rhizospheric soils from Bagua and Utcubamba Provinces at the phylum (A, C, E) and class (B, D, F) levels.


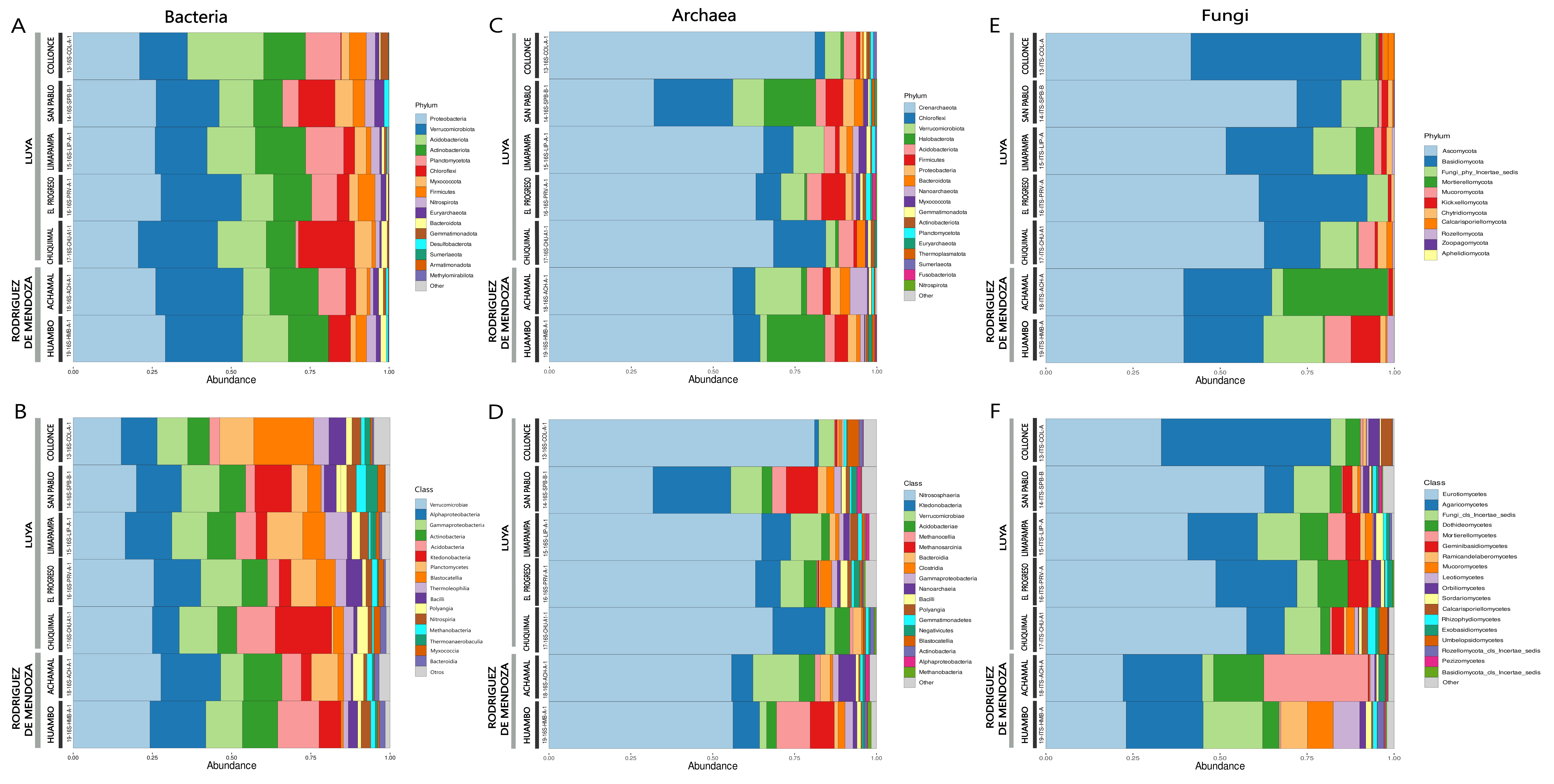


**Figure S6.** Taxonomic composition of the microbiota (i.e., bacteria, archaea, and fungi) associated with coffee rhizospheric soils from Luya and Rodríguez de Mendoza Provinces at the phylum (A, C, E) and class (B, D, F) levels.





**Figure S7**. Co-occurrence network of microbiota associated with the cacao rhizospheric soils in the Amazonas, using a filter threshold of 0.001 and a correlation coefficient of 0.6.





**Figure S8**. Co-occurrence network of microbiota associated with the coffee rhizospheric soils in the Amazonas region, using a filter threshold of 0.0005 and a correlation coefficient of 0.5.


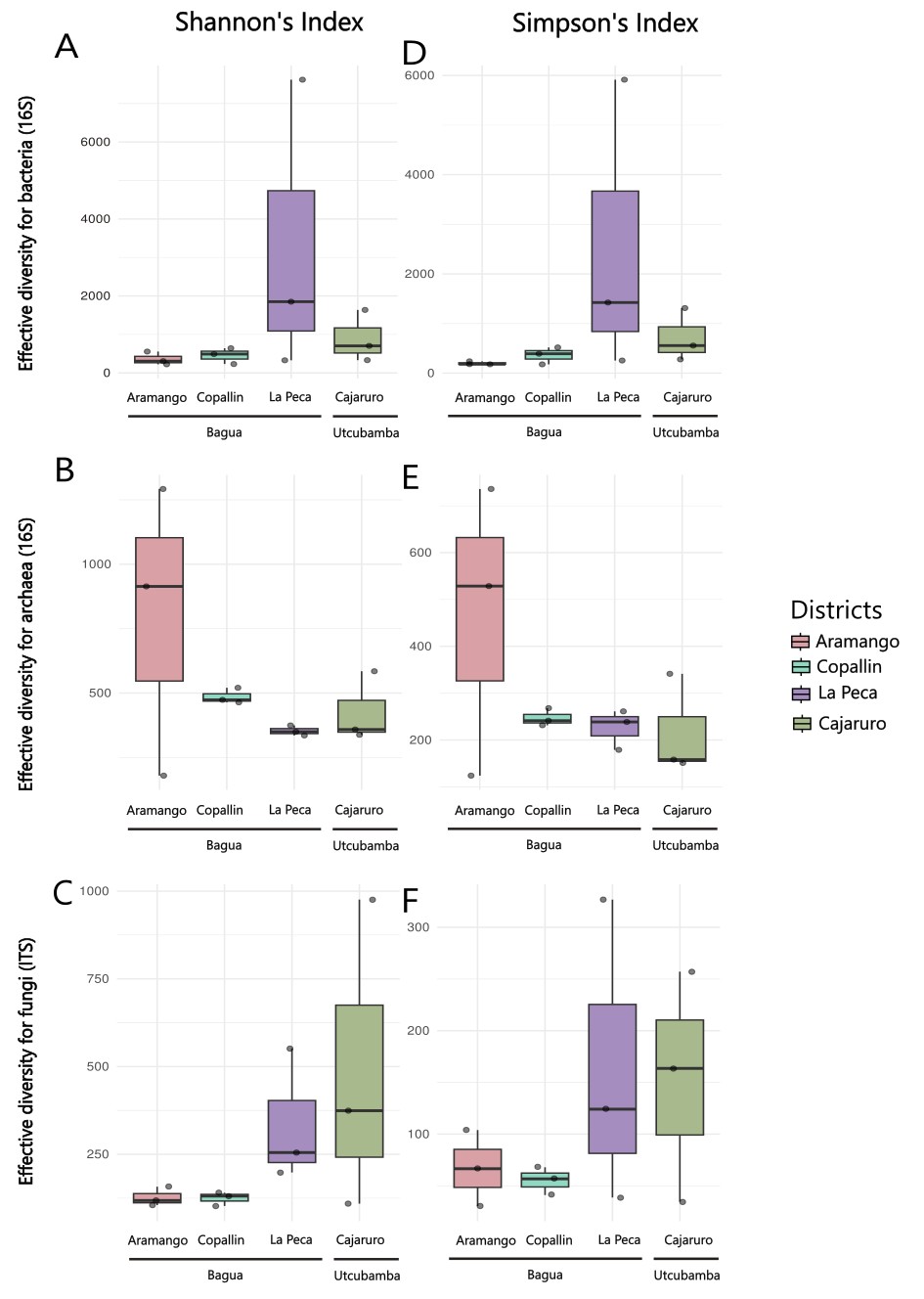


**Figure S9.** Alpha diversity indices of the microbiota (i.e., bacteria, archaea, and fungi), evaluated using the Shannon (A–C) and Simpson indices (D–F), associated with the rhizospheric soils of cacao plantations across the different districts of the Amazonas region.


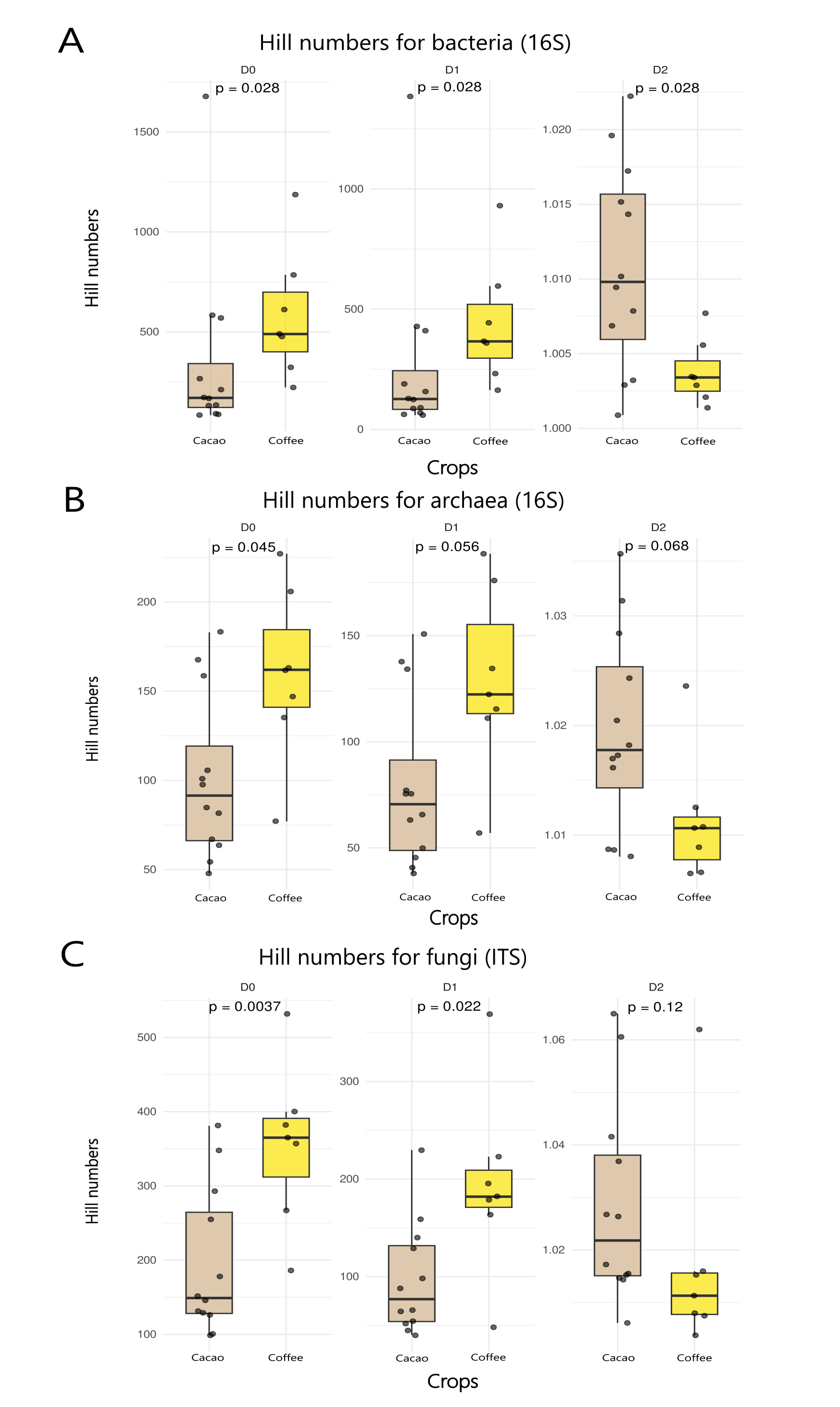


**Figure S10.** Diversity comparisons of the cacao and coffee rhizospheric soil microbiota [i.e., bacteria (A), archaea (B), and fungi (C)] based on Hill numbers for taxon richness (D0), Shannon diversity (D1), and Simpson's dominance (D2).


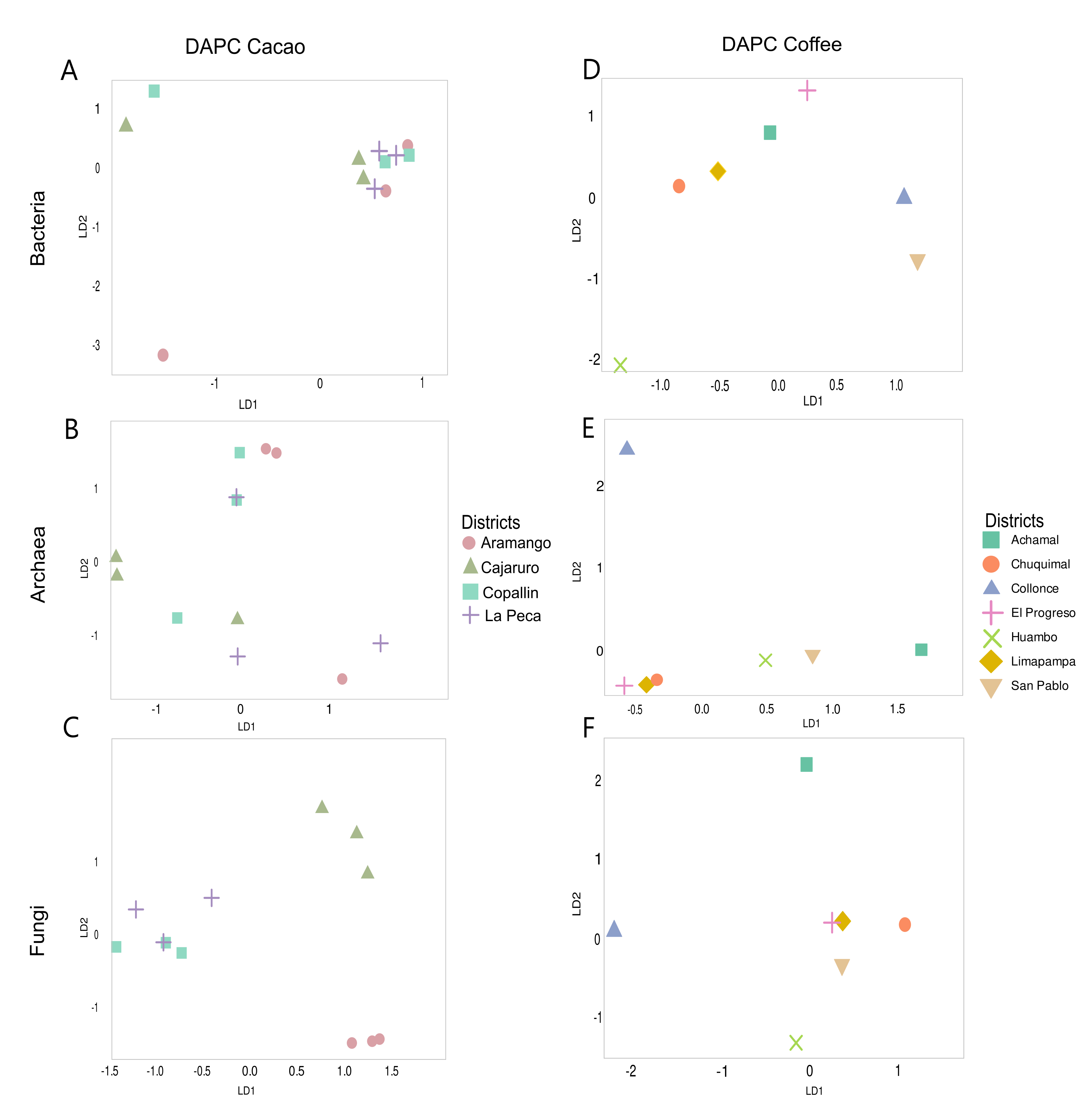


**Figure S11.** DAPC with two linear discriminants (LD1 and LD2) of the microbiota (i.e., bacteria, archaea, and fungi) associated with the rhizospheric soils of cacao (A-C) and coffee (D-F) farms from the Amazonas region.





**Figure S12.** db-RDA-CAP plot representing the variance explained by the influence of physicochemical parameters on the composition of the microbiota for both crops (G-I).

**
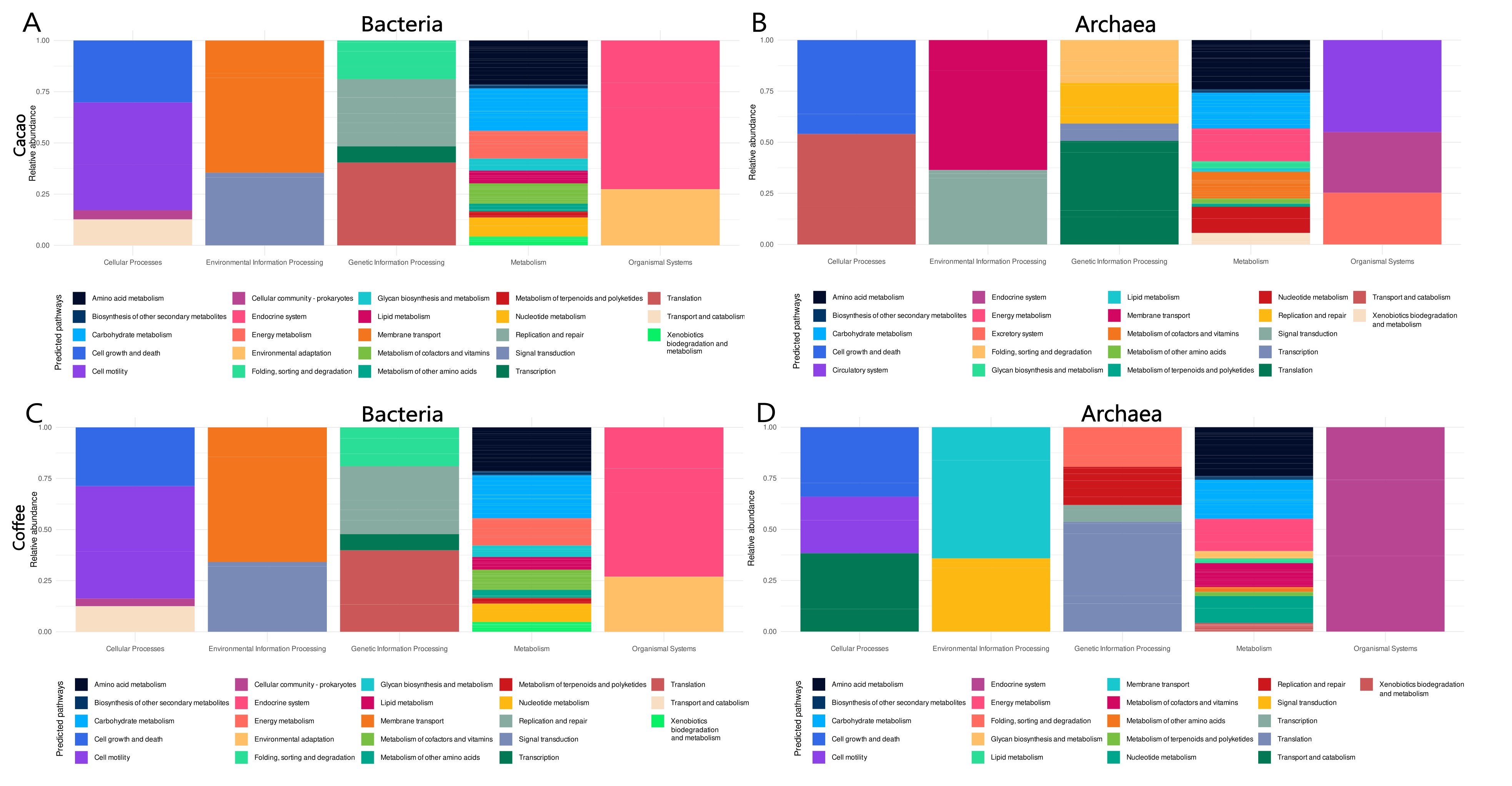
**

**Figure S13.** Predicted pathways for biological functions and subfunctions of bacterial and archaeal microbiota associated with rhizospheric soils of cacao (A, B) and coffee (C, D) farms from the Amazonas region.


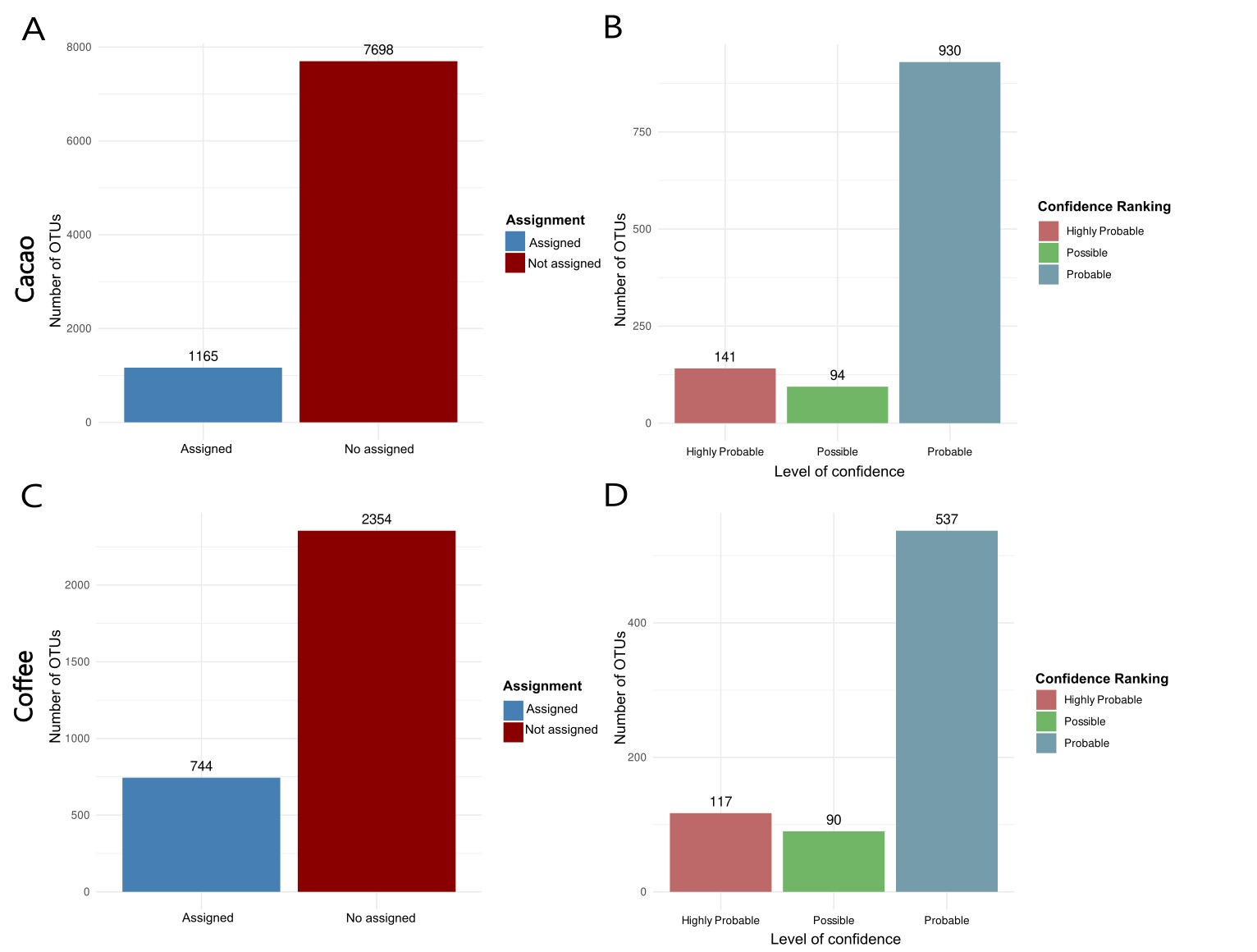


**Figure S14.** Bar chart showing assigned and unassigned ecological guilds, along with confidence rankings, for fungi associated with cacao (A, B) and coffee (C, D) rhizosphere soils in the Amazonas region.


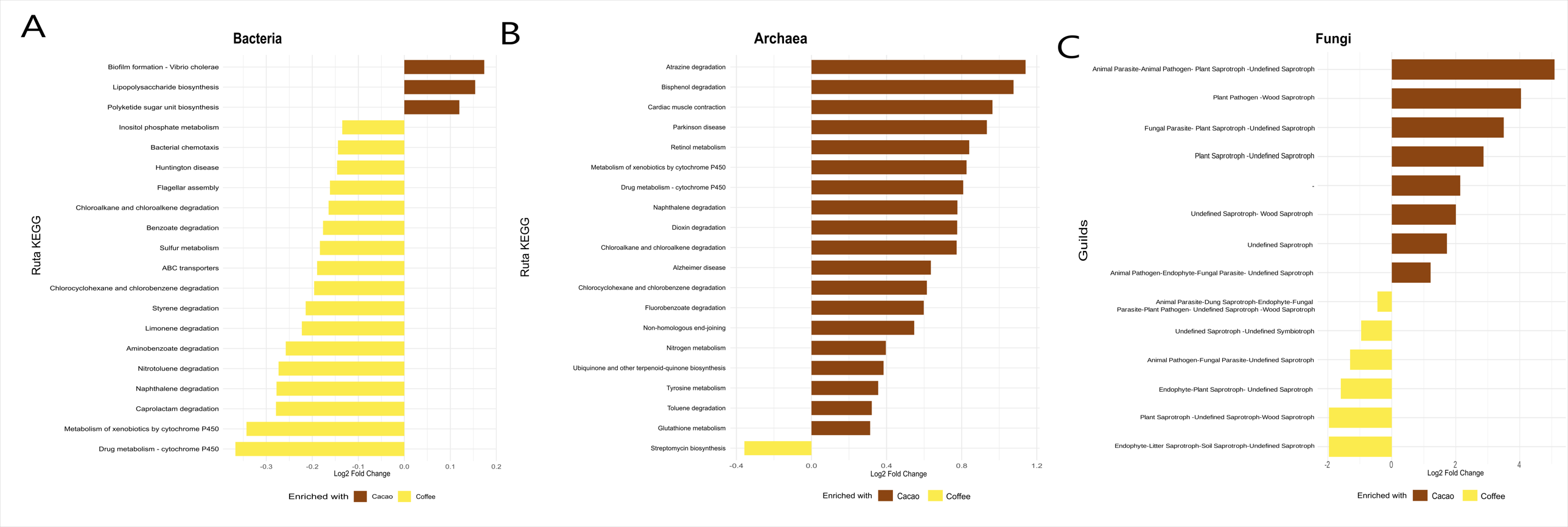


**Figure S15.** Enriched pathways of microbiota associated with cacao and coffee rhizosphere soils in the Amazonas region.
